# Efficacy of WRSs2, a live-attenuated *Shigella sonnei* vaccine, against shigellosis in a controlled human infection model in the USA: a phase 2, double-blind, randomised, placebo-controlled trial

**DOI:** 10.1016/S1473-3099(26)00224-0

**Published:** 2026-06-30

**Authors:** Nadine Rouphael, Shahida Baqar, Michelle Dickey, Christina Quigley, Tena Pham, Josh Adams, Gaurav Kwatra, Sarah Bechnak, Veronica Smith, Erin M Scherer, Daniel S Graciaa, Jill El-Khorazaty, Jamie A Fraser, Susan Heard, Krista Cato, Jorge Mejia-Galvis, Shoshana Barnoy, Lakshmi Chandrasekharan, Chad K Porter, Akamol E Suvarnapunya, Malabi M Venkatesan, Robert W Frenck

**Affiliations:** Hope Clinic of the Emory Vaccine Center, Division of Infectious Diseases, Department of Medicine, Emory University, Decatur, GA, USA; Division of Microbiology and Infectious Diseases, National Institute of Allergy and Infectious Diseases, US National Institutes of Health, Bethesda, MD, USA; Division of Infectious Diseases, Department of Pediatrics, Cincinnati Children’s Hospital Medical Center, University of Cincinnati College of Medicine, Cincinnati, OH, USA; Division of Infectious Diseases, Department of Pediatrics, Cincinnati Children’s Hospital Medical Center, University of Cincinnati College of Medicine, Cincinnati, OH, USA; Division of Infectious Diseases, Department of Pediatrics, Cincinnati Children’s Hospital Medical Center, University of Cincinnati College of Medicine, Cincinnati, OH, USA; Division of Infectious Diseases, Department of Pediatrics, Cincinnati Children’s Hospital Medical Center, University of Cincinnati College of Medicine, Cincinnati, OH, USA; Division of Infectious Diseases, Department of Pediatrics, Cincinnati Children’s Hospital Medical Center, University of Cincinnati College of Medicine, Cincinnati, OH, USA; Hope Clinic of the Emory Vaccine Center, Division of Infectious Diseases, Department of Medicine, Emory University, Decatur, GA, USA; Hope Clinic of the Emory Vaccine Center, Division of Infectious Diseases, Department of Medicine, Emory University, Decatur, GA, USA; Hope Clinic of the Emory Vaccine Center, Division of Infectious Diseases, Department of Medicine, Emory University, Decatur, GA, USA; Hope Clinic of the Emory Vaccine Center, Division of Infectious Diseases, Department of Medicine, Emory University, Decatur, GA, USA; The Emmes Company, Rockville, MD, USA; The Emmes Company, Rockville, MD, USA; The Emmes Company, Rockville, MD, USA; Division of Microbiology and Infectious Diseases, National Institute of Allergy and Infectious Diseases, US National Institutes of Health, Bethesda, MD, USA; Division of Microbiology and Infectious Diseases, National Institute of Allergy and Infectious Diseases, US National Institutes of Health, Bethesda, MD, USA; Walter Reed Army Institute of Research, Silver Spring, MD, USA; Walter Reed Army Institute of Research, Silver Spring, MD, USA; Naval Medical Research Command, Silver Spring, MD, USA; Walter Reed Army Institute of Research, Silver Spring, MD, USA; Walter Reed Army Institute of Research, Silver Spring, MD, USA; Division of Infectious Diseases, Department of Pediatrics, Cincinnati Children’s Hospital Medical Center, University of Cincinnati College of Medicine, Cincinnati, OH, USA

## Abstract

**Background:**

Despite long-standing research, no licensed vaccine exists for shigella, a leading cause of bacterial diarrhoea and dysentery. WRSs2 is a live-attenuated *Shigella sonnei* vaccine candidate which has previously shown safety and immunogenicity. In this trial, we evaluated its safety and efficacy in a controlled human infection model.

**Methods:**

In this phase 2, double-blind, randomised, placebo-controlled trial at two sites in the USA, healthy adults aged 18–49 years were assigned using a site-stratified permuted-block schedule. The original three-arm design allocated participants 1:1:1 to two-dose WRSs2 (10^6^ colony-forming units [CFU]), one-dose placebo followed by one-dose WRSs2 (10^6^ CFU), or two-dose placebo; doses were given 28 days apart. After 69 participants were enrolled, a Data and Safety Monitoring Board (DSMB)-triggered safety review and protocol amendment resulted in subsequent participants being assigned 2:1 to two-dose WRSs2 (5×10^5^ CFU) or placebo. Participants were challenged orally 28 days after the second vaccination with approximately 1·5×10^3^ CFU of *S sonnei* 53G. The primary endpoint was endpoint review committee-adjudicated shigellosis in challenged participants. Safety was assessed in all vaccinated participants. This trial is registered with ClinicalTrials.gov, NCT04242264. The trial is complete.

**Findings:**

Between Oct 11, 2022, and Jan 9, 2024, 108 participants were enrolled, with 22 assigned to two-dose 10^6^ CFU, 26 to two-dose 5×10^5^ CFU, 23 to one-dose 10^6^ CFU, and 37 to placebo. 73 participants underwent challenge (16, 18, 13, and 26 participants in the respective groups). Endpoint review committee-adjudicated shigellosis occurred in three (9%) of 34 participants given pooled two-dose vaccine and 21 (81%) of 26 placebo recipients (vaccine efficacy 89% [95% CI 71–96]; p<0·0001). Six participants had grade 3 post-vaccination adverse events, prompting two DSMB reviews; after the first review, the protocol was amended to reduce the vaccine dose and revise eligibility criteria. There was no change after the second review. No vaccine-related serious adverse events or deaths occurred.

**Interpretation:**

In adults in the USA, WRSs2 provided high-level protection against *S sonnei* shigellosis. Although protection was substantial, the occurrence of a few self-limiting grade 3 adverse events indicates that further optimisation is needed to better define the safety–efficacy balance. These findings support further clinical development of live-attenuated shigella vaccines.

**Funding:**

US National Institutes of Health with pharmaceutical support from the US Department of Defense.

## Introduction

Shigellosis is a major global health challenge,^1^ with the highest burden concentrated in low-income and middle-income countries (LMICs), where inadequate sanitation and poor access to clean water facilitate transmission. Children aged 1–5 years in these regions are disproportionately affected, accounting for most cases and contributing to substantial morbidity and mortality. In LMICs, *Shigella flexneri* is the predominant species, while *Shigella sonnei* is more common in high-income countries, where outbreaks are often linked to international travel, military deployment, childcare settings, and at-risk populations. The increasing global spread of multidrug-resistant shigella strains further underscores the urgent need for effective preventive measures including vaccines.

Various vaccine strategies have been explored, including conjugate,^2^ bioconjugate,^3^ synthetic glycan-based conjugate,^4^ and shigella-based live-attenuated candidates.^1,5,6^ Early conjugate vaccines established proof of concept for O-antigen-based protection, and newer bioconjugate^3^ and synthetic glycan-based conjugate candidates, including Flexyn2a and SF2a-TT15,^4^ have shown promise in clinical studies. Live-attenuated candidates, such as *S flexneri* 2a SC602^7^ and *S sonnei* WRS1,^5,8,9^ provided important proof-of-concept but were constrained by reactogenicity at higher doses or restricted breadth of use. Second-generation live-attenuated candidates, such as WRSs2, incorporate additional gene deletions to improve safety while maintaining immunogenicity.^10,11^ In a dose-escalating phase 1 trial in healthy adults in the USA, a single oral dose of WRSs2 was safe and well tolerated across doses ranging from 10^3^ colony-forming units (CFU) to 10^7^ CFU, with only one case of moderate diarrhoea at the highest dose.^12^ A dose of 10^6^ CFU was considered safe for further evaluation. WRSs2 also induced dose-dependent mucosal and systemic immune responses that correlated with faecal shedding, memory B cells,^13,14^ and functional antibodies.^15^

A controlled human infection model (CHIM) using well characterised shigella challenge strains offers a standardised, efficient platform^16^ for investigating shigella vaccine efficacy and identifying immune correlates of protection, as well as reducing variability, sample size, and time compared with field trials.^17–19^

In this trial, we evaluated the safety, reactogenicity, immunogenicity, and protective efficacy of WRSs2 in a CHIM. The primary objective was to estimate the efficacy of two doses of WRSs2, administered at either 10^6^ CFU or 5×10^5^ CFU, in preventing shigellosis following challenge. Secondary objectives included evaluation of the efficacy of different dosing regimens, further characterisation of the safety profile and reactogenicity of WRSs2, measurement of immune responses post-vaccination and post-challenge by quantifying anti-lipopolysaccharide and anti-WRSs2 IgG and IgA titres, and examination of faecal shedding patterns of *S sonnei*.

## Methods

### Study design

This phase 2, double-blind, randomised, placebo-controlled trial was conducted in healthy adults at two sites in the USA—the Cincinnati Children’s Hospital Medical Center (ethics approval number 2019–0600) and the Hope Clinic of the Emory Vaccine Center Vaccine and Treatment Evaluation Units, selected as they are NIAID Vaccine and Treatment Evaluation Unit sites with established capacity and previous inpatient CHIM studies experience. Originally, participants were assigned 1:1:1 to two-dose WRSs2 (10^6^ CFU), one dose of placebo followed by one dose WRSs2 (10^6^ CFU), or two-dose placebo, with doses 28 days apart. After 69 participants had been enrolled, randomly assigned, and dosed, a Data and Safety Monitoring Board (DSMB)-triggered safety review was convened after grade 3 gastrointestinal adverse events were observed following vaccination. Under protocol amendment 7 (Sept 14, 2023; appendix 1), subsequent participants were assigned 2:1 to two-dose 5×10^5^ CFU WRSs2 or placebo, and eligibility was amended to exclude participants with BMI greater than 40 kg/m^2^ or concurrent use of weight-loss medications. Participants already enrolled before the amendment remained in their originally assigned groups. The final dataset comprised four operational groups: two-dose 10^6^ CFU, two-dose 5×10^5^ CFU, one-dose 10^6^ CFU, and placebo. The challenge phase and primary shigellosis definition were unchanged by the amendment. Participants were admitted to the inpatient unit on day 56 (28 days after scheduled study product administration) and challenged orally on day 57 with approximately 1·5×10^3^ CFU *S sonnei* 53G. A second DSMB-triggered safety pause occurred in January, 2024, after additional grade 3 post-vaccination events; no further protocol changes were made. There was no participant or public involvement in the design, conduct, and reporting of this trial. This trial is registered with ClinicalTrials.gov, NCT04242264. The trial is complete.

### Participants

Eligible participants were healthy adults aged 18–49 years. Eligibility was restricted to participants with low pre-existing immunity to *S sonnei* (defined as a lipopolysaccharide-specific serum IgG ELISA titre ≤1:2500 at screening^19^) to reduce the likelihood that baseline immunity would affect susceptibility to challenge and confound interpretation of vaccine efficacy. After protocol amendment 7, eligibility also required a BMI between 18 kg/m^2^ and 40 kg/m^2^; the upper BMI limit and the prohibition of weight-loss medications were intro duced after the first DSMB-triggered safety review because the earliest grade 3 gastrointestinal events occurred in participants with obesity, concurrent weight-loss agents, or both, prompting a precautionary eligibility modification. Individuals were excluded for immuno compromising conditions, chronic gastrointestinal disease, recent antibiotic use, or pregnancy. Sex assigned at birth was collected by participant self-reporting; gender identity was not collected. Race and ethnicity were self-reported using prespecified categories at enrolment and are reported to describe the study population rather than as biological exposures. Participants provided written informed consent and were followed up for 6 months.

### Randomisation and masking

Participants were randomly assigned to treatment through the Interactive Data Entry System using a site-stratified permuted-block randomisation scheme generated by The Emmes Company (Rockville, MD, USA). Before Amendment 7, allocation was 1:1:1 across the original three groups (two-dose 10^6^ CFU WRSs2, one-dose 10^6^ CFU WRSs2, and placebo). After Amendment 7, the vaccine dose to be given was reduced to 5×10^5^ CFU and subsequent participants were randomly assigned 2:1 between the two-dose 5×10^5^ CFU vaccine and placebo groups. Site investigators and staff enrolled participants after eligibility confirmation and initiated randomisation in the Interactive Data Entry System; they did not have access to the allocation sequence. Treatment assignment was accessible only to unblinded pharmacy personnel and an unblinded research nurse responsible for study product preparation, dispensing, and administration; these personnel were not involved in clinical assessments. All other personnel (participants, investigators, clinical and study staff involved in assessments, and outcome adjudicators) remained masked to treatment assignment. Amendment 7 was implemented after DSMB safety review and was not based on any interim efficacy analysis; participants, investigators, clinical assessors, endpoint adjudicators, and laboratory personnel remained masked to treatment assignment. An independent unblinded biostatistician developed the revised randomisation schedule for the remaining enrolments. Vaccine (in oral saline suspension) and placebo (saline) were administered in identical delivery cups to maintain masking. The final vaccine and placebo preparations appeared visually indistinguishable to the unblinded pharmacist. The success of masking was not formally assessed.

### Study procedures

WRSs2 is a live-attenuated oral *S sonnei* vaccine candidate manufactured under current Good Manufacturing Practice conditions.^10^ The vaccine, derived from the wild-type *S sonnei* Moseley strain, was administered in doses of either 10^6^ CFU or 5×10^5^ CFU, with placebo consisting of normal saline. All doses of placebo and vaccine were given by an unblinded research nurse as 31 mL oral saline suspensions following 150 mL of bicarbonate solution, with a 28-day interval between doses. The *S sonnei* 53G challenge (approximately 1·5×10^3^ CFU) was administered as a 31 mL oral suspension following ingestion of 120 mL of bicarbonate solution on day 57. On day 62 (5 days post-challenge), participants received ciprofloxacin (500 mg twice a day) for 3 days.^18^

During outpatient vaccination, participants were followed up for safety, immunogenicity, and faecal shedding. Participants completed symptom diaries for 7 days after each vaccination to record solicited adverse events, and unsolicited adverse events were followed up to day 56. Serious adverse events (SAEs) were collected in the same way as adverse events, and reported within 24 h of site awareness, to day 180 and followed up until resolution or stabilisation. Blood and stool samples were collected at specified intervals for evaluation of immunogenicity and shedding. Participants who continued to meet eligibility criteria for challenge were admitted to the inpatient unit. Vital signs and physical assessments were performed daily, and stools were monitored for frequency, consistency, weight, and blood.^18^ Daily stool cultures were obtained to assess shedding of the challenge strain. Participants received ciprofloxacin starting on day 62 (5 days post-challenge). Early treatment was initiated if participants met predefined illness criteria or in the judgement of the study physician. Discharge required clinical recovery and two consecutive shigella-negative stool cultures at least 6 h apart. Post-discharge outpatient visits occurred at specified intervals with a final safety telephone follow-up on day 180.

### Outcomes

The primary endpoint was occurrence of shigellosis following challenge, assessed from day 57 to day 63 after challenge in pooled two doses (10^6^ CFU or 5×10^5^ CFU) in WRSs2 recipients versus placebo recipients, defined as: severe diarrhoea; or moderate diarrhoea with fever or with one or more moderate constitutional or enteric symptoms; or dysentery.^20^ An independent Endpoint Review Committee (ERC), blinded to treatment assignment, reviewed primary endpoint data to establish whether participants met the predefined criteria for shigellosis.

Secondary endpoints assessed clinical protection by group, immunogenicity, safety, and *S sonnei* shedding after vaccination and challenge.

Clinical protection against shigellosis was also assessed by individual vaccine-dose group versus placebo.

Safety assessments were conducted systematically. Solicited systemic adverse events, including fever, malaise, headache, and gastrointestinal symptoms, were recorded for 7 days following each vaccination using participant memory aids reviewed by study staff. Related unsolicited adverse events were collected to day 56. SAEs were collected to day 180. Severity was graded using standard toxicity grading scales.^21^

For humoral immunogenicity, serum IgG and IgA responses to *S sonnei* lipopolysaccharide and WRSs2 (a native macromolecular complex composed of *Shigella* lipopolysaccharide and invasion plasmid antigen proteins^22^) were measured using ELISA assays^12^ at days 1, 15, 29, 43, and 56 and post-challenge at days 64, 71, 85, and 113.

Stool specimens were collected to assess WRSs2 faecal shedding on days 4, 8, 15, 29, 32, 36, 43, and 56 and the challenge strain (53G) from day 57 to day 65. Stool samples were cultured and *Shigella* confirmed using colony morphology and biochemical testing. Antigen detection methods complemented culture-based identification for quantification of shedding.

### Statistical analysis

Efficacy was evaluated in the protocol-defined full analysis population, comprising all challenged participants analysed according to their randomised group. The per-protocol population excluded participants with prespecified protocol deviations. Safety analyses included all participants who received at least one vaccination. Immunogenicity and shedding analyses included all vaccinated participants with available data. The original protocol specified a three-arm design (one-dose 10^6^ CFU, two-dose 10^6^ CFU, and placebo) with two primary comparisons versus placebo. Up to 120 participants were to be randomly assigned to yield approximately 90 challenged participants (about 30 per arm), providing around 82% power for each comparison after multiplicity adjustment. At the time of protocol amendment 7, 69 participants had already been randomly assigned and 45 had undergone challenge under the original three-arm design. Amendment 7 discontinued further enrolment into the one-dose 10^6^ CFU group and revised subsequent randomisation to 2:1 between two-dose 5×10^5^ CFU and placebo. The amended primary efficacy comparison was prospectively revised to pooled two-dose vaccine recipients (10^6^ CFU and 5×10^5^ CFU) versus placebo, with a target of 60 challenged participants (two-dose vaccine, n=35; placebo, n=25) providing about 89% power at a two-sided α of 0·05 assuming a 70% placebo attack rate and 57% vaccine efficacy. Recruitment therefore continued because the participants already challenged at the time of amendment were spread across three groups and did not by themselves satisfy the revised two-group target; specifically, participants in the one-dose 10^6^ CFU group no longer contributed to the amended primary analysis. No interim efficacy analysis was performed at the time of amendment. Vaccine efficacy was estimated as 1 minus the relative risk of shigellosis with 95% Wilson CIs. Group-specific efficacy estimates for the two-dose 10^6^ CFU group, the two-dose 5×10^5^ CFU group, and the one-dose 10^6^ CFU group were secondary descriptive analyses and were not powered for formal between-group hypothesis testing. A post-hoc analysis of prevention of severe shigellosis was conducted using methods consistent with the primary vaccine efficacy analysis. No formal subgroup analyses were prespecified, and no imputation was performed for missing data.

### Role of the funding source

The trial was funded by the Division of Microbiology and Infectious Diseases at the National Institute of Allergy and Infectious Diseases, US National Institutes of Health, and the US Department of Defense provided pharmaceutical support for WRSs2 and *S sonnei* 53G. The funder (US National Institutes of Health) held the Investigational New Drug application and was responsible for reviewing safety data and reviewing and approving the study protocol and statistical analysis plan, but had no role in the data analysis or its interpretation.

## Results

**Figure 1:**
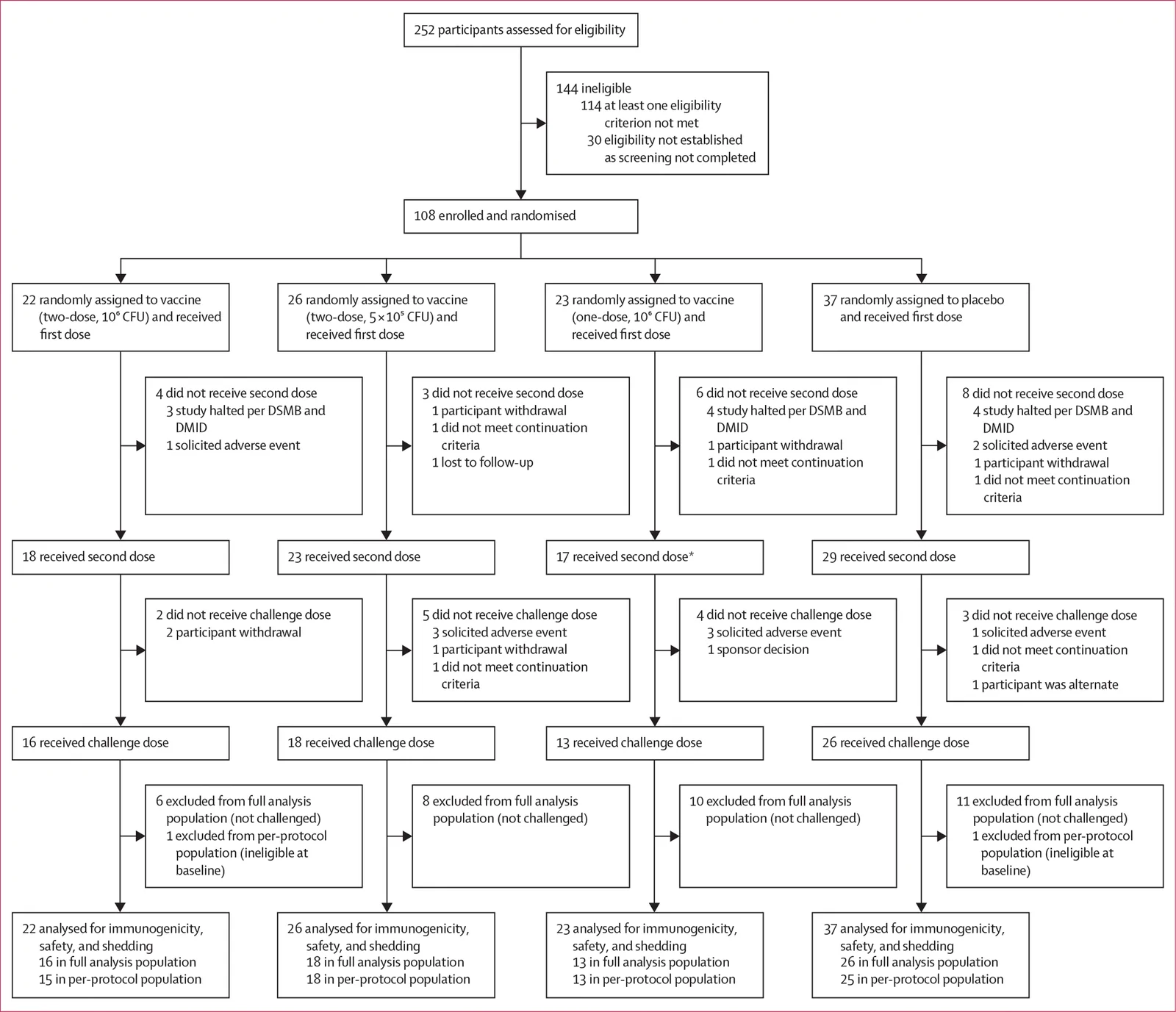
Trial profile The trial began as a three-arm design and, after protocol amendment 7, continued as a two-arm design. CFU=colony-forming units. DMID=Division of Microbiology and Infectious Diseases, National Institute of Allergy and Infectious Diseases, US National Institutes of Health. DSMB=Data and Safety Monitoring Board. ^*^One participant was incorrectly administered placebo instead of WRSs2 at the second scheduled dosing visit.

Between Oct 11, 2022, and Jan 9, 2024, 108 participants were randomly assigned to one of four groups: two-dose 10^6^ CFU (n=22), two-dose 5×10^5^ CFU (n=26), one-dose 10^6^ CFU (n=23), or placebo (n=37; figure 1). Of 108 participants, 87 (81%) received a second vaccination and 73 (84%) of these underwent challenge (full analysis population): 16 (73%) of 22, 18 (69%) of 26, 13 (57%) of 23, and 26 (70%) of 37, respectively. 71 challenged participants comprised the per-protocol population; two challenged participants were excluded from the per-protocol population because eligibility deviations were identified retrospectively during data review—both remained in the full analysis population. Reasons for not proceeding to challenge are shown in figure 1 and included study hold, adverse events, not meeting continuation criteria, participant withdrawal, and other protocol-defined exclusions. 101 (94%) of 108 participants completed safety follow-up to day 180.

Baseline demographic and clinical characteristics of all dosed participants are in appendix 2 (pp 10–11) and of the full analysis population in table 1. Challenged participants had a mean age of 33·7 years (SD 8·7), 38 (52%) were women and 35 (48%) were men. The racial distribution included 46 participants (63%) who identified as White, 20 (27%) Black or African American, and 4 (5%) multiracial. Overall, 4 participants (5%) identified as Hispanic or Latino. The mean BMI was 30·2 kg/m^2^ (SD 7·36). Notably, the two-dose 5×10^5^ CFU group had the lowest mean BMI (28·7 kg/m^2^; SD 5·25), likely reflecting the addition of a BMI eligibility criterion (18–40 kg/m^2^) that coincided with protocol amendment 7 and the dose reduction. The baseline characteristics of the challenged participants closely reflected those of all dosed participants, indicating that the subset who underwent challenge was broadly representative of the enrolled cohort.

The primary vaccine efficacy analysis used the population of all challenged participants and the ERC-determined outcome of shigellosis (table 2). Shigellosis occurred in three (9% [95% CI 3–23]) of 34 participants who received pooled two-dose vaccine (10^6^ or 5×10^5^ CFU), compared with 21 (81% [62–91]) of 26 participants in the placebo group, yielding an estimated vaccine efficacy of 89% (71–96; p<0·0001). The vaccine efficacy in the per-protocol population was also 89% (69–96).

Participants in the one-dose 10^6^ CFU group were analysed separately to the prespecified pooled two-dose vaccine versus placebo comparison because enrolment into the one-dose group was discontinued after protocol amendment 7 and the amended primary analysis was restricted to pooled two-dose vaccine recipients versus placebo recipients. In secondary group-specific analyses, shigellosis occurred in three (19% [95% CI 7–43]) of 16 participants in the two-dose 10^6^ CFU group, 0 (0% [0–18]) of 18 participants in the two-dose 5×10^5^ CFU group, and 0 (0%; 95% CI 0–23%) of 13 participants in the one-dose 10^6^ CFU group. Descriptive statistics for programmatically determined shigellosis by study group and cohort are shown in appendix 2 (p 12).

The severity-specific counts—severe, moderate, and dysentery or other protocol-defined symptoms—were determined programmatically rather than by ERC adjudication. Severe shigellosis occurred in one (3%) of 34 participants in the pooled two-dose groups compared with 18 (69%) of 26 in the placebo group, corresponding to a post-hoc vaccine efficacy estimate of 96% (95% CI 78–99).

Moderate shigellosis occurred in one (6%) of 16 participants in the two-dose 10^6^ CFU group, one (6%) of 18 participants in the two-dose 5×10^5^ CFU group, one (8%) of 13 participants in the one-dose 10^6^ CFU group, and three (12%) of 26 participants in the placebo group. Programmatically determined dysentery, other protocol-defined shigellosis symptoms, or both were most common in placebo recipients (14 [54%] of 26) and were uncommon in vaccine recipients (one [6%] of 16 in the two-dose 10^6^ CFU group; none [0%] of 18 in the two-dose 5×10^5^ CFU group; none [0%] of 13 in the one-dose 10^6^ CFU group). Six placebo recipients received early antibiotics following challenge, two of whom also received intravenous fluids. Rates were consistent across both study sites (appendix 2 p 12).

Solicited adverse events were common after vaccination (table 3). They occurred in 21 (95%) of 22 participants in the two-dose 10^6^ CFU group, 23 (88%) of 26 in the two-dose 5×10^5^ CFU group, 13 (59%) of 22 in the one-dose 10^6^ CFU group, and 30 (79%) of 38 in the placebo group, after any vaccination. Among vaccine recipients, solicited adverse events occurred after the first dose in 86–88% and after the second dose in 69–78%, compared with 67% after the first and 73% after the second placebo doses, respectively. The most frequent solicited adverse events among vaccine recipients were headache (59%), malaise or fatigue (53%), diarrhoea (45%), pain or abdominal cramps (36%), decreased appetite (33%), chills (29%), and arthralgia (27%; table 3). Most events were mild to moderate, peaked within 1–3 days post-vaccination, and resolved without intervention. Although the overall frequency of solicited AEs was comparable between vaccine and placebo recipients, moderate adverse events—including diarrhoea, headache, chills, and fatigue—were more commonly reported in the vaccine groups (appendix 2 p 13), particularly after the first dose. Vaccine-related unsolicited adverse events until day 56 were reported in 9–19% of vaccine recipients and in 0% of placebo recipients (appendix 2 p 14). No vaccine-related or challenge-related SAEs or deaths occurred.

Six (6%) of 108 participants had grade 3 adverse events post-vaccination that triggered two ad hoc DSMB reviews. The first review, in March, 2023, followed grade three gastrointestinal events after 10^6^ CFU vaccination in two participants with obesity and on weight-loss medications. One participant in the one-dose 10^6^ CFU group developed grade 3 nausea and diarrhoea 1 day after vaccination; symptoms resolved within 48 h. After their first dose in the two-dose 10^6^ CFU group, a second participant developed grade 3 diarrhoea, with 5 days of symptoms including loss of appetite and abdominal cramping. After DSMB review, the protocol was amended to reduce the vaccine dose to 5×10^5^ CFU for subsequent two-dose recipients, discontinue further enrolment into the one-dose group, change subsequent randomisation to 2:1 (vaccine:placebo), and exclude participants with BMI greater than 40 kg/m^2^ or concurrent weight-loss medication use. A second safety pause occurred in January, 2024, after four more grade 3 post-vaccination adverse events, including fever, diarrhoea, or both after the first dose. Two cases were associated with intercurrent viral infections (SARS-CoV-2 or norovirus). The other two cases involved self-limited severe diarrhoea after dose 1: one began 5 days post-vaccination and lasted 1 day, and the other began 2 days post-vaccination and lasted 2 days. All affected participants subsequently received and tolerated the second vaccine dose. After review, no further protocol modifications were recommended.

**Figure 2:**
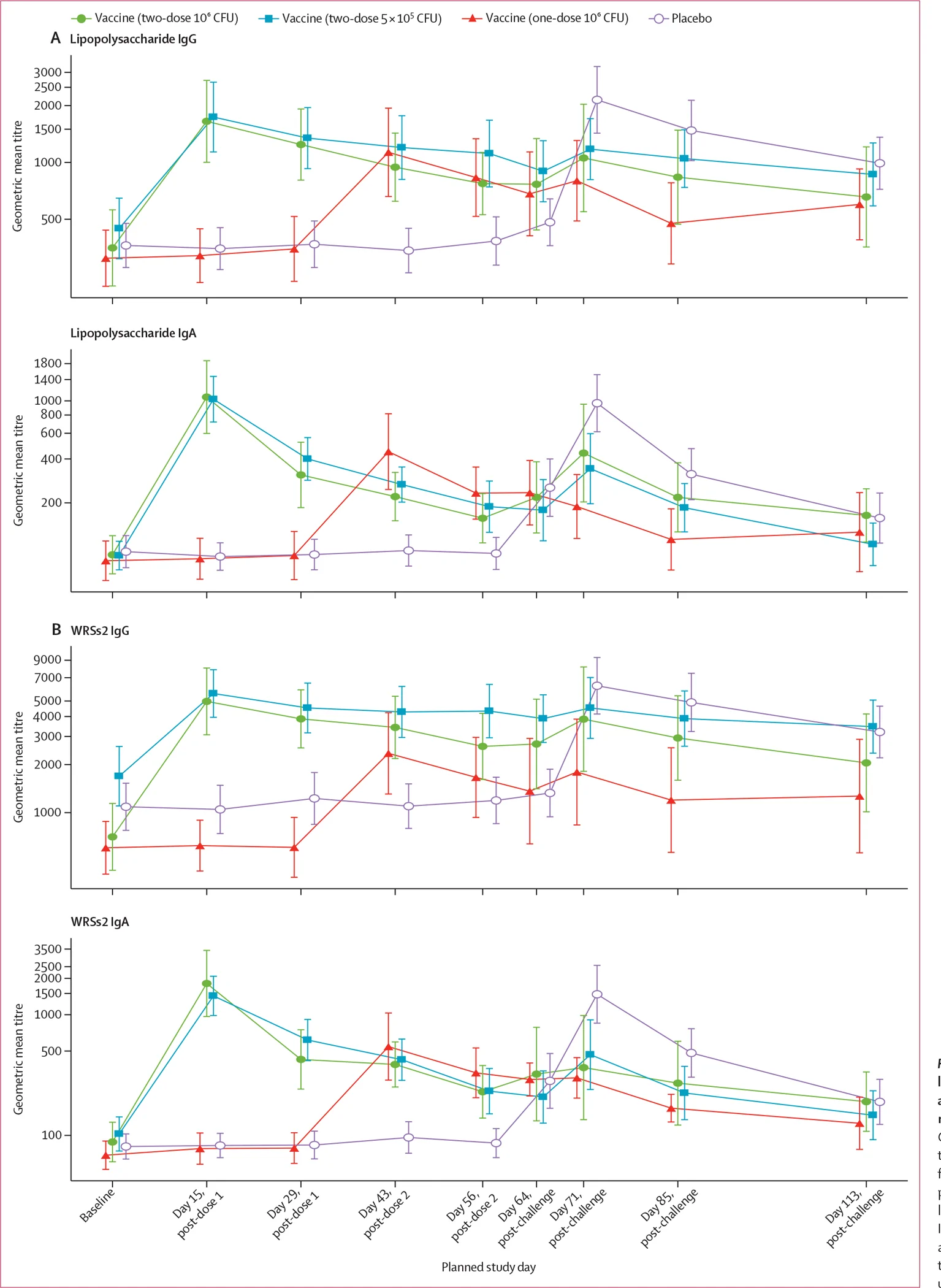
Anti-lipopolysaccharide and anti-WRSs2 IgG and IgA responses Geometric mean titre by timepoint and by study arm for the immunogenicity population for lipopolysaccharide-specific IgG titres and IgA titres (A) and WRSs2 IgG titres and IgA titres (B). CFU=colony-forming units.

**Figure 3:**
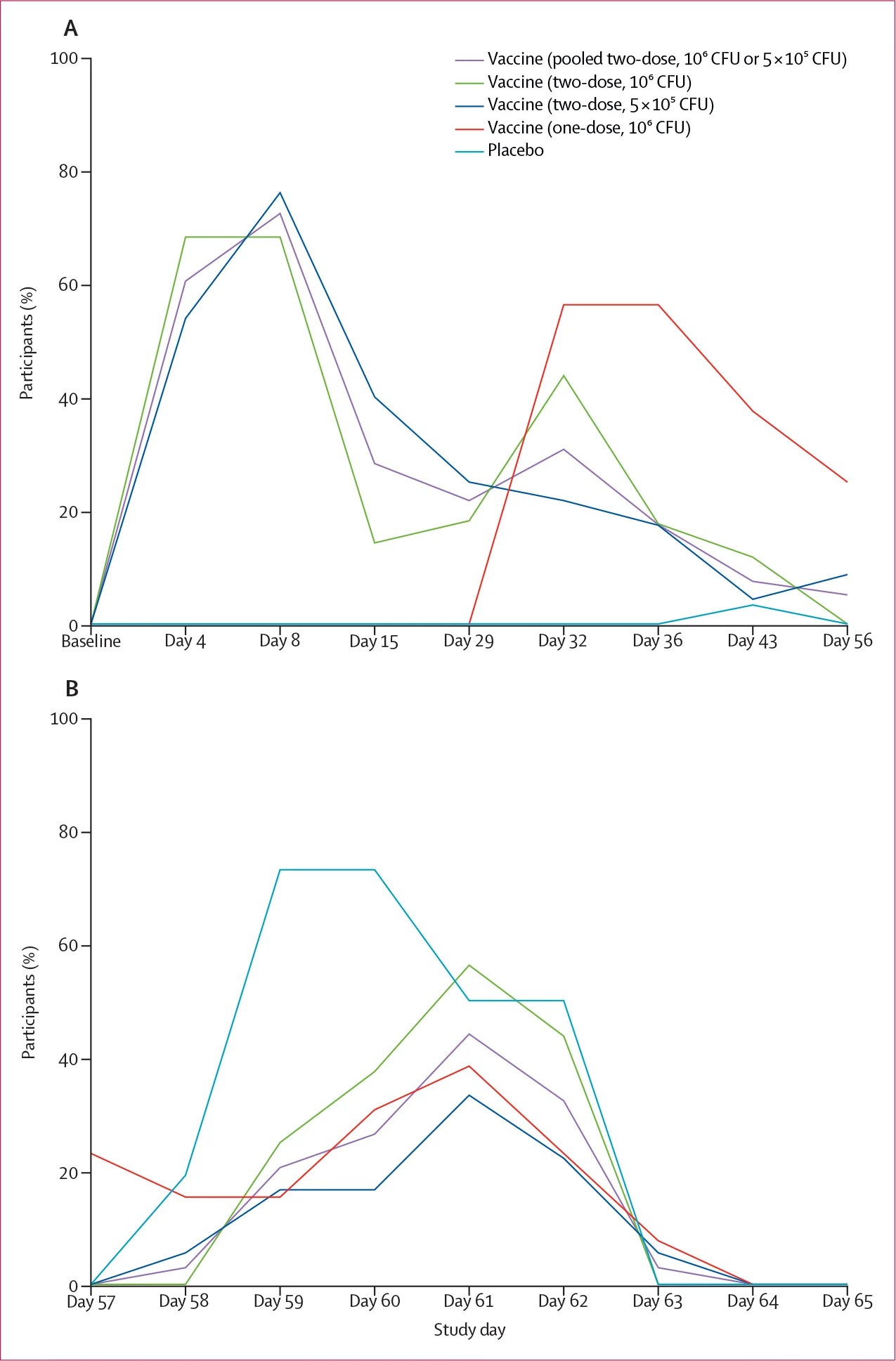
Shedding of *Shigella* in stool post-vaccination and post-challenge (A) Percentage of participants in the shedding analysis population with pre-challenge *Shigella sonnei* shedding by culture by study day and study arm. Study dosing (placebo or vaccine) was administered on day 1 and day 29; for the one-dose WRSs2 group, placebo was administered at the first scheduled dosing visit and WRSs2 at the second. (B) Percentage of participants in the full analysis population with post-challenge shedding by culture and by study day. On day 62, all participants received ciprofloxacin, per protocol. CFU=colony-forming units.

Following vaccination, participants in both two-dose (10^6^ CFU and 5×10^5^ CFU) groups elicited strong antibody responses. By day 15 after the first dose, at least 4-fold rises in anti-lipopolysaccharide IgG were seen in 16 (73%; 95% CI 52–87) of 22 participants in the two-dose 10^6^ CFU group and 19 (76%; 57–89) of 25 participants in the two-dose 5×10^5^ CFU group, with peak geometric mean fold rises (GMFRs) of 5·0 (95% CI 3·4–7·4) and 4·1 (3·1–5·5), respectively. IgA responses were more pronounced, with peak GMFRs of 12·0 (7·0–20·7) and 11·8 (8·4–16·6) and at least 4-fold responses in 19 (86%; 67–95) of 22 participants and 24 (96%; 80–99) of 25 participants in the two-dose groups, respectively (figure 2). WRSs2-specific IgG and IgA responses followed similar kinetics (appendix 2 pp 19–22, 25–26). Few to no serological responses were observed in placebo recipients before challenge. Post-challenge, placebo recipients showed large increases in antibody titres (eg, anti-lipopolysaccharide IgG GMFR 6·1), whereas vaccine recipients had more modest boosts, consistent with previous immunological priming (figure 3; appendix 2 p 15).

Faecal shedding of WRSs2 from post-vaccination until challenge is summarised in figure 3 and appendix 2 (p 27). In the placebo group, one (3%; 95% CI 0–13) of 38 participants shed WRSs2 once, 14 days after the second placebo dose; the mechanism of this isolated finding could not be identified. In the vaccinated population, culture-positive pre-challenge shedding was detected in 43 (90%; 78–95) of 48 participants in the pooled two-dose group and 12 (55%; 35–73) of 22 participants in the one-dose group (appendix 2 p 27). Shedding declined steadily after the first dose in vaccine recipients, with little additional shedding observed following the second dose (figure 3A). In participants with detectable shedding pre-challenge, the mean duration was longest in the two-dose 10^6^ CFU group (21·9 days, SD 14·6, n=14), followed by the two-dose 5×10^5^ CFU group (15·9 days, SD 15·0, n=22), and the one-dose 10^6^ CFU group (13·3 days, SD 10·3, n=11; appendix 2 p 27).

Post-challenge culture-positive shedding of *S sonnei* occurred in 18 (53%; 95% CI 37–69) of 34 participants in the pooled two-dose vaccine groups and eight (62%; 36–82) of 13 participants in the one-dose group, compared with 23 (88%; 71–96) of 26 participants in the placebo group (figure 3B; appendix 2 p 28). After completion of antibiotics, no participant had *Shigella* isolated from their stool. In participants with detectable post-challenge shedding, mean duration was shorter in the pooled two-dose vaccine recipients (2·6 days [SD 1·2, n=18]) and one-dose vaccine recipients (2·5 days [SD 1·3]) than in placebo recipients (3·1 days [SD 1·3, n=23]). Post-challenge, among those with detectable shedding, the colony count was substantially lower in pooled two-dose vaccine recipients (~4·1×10^5^ CFU/g, n=18) compared with placebo (~4·5×10^6^ CFU/g, n=23; appendix 2 p 29). These shedding analyses were secondary descriptive analyses and were not prespecified for formal hypothesis testing.

## Discussion

Although *Shigella* vaccine development has been pursued for more than a century, no licensed vaccine is currently available. In this phase 2 efficacy trial using a CHIM, two oral doses of the live-attenuated *S sonnei* vaccine candidate WRSs2 conferred 89% (95% CI 71–96) protection against shigellosis, with an acceptable safety profile in healthy adults.

Multiple vaccine strategies against shigella—including polysaccharide–protein conjugates, synthetic glycan-based vaccines, and earlier-generation live-attenuated strains—have been explored; few have shown consistent, high-level protection.^1,5,6^ WRSs2 advances the live-attenuated platform by incorporating targeted deletions of senA, senB, and virG(icsA), intended to reduce reactogenicity while preserving immunogenicity. Exploratory group-specific estimates suggested high protection across dose regimens, which was observed across dosing strategies. In a post-hoc analysis, the two-dose regimen prevented 96% (95% CI 78–99) of severe shigellosis cases, underscoring the vaccine’s potential to substantially attenuate disease severity. Reduced faecal shedding after challenge in vaccine recipients suggests the possibility of decreased person-to-person transmission of shigella, although this study was not designed to measure transmission and the public health relevance of this finding will require confirmation in future studies.

WRSs2-associated reactogenicity was generally transient and mild to moderate, with overall frequencies similar between vaccine and placebo recipients, aside from relatively higher rates of diarrhoea, chills, and fever with WRSs2. A small number of grade 3 events prompted DSMB reviews and protocol modifications, including exclusion of participants using weight-loss medications or with elevated BMI and a reduction of the vaccine dose to 5×10^5^ CFU. Two other subsequent grade 3 events were judged as probably unrelated to vaccination (eg, intercurrent SARS-CoV-2 or norovirus infections), whereas two remaining diarrhoeal events were self-limited severe events considered related to vaccination that did not require symptomatic treatment. Importantly, there were no deaths, SAEs, or discontinuations attributed to vaccination, supporting an acceptable safety profile.

Both the 10^6^ CFU and 5×10^5^ CFU regimens elicited robust systemic antibody responses, with at least 4-fold rises in anti-lipopolysaccharide and anti-WRSs2 IgG seen in the majority of vaccine recipients. Antibody titres (lipopolysaccharide and WRSs2, IgG and IgA) peaked around day 15 after the first vaccination and declined thereafter, with no rise after dose 2 indicating no clear boost in immunogenicity or even efficacy from a second dose in this CHIM. The robust induction of both anti-lipopolysaccharide and anti-WRSs2 IgG responses suggests that WRSs2 might engage multiple immune mechanisms, including those associated with opsonophagocytic killing and inhibition of epithelial invasion. Indeed, serum bactericidal and opsonophagocytic assays targeting shigella have shown that lipopolysaccharide-specific antibodies can enhance complement-mediated lysis, and antibodies targeting conserved Ipa proteins (indicated by anti-WRSs2) are capable of inhibiting bacterial invasion of epithelial cells.^23^

The use of a CHIM enabled precise efficacy evaluation within a relatively small sample size in a non-endemic setting.^24^ These findings align with previous CHIM studies, including those investigating Flexyn2a^3^ and earlier live-attenuated vaccine candidates, in showing that vaccine-induced protection against shigellosis can be done in controlled challenge models—although, the magnitude of protection observed in our study with the number of participants was greater than that reported in previous Shigella vaccine challenge studies. In field trials, first-generation shigella conjugate vaccines, such as *S sonnei* O-specific polysaccharide bound to *Pseudomonas aeruginosa* recombinant exoprotein A (*S sonnei*-rEPA), showed 74% protection in young adults (aged 18–22 years) and 71% protection in children aged 3–4 years, but no protection in younger children.^2,25^

Our study has several strengths. First, it is one of the largest shigella CHIM trials to show high efficacy for any shigella vaccine.^19^ Second, as a live oral formulation, WRSs2 offers pragmatic advantages over parenteral vaccines: it is easier to administer, requires less medical training, and could be more practical in resource-limited settings where skilled personnel might be constrained. Third, clinical adjudication of shigellosis cases, combined with daily symptom surveillance, faecal cultures, and quantitative immune assays, ensured comprehensive endpoint assessment. Fourth, the study incorporated adaptive safety oversight, with real-time DSMB review and protocol modifications improving participant safety without compromising data integrity.

The study has limitations. The study population consisted of healthy adults in the USA, and baseline immunity and exposure histories could differ from those in endemic populations, particularly young children. The short interval between the last vaccination and challenge precludes assessment of long-term durability of protection. Additionally, although a CHIM provides a controlled environment for efficacy estimation, it might not fully capture the heterogeneity of natural exposure, co-infections, nutritional influences, force of infection, and environmental enteropathy encountered in endemic settings, particularly in young children who bear the highest burden of shigellosis. The poor performance of some earlier live oral shigella vaccine candidates in such settings underscores the need for dedicated evaluation of WRSs2 in target populations. This study was not designed or powered to define immune correlates of protection, and the very small number of breakthrough shigellosis cases among vaccine recipients limited the interpretability of formal correlate analyses in this report. Functional antibody assays remain of interest for future work; previous studies of WRSs2 have shown serum and faecal bactericidal and opsonophagocytic antibody responses after vaccination.^15^ We have not examined whether post-vaccination diarrhoea was associated with greater humoral immunogenicity in this report, and this remains an important question for future studies of live oral shigella vaccines. An isolated placebo-group culture-positive WRSs2 result was observed during pre-challenge follow-up. We could not establish whether this reflected specimen handling or laboratory artifact, unrecognised exposure, or rare transmission. This uncertainty should be considered when interpreting vaccine-strain shedding findings. Finally, the small number of events in subgroup analyses warrants cautious interpretation of efficacy by dose, and protocol modifications with staged enrolment might have affected comparability across cohorts and should be considered when interpreting pooled efficacy results.

These findings have important implications for shigella vaccine development. Reduced challenge strain shedding among vaccine recipients suggests potential community-level benefits beyond individual protection. The 5×10^5^ CFU dose of WRSs2 seemed to offer strong protection with an acceptable safety profile, supporting its selection for further evaluation. A single-dose regimen could ultimately be attractive for selected use cases such as travellers, but the one-dose efficacy estimate in this study was exploratory, and post-vaccination diarrhoea in nearly half of vaccine recipients would require careful risk–benefit consideration in lower-risk populations. Vaccine-strain shedding was observed after vaccination, particularly after the first dose, but this study did not assess person-to-person transmission of the vaccine strain; the implications for transmission risk should, therefore, be interpreted cautiously and monitored in future studies. WRSs2 contains defined attenuating deletions, reducing the likelihood of simple phenotypic reversion; however, genetic stability and potential compensatory adaptation remain important considerations for live vaccines and warrant continued monitoring through manufacturing controls, genomic characterisation, and future clinical studies.

As shigella strains become increasingly resistant to front-line antimicrobials, effective vaccination remains an urgent global priority. CHIMs will continue to play a pivotal role in down-selecting and optimising candidates before field trials. Building on these results, WRSs2 warrants advancement to trials in endemic populations, including young children. Future studies should assess the durability of immunity, cross-protection against heterologous strains, and potential for co-administration with other paediatric vaccines. When further testing is pursued, dose and dosing schedule could need to be optimised to better define the safety profile—particularly in children, who bear the highest burden of disease. Given the global burden from *S sonnei* and *S flexneri* 2a, 3a, and 6, these findings support continued evaluation of a multivalent candidate comprised of the prevalent circulating serotypes.

In conclusion, WRSs2 is a promising live-attenuated oral *S sonnei* vaccine that provided high-level protection in a rigorous challenge model. These results lay the groundwork for advancing this candidate towards licensure and broader implementation to reduce the global burden of shigellosis.

## Supplementary Material

MMC2

MMC1

## Figures and Tables

**Table 1:** Baseline characteristics of the full analysis population

	Two-dose 10^6^ CFU (n=16)	Two-dose 5 × 10^5^ CFU (n=18)	One-dose 10^6^ CFU (n=13)	Placebo (n=26)

**Sex**				
Male	7 (44%)	10 (56%)	7 (54%)	11 (42%)
Female	9 (56%)	8 (44%)	6 (46%)	15 (58%)
**Ethnicity**				
Not Hispanic or Latino	14 (88%)	17 (94%)	13 (100%)	25 (96%)
Hispanic or Latino	2 (13%)	1 (6%)	0	1 (4%)
**Race**				
Native American or Alaska Native	0	0	0	1 (4%)
Asian	0	1 (6%)	0	0
Black or African American	3 (19%)	3 (17%)	6 (46%)	8 (31%)
White	12 (75%)	13 (72%)	5 (38%)	16 (62%)
Multiracial	0	1 (6%)	2 (15%)	1 (4%)
Not reported	1 (6%)	0	0	0
**Age, years**				
Mean (SD)	32·6 (8·5)	31·8 (7·9)	41·4 (6·0)	31·9 (8·7)
Median (IQR)	29·5 (27·0–40·0)	31·0 (27·0–39·0)	43·0 (41·0–45·0)	31·0 (27·0–35·0)
**BMI, kg/m^2^**				
Mean (SD)	33·36 (9·08)	28·73 (5·25)	29·02 (6·25)	29·80 (7·76)
Median (IQR)	33·00 (29·05–37·65)	26·95 (25·10–31·60)	28·20 (26·70–32·50)	27·80 (24·80–32·80)

Data are n (%) unless otherwise stated. A full list of baseline characteristics for all dosed participants is in appendix 2 (pp 10·11). CFU=colony-forming units.

**Table 2:** Number and proportion of participants with shigellosis and estimated vaccine efficacy post-challenge by shigellosis determination method, analysis population, and study arm vaccine dose

	Participants with shigellosis	Total participants	Proportion of participants with shigellosis (95% Wilson CI)	Estimated vaccine efficacy (95% Wilson CI)	p value*

**Endpoint review committee**					
Full analysis population					
Two-dose 10^6^ or 5 × 10^5^CFU	3	34	0·09 (0·03–0·23)	0·89 (0·71–0·96)	<0·0001
Two-dose 10^6^ CFU	3	16	0·19 (0·07–0·43)	0·77 (0·44–0·92)	··
Two-dose 5 × 10^5^ CFU	0	18	0·00 (0·00–0·18)	1·00 (0·78–1·00)	··
One-dose 10^6^ CFU	0	13	0·00 (0·00–0·23)	1·00 (0·71–1·00)	··
Placebo	21	26	0·81 (0·62–0·91)	··	··
Per-protocol population					
Two-dose 10^6^ or 5 × 10^5^CFU	3	33	0·09 (0·03–0·24)	0·89 (0·69–0·96)	··
Two-dose 10^6^ CFU	3	15	0·20 (0·07–0·45)	0·75 (0·41–0·91)	··
Two-dose 5 × 10^5^ CFU	0	18	0·00 (0·00–0·18)	1·00 (0·77–1·00)	··
One-dose 10^6^ CFU	0	13	0·00 (0·00–0·23)	1·00 (0·71–1·00)	··
Placebo	20	25	0·80 (0·61–0·91)	··	··
**Programmatic definition**					
Full analysis population					
Two-dose 10^6^ or 5 × 10^5^CFU	4	34	0·12 (0·05–0·27)	0·8 (0·66–0·94)	··
Two-dose 10^6^ CFU	3	16	0·19 (0·07–0·43)	0·77 (0·44–0·92)	··
Two-dose 5 × 10^5^ CFU	1	18	0·06 (0·01–0·26)	0·9 (0·67–0·99)	··
One-dose 10^6^ CFU	1	13	0·08 (0·01–0·33)	0·90 (0·57–0·98)	··
Placebo	21	26	0·81 (0·62–0·91)	··	··
Per-protocol population					
Two-dose 10^6^ or 5 × 10^5^CFU	4	33	0·12 (0·05–0·27)	0·8 (0·64–0·94)	··
Two-dose 10^6^ CFU	3	15	0·20 (0·07–0·45)	0·75 (0·41–0·91)	··
Two-dose 5 × 10^5^ CFU	1	18	0·06 (0·01–0·26)	0·93 (0·67–0·99)	··
One-dose 10^6^ CFU	1	13	0·08 (0·01–0·33)	0·90 (0·57–0·98)	··
Placebo	20	25	0·80 (0·61–0·91)	··	··

CFU=colony-forming units. The denominator for proportion is based on the number of participants enrolled in the specified study arm and analysis population.

* p value is shown only for the prespecified primary comparison; individual arm-specific analyses were underpowered and descriptive.

**Table 3:** Number and percentage of participants experiencing solicited events after either vaccination dose, with 95% CIs by symptom, maximum severity, and study arm in the safety population

	Two-dose 10^6^ CFU (n=22)		Two-dose 5 × 10^5^ CFU (n=26)		One-dose 10^6^ CFU (n=22*)		Placebo (n=38*)		All participants (N=108)	
	n (%)	95% CI	n (%)	95% CI	n (%)	95% CI	n (%)	95% CI	n (%)	95% CI

**Any symptom**										
None	1 (5%)	<1–23	3 (12%)	2–30	9 (41%)	21–64	8 (21%)	10–37	21 (19%)	12–28
Mild	10 (45%)	24–68	4 (15%)	4–35	5 (23%)	8–45	17 (45%)	29–62	36 (33%)	25–43
Moderate	10 (45%)	24–68	16 (62%)	41–80	7 (32%)	14–55	12 (32%)	18–49	45 (42%)	32–52
Severe	1 (5%)	<1–23	3 (12%)	2–30	1 (5%)	<1–23	1 (3%)	<1–14	6 (6%)	2–12
**Loss of appetite**										
None	14 (64%)	41–83	15 (58%)	37–77	18 (82%)	60–95	28 (74%)	57–87	75 (69%)	60–78
Mild	7 (32%)	14–55	10 (38%)	20–59	1 (5%)	<1–23	10 (26%)	13–43	28 (26%)	18–35
Moderate	1 (5%)	<1–23	1 (4%)	<1–20	3 (14%)	3–35	0	0–9	5 (5%)	2–10
Severe	0	0–15	0	0–13	0	0–15	0	0–9	0	0–3
**Arthralgia**										
None	14 (64%)	41–83	19 (73%)	52–88	18 (82%)	60–95	30 (79%)	63–90	81 (75%)	66–83
Mild	6 (27%)	11–50	3 (12%)	2–30	1 (5%)	<1–23	7 (18%)	8–34	17 (16%)	9–24
Moderate	2 (9%)	1–29	4 (15%)	4–35	3 (14%)	3–35	1 (3%)	<1–14	10 (9%)	5–16
Severe	0	0–15	0	0–13	0	0–15	0	0–9	0	0–3
**Chills**										
None	17 (77%)	55–92	16 (62%)	41–80	17 (77%)	55–92	33 (87%)	72–96	83 (77%)	68–84
Mild	2 (9%)	1–29	6 (23%)	9–44	3 (14%)	3–35	4 (11%)	3–25	15 (14%)	8–22
Moderate	3 (14%)	3–35	3 (12%)	2–30	2 (9%)	1–29	1 (3%)	<1–14	9 (8%)	4–15
Severe	0	0–15	1 (4%)	<1–20	0	0–15	0	0–9	1 (<1%)	<1–5
**Diarrhoea**										
None	11 (50%)	28–72	11 (42%)	23–63	16 (73%)	50–89	36 (95%)	82->99	74 (69%)	59–77
Mild	10 (45%)	24–68	7 (27%)	12–48	2 (9%)	1–29	2 (5%)	<1–18	21 (19%)	12–28
Moderate	0	0–15	7 (27%)	12–48	3 (14%)	3–35	0	0–9	10 (9%)	5–16
Severe	1 (5%)	<1–23	1 (4%)	<1–20	1 (5%)	<1–23	0	0–9	3 (3%)	<1–8
**Fever**										
None	21 (95%)	77->99	21 (81%)	61–93	21 (95%)	77->99	38 (100%)	91–100	101 (94%)	87–97
Mild	1 (5%)	<1–23	1 (4%)	<1–20	1 (5%)	<1–23	0	0–9	3 (3%)	<1–8
Moderate	0	0–15	2 (8%)	<1–25	0	0–15	0	0–9	2 (2%)	<1–7
Severe	0	0–15	2 (8%)	<1–25	0	0–15	0	0–9	2 (2%)	<1–7
**Headache**										
None	9 (41%)	21–64	8 (31%)	14–52	12 (55%)	32–76	16 (42%)	26–59	45 (42%)	32–52
Mild	5 (23%)	8–45	8 (32%)	14–52	5 (23%)	8–45	14 (37%)	22–54	32 (30%)	21–39
Moderate	8 (36%)	17–59	10 (38%)	20–59	5 (23%)	8–45	7 (18%)	8–34	30 (28%)	20–37
Severe	0	0–15	0	0–13	0	0–15	1 (3%)	<1–14	1 (<1%)	<1–5
**Malaise or fatigue**										
None	10 (45%)	24–68	10 (38%)	20–59	13 (59%)	36–79	20 (53%)	36–69	53 (49%)	39–59
Mild	5 (23%)	8–45	12 (46%)	27–67	7 (32%)	14–55	13 (34%)	20–51	37 (34%)	25–44
Moderate	7 (32%)	14–55	4 (15%)	4–35	2 (9%)	1–29	5 (13%)	4–28	18 (17%)	10–25
Severe	0	0–15	0	0–13	0	0–15	0	0–9	0	0–3
**Myalgia**										
None	14 (64%)	41–83	18 (69%)	48–86	19 (86%)	65–97	30 (79%)	63–90	81 (75%)	66–83
Mild	5 (23%)	8–45	6 (23%)	9–44	1 (5%)	<1–23	6 (16%)	6–31	18 (17%)	10–25
Moderate	3 (14%)	3–35	2 (8%)	<1–25	2 (9%)	1–29	2 (5%)	<1–18	9 (8%)	4–15
Severe	0	0–15	0	0–13	0	0–15	0	0–9	0	0–3
**Nausea**										
None	17 (77%)	55–92	19 (73%)	52–88	16 (73%)	50–89	25 (66%)	49–80	77 (71%)	62–80
Mild	4 (18%)	5–40	6 (23%)	9–44	4 (18%)	5–40	10 (26%)	13–43	24 (22%)	15–31
Moderate	1 (5%)	<1–23	1 (4%)	<1–20	1 (5%)	<1–23	3 (8%)	2–21	6 (6%)	2–12
Severe	0	0–15	0	0–13	1 (5%)	<1–23	0	0–9	1 (<1%)	<1–5
**Pain or abdominal cramps**										
None	16 (73%)	50–89	14 (54%)	33–73	15 (68%)	45–86	26 (68%)	51–82	71 (66%)	56–75
Mild	4 (18%)	5–40	8 (31%)	14–52	3 (14%)	3–35	12 (32%)	18–49	27 (25%)	17–34
Moderate	2 (9%)	1–29	4 (15%)	4–35	4 (18%)	5–40	0	0–9	10 (9%)	5–16
Severe	0	0–15	0	0–13	0	0–15	0	0–9	0	0–3
**Vomiting**										
None	20 (91%)	71–99	25 (96%)	80->99	19 (86%)	65–97	33 (87%)	72–96	97 (90%)	83–95
Mild	2 (9%)	1–29	1 (4%)	<1–20	2 (9%)	1–29	5 (13%)	4–28	10 (9%)	5–16
Moderate	0	0–15	0	0–13	1 (5%)	<1–23	0	0–9	1 (<1%)	<1–5
Severe	0	0–15	0	0–13	0	0–15	0	0–9	0	0–3

Severity is the maximum severity reported post-vaccination for each participant.

* n=22 in the one-dose 10^6^ CFU group and n=38 in the placebo group as one participant randomly assigned to the one-dose group incorrectly received placebo instead of WRSs2 at the second scheduled dosing visit, and this safety table is based on actual study product received.

## Data Availability

Data describing the trial are shared in the ClinicalTrials.gov database at the US National Library of Medicine using the identifier NCT04242264. De-identified individual-participant data and a data dictionary defining the dataset will be made available after publication of the primary outcomes of this trial (NCT04242264). The study protocol, statistical analysis plan, and informed consent form are also available (NCT04242264). Data and supportive documents will be made available on request via email to the US National Institute of Allergy and Infectious Diseases Office of Data Science and Emerging Technologies: datascience@niaid.nih.gov. A data sharing agreement must be signed before data are shared.

## Research in context

### Evidence before this study

We searched PubMed for clinical trials published up to Sept 30, 2025 using the terms “Shigella vaccine” AND (“challenge” OR “efficacy”) without language restrictions. We prioritised randomised trials, field efficacy studies, and controlled human infection model studies. Our search identified several key studies assessing the vaccine efficacy of different *Shigella* vaccine candidates. In earlier phase 3 field trials in young adults aged 18–35 years, *Shigella sonnei* O-specific polysaccharide bound to *Pseudomonas aeruginosa* recombinant exoprotein A (*S sonnei*-rEPA) showed 74% efficacy in a young-adult field trial and a separate early-exposure cohort showed 43% protection. In another phase 3 study conducted in Israel, the overall vaccine efficacy for an *S sonnei* conjugate vaccine was 28%, although substantially higher (71%) in children aged 3–4 years. The Flexyn2a bioconjugate vaccine candidate showed modest protection (30% efficacy) against *Shigella flexneri* 2a in a controlled challenge study in the USA. SC602 also showed substantial protection in a small number of adults in the USA, although later studies in Bangladesh showed low shedding and poor immunogenicity. Modest vaccine efficacy (40%) was observed with the live-attenuated *S sonnei* WRSs1 vaccine in a Thai challenge model. Despite over 100 years of research, no *Shigella* vaccine has been licensed.

### Added value of this study

This study provides, to our knowledge, the first evidence of high-level protection conferred by a live-attenuated *S sonnei* vaccine candidate—WRSs2—evaluated using a controlled human infection model. WRSs2 showed 89% efficacy against *S sonnei* 53G challenge—substantially exceeding efficacy reported in previous *Shigella* vaccine trials. The vaccine was generally well tolerated, with only a few grade 3 adverse events, and elicited robust systemic immune responses while reducing both disease severity and bacterial shedding in vaccinated participants. Conducting the trial in a non-endemic setting enabled precise assessment of vaccine-induced protection. These results support WRSs2 as a strong candidate for further clinical development.

### Implications of all the available evidence

The demonstration of 89% efficacy for WRSs2 marks an important advancement in *Shigella* vaccine development. Although the vaccine conferred high-level protection, some recipients had self-limiting grade 3 adverse events, indicating the need for further optimisation to enhance the safety–efficacy balance. Nonetheless, these findings underscore the potential of further trials for WRSs2, particularly for use in young children and populations in high-burden settings. Amid rising antimicrobial resistance, advancing effective and well tolerated vaccines such as WRSs2 remains crucial for reducing the global burden of diarrhoeal disease.
