# Supplementary material for: Efficacy of WRSs2, a live-attenuated Shigella sonnei vaccine, against shigellosis in a controlled human infection model in the USA: a phase 2, double-blind, randomised, placebo-controlled trial
Source: Lancet Infect Dis. Author manuscript; Available in PMC 2026 Aug 4. (PMC13435326; doi:10.1016/S1473-3099(26)00224-0)
Supplement: MMC2 [file NIHMS2193422-supplement-MMC2.pdf]

THE LANCET
Infectious Diseases

## Supplementary appendix 2

This appendix formed part of the original submission and has been peer reviewed. We post it as supplied by the authors.

Supplement to: Rouphael N, Baqar S, Dickey M, et al. Efficacy of WRSs2, a live-attenuated *Shigella sonnei* vaccine, against shigellosis in a controlled human infection model in the USA: a phase 2, double-blind, randomised, placebo-controlled trial. *Lancet Infect Dis* 2026; published online June 30. https://doi.org/10.1016/S1473-3099(26)00224-0.

**Title Page**

# Efficacy of WRSs2, a Live-Attenuated *Shigella sonnei* vaccine, against Shigellosis in a Controlled Human Infection Model: A Double-Blind, Randomized Trial

**Authors**

Nadine Rouphael, MD[1], Shahida Baqar, PhD[2], Michelle Dickey, NP[3], Christina Quigley, PhD[3], Tena Pham, RN[3], Josh Adams, BS[3], Gaurav Kwatra, PhD, MPH[3], Sarah Bechnak, NP[1], Veronica Smith, NP[1], Erin M. Scherer, PhD, DPhil[1], Daniel S. Graciaa, MD, MPH[1], Jill El-Khorazaty, MS[4], Jamie A. Fraser, MPH[4], Susan Heard, BS[4], Krista Cato, MHA[2], Jorge Mejia-Galvis, MD[2],Shoshana Barnoy PhD [5], Lakshmi Chandrasekharan PhD [5], Chad K. Porter PhD [6], Akamol E. Suvarnapunya, PhD[5], Malabi M. Venkatesan, PhD[5*], Robert W. Frenck Jr, MD[3*] on behalf of DMID 17-0112 Study Group.

1. Hope Clinic of the Emory Vaccine Center, Division of Infectious Diseases, Department of Medicine, Emory University, Atlanta, Georgia, USA
2. Division of Microbiology and Infectious Diseases, National Institute of Allergy and Infectious Diseases, National Institutes of Health, Bethesda, Maryland, USA.
3. Division of Infectious Diseases, Department of Pediatrics, Cincinnati Children's Hospital Medical Center, University of Cincinnati College of Medicine, Cincinnati, Ohio, USA.
4. The Emmes Company, LLC, Rockville, Maryland, USA.
5. Walter Reed Army Institute of Research, Silver Spring, MD, USA.
6. Naval Medical Research Command, Silver Spring, MD, USA

*Joint senior authors

**DMID 17-0112 Study Group Authors:**

Division of Infectious Diseases, Department of Pediatrics, Cincinnati Children's Hospital Medical Center, University of Cincinnati College of Medicine, Cincinnati, Ohio, USA: Rebecca Brady, Carson Caldwell, Maegan Hampton, Monica McNeal, Grant Paulsen.

Hope Clinic of the Emory Vaccine Center, Division of Infectious Diseases, Department of Medicine, Emory University, Atlanta, Georgia, USA: Cassie Grimsley Ackerley, Jill Briggs, Matthew H. Collins, Daniel J. Gromer, Paulina A. Rebolledo, Hady Samaha, Jacob D. Sherman, Ralph Tanios, Jessica Traenkner, Zanthia Wiley.

Division of Microbiology and Infectious Diseases, National Institute of Allergy and Infectious Diseases, National Institutes of Health, Bethesda, Maryland, USA: Lori Newman.

## Supplementary Materials

## Table of Contents

**METHODS** .......... 4

Vaccine Characterization and Manufacturing: .......... 4
Challenge Strain: .......... 4
WRSs2 Vaccine and 53G Challenge Product Preparation: .......... 4
WRSs2 Vaccine and 53G Challenge Product Dose Verification .......... 4

**PRIMARY ENDPOINT** .......... 4

Shigellosis .......... 4
Blood Sample Processing .......... 5
Stool Sample Processing .......... 5
Hemoccult Testing (Inpatient Phase Only): .......... 5
Preparation for Immunoblot and Other Assays: .......... 5
S. sonnei Anti-Lipopolysaccharide (LPS) IgG ELISA .......... 5
S. sonnei Anti-LPS and Anti-Invaplex IgG and IgA ELISA .......... 5
Stool Culture and Agglutination Testing .......... 6

**STATISTICAL ANALYSIS** .......... 6

**SUPPLEMENTAL REFERENCES** .......... 7

**SUPPLEMENTARY FIGURE** .......... 8

Solicited Adverse Events .......... 8

**SUPPLEMENTARY TABLES** .......... 10

Table S1: Baseline Demographic and Clinical Characteristics of All Dosed Participants (Vaccine and Placebo Arms) .......... 10
Table S2: Number and Percentage of Participants Meeting the Primary Endpoint Post-Challenge by Study Arm and Cohort for the Full Analysis Population .......... 12
Table S3: Difference in Proportion of Participants Experiencing Solicited Events Post-Either Vaccination Dose by Study Arm, Safety Population .......... 13
Table S4: Overall Summary of Adverse Events by Study Arm in the Safety Population .......... 14
Table S5: Pre-Challenge LPS-specific IgG GMT, GMFR, and Seroresponse (≥ 4-Fold Rise) Results with 95% Confidence Intervals by Time Point and Study Arm, Immunogenicity Population .......... 15
Table S6: Pre-Challenge LPS-specific IgA GMT, GMFR, and Seroresponse (≥ 4-Fold Rise) Results with 95% Confidence Intervals by Time Point and Study Arm, Immunogenicity Population .......... 17
Table S7: Pre-Challenge Invaplex-specific IgG GMT, GMFR, and Seroresponse (≥ 4-Fold Rise) Results with 95% Confidence Intervals by Time Point and Study Arm, Immunogenicity Population .......... 19
Table S8: Pre-Challenge Invaplex-specific IgA GMT, GMFR, and Seroresponse (≥ 4-Fold Rise) Results with 95% Confidence Intervals by Time Point and Study Arm, Immunogenicity Population .......... 21
Table S9: Post-Challenge LPS-specific IgG GMT, GMFR, and Seroresponse (≥ 4-Fold Rise) Results with 95% Confidence Intervals by Time Point and Study Arm, Immunogenicity Population .......... 23
Table S10: Post-Challenge LPS-specific IgA GMT, GMFR, and Seroresponse (≥ 4-Fold Rise) Results with 95% Confidence Intervals by Time Point and Study Arm, Immunogenicity Population .......... 24
Table S11: Post-Challenge Invaplex-specific IgG GMT, GMFR, and Seroresponse (≥ 4-Fold Rise) Results with 95% Confidence Intervals by Time Point and Study Arm, Immunogenicity Population .......... 25

TABLE S12: POST-CHALLENGE INVAPLEX-SPECIFIC IGA GMT, GMFR, AND SERORESPONSE (≥ 4-FOLD RISE) RESULTS WITH 95% CONFIDENCE INTERVALS BY TIME POINT AND STUDY ARM, IMMUNOGENICITY POPULATION ................................ 26
TABLE S13: SUMMARY OF THE DURATION (DAYS) OF *S. SONNEI* SHEDDING PRE-CHALLENGE BY STUDY ARM FOR THE SHEDDING ANALYSIS POPULATION ........................................................................................................ 27
TABLE S14: SUMMARY OF THE DURATION (DAYS) OF *S. SONNEI* SHEDDING POST-CHALLENGE BY CULTURE AND IMMUNOBLOT BY STUDY ARM FOR THE FULL ANALYSIS POPULATION ...................................................................... 28
TABLE S15: SUMMARY OF MAXIMUM *S. SONNEI* COLONY FORMING UNITS PER GRAM OF STOOL BY IMMUNOBLOT POST-CHALLENGE BY STUDY ARM FOR THE FULL ANALYSIS POPULATION. ....................................................................... 29

**DATA SHARING STATEMENT ........................................................................................................ 30**

## Methods

**Vaccine Characterization and Manufacturing:**
Attenuation of the WRSs2 strain was achieved via sequential deletions of *senA*, *senB*, and *virG (icsA)* using lambda red recombineering, rendering the strain tetracycline-sensitive and immunogenic. The final clinical lot (#1501) was lyophilized at 9·6 × $10^8$ CFU per vial and stored at –80 °C until use.

**Challenge Strain:**
*S. sonnei* strain 53G is a virulent wild-type isolate originally obtained from a child with diarrhea in Tokyo in 1954. The challenge strain used (Lot #1794) was manufactured under cGMP conditions at the Walter Reed Army Institute of Research (WRAIR), lyophilized at 4.0 × $10^9$ CFU per vial, and stored at –80 °C until reconstitution.

**WRSs2 Vaccine and 53G Challenge Product Preparation:**
Each vial of WRSs2 vaccine was thawed on ice for 30 minutes, after which 2 mL of sterile water for injection (SWI) was added. The reconstituted product was kept on ice for 15 minutes with swirling every 5 minutes. Once reconstituted, the vaccine was maintained on ice and used within 2 hours.
WRSs2 was diluted 1:10, 1:100, and 1:1000 in sterile normal saline (0·9% sodium chloride) in 15 mL polypropylene tubes, and optical density (OD600) measurements were taken using a Genesys30 spectrometer (ThermoScientific) to calculate the concentration of vaccine in the vial. Based on the calculated concentration, further dilutions were made in sterile saline to achieve final doses of $10^6$ or 5 × $10^5$ colony-forming units (CFU)/mL. An aliquot was removed and tested by colony count to confirm the administered dose. One milliliter of this preparation was added to 30 mL of sterile saline per participant. After administration, the dosing tube was also tested by colony count to verify the dose. Two 100 µL aliquots were collected from the dosing tube to confirm the presence of the expected mutations in the vaccine strain.
Each vial of the 53G challenge product was thawed on ice for 30 minutes, reconstituted with 2 mL of SWI, and kept on ice for 15 minutes with intermittent swirling. The reconstituted product was maintained on ice and used within 2 hours.
The 53G challenge strain was diluted 1:10, 1:100, and 1:1000 in sterile saline, and OD600 measurements were performed to determine concentration. Based on this, dilutions were prepared to yield a final dose of approximately 1·5 × $10^3$ CFU/mL (range: 1.25 × $10^3$ to 1·75 × $10^3$ CFU/mL). An aliquot was removed for colony counting to confirm the administered dose. One milliliter of the prepared challenge solution was then added to 30 mL of sterile saline per participant. After administration, another aliquot was tested by colony count to verify the delivered dose.

**WRSs2 Vaccine and 53G Challenge Product Dose Verification**
Aliquots of the WRSs2 vaccine (pre- and post-dose) were serially diluted 10-fold to achieve concentrations of 0·5–1 × $10^5$, 0·5–1 × $10^4$, 0·5–1 × $10^3$, or 0·5–1 × $10^2$ CFU/mL in sterile normal saline. After brief vortexing, 0·1 mL of the $10^4$, $10^3$, or $10^2$ dilutions were plated in triplicate on Tryptic Soy Agar (TSA) plates (i.e., 3 plates per dilution) and spread to dryness. Plates were incubated at 37 ± 1 °C for 13 ± 1 hours and either counted immediately or stored at 2–8 °C and enumerated within 24 hours. Both Form I (smooth) and Form II (rough) colony types were counted to assess vaccine quantity and colony morphology.
For the 53G challenge strain, pre- and post-dose aliquots were pulse vortexed briefly, and 0·1 mL of the challenge dose was plated on TSA in triplicate (three plates with 0·1 mL each) and spread to dryness. Incubation and enumeration conditions were identical to those used for WRSs2. Form I and II colonies were also quantified for dose verification and assessment of culture integrity.

## Primary Endpoint (1)

| Shigellosis | Definition |
|---|---|
| 1. Severe Diarrhea | ≥6 stools classified as 3-5 in consistency in 24 hours OR >800 gm stool classified as 3-5 in 24 hours |
| 2. Moderate Diarrhea with additional signs/symptoms | 4-5 stools classified as 3-5 in consistency in 24 hours OR 400-800 gm stools classified as 3-5 in 24 hours with one or more of the following: oral temperature ≥38·0˚C†; ≥1 moderate constitutional/enteric symptom‡; ≥2 episodes of vomiting in 24 hours |

| | |
|---|---|
| 3. Dysentery with additional signs/symptoms | ≥2 stools classified as 3-5 in consistency with gross blood (hemoccult positive) in 24 hours with one or more of the following: oral temperature ≥38·0°C; ≥1 moderate constitutional/enteric symptom; ≥2 episodes of vomiting in 24 hours |

† Confirmed by two separate readings at least five minutes apart
‡ Moderate constitutional/enteric symptoms include nausea, abdominal pain/cramping, myalgia/arthralgia, malaise (does not include anorexia, chills, headache)

**Blood Sample Processing**
Serum: Blood was collected in red-top or serum separator tubes and held at room temperature (15–30 °C) for 30 minutes to 8 hours before centrifugation at ~1000–1300 × g for 10–15 minutes at 4–22 °C, or centrifuged prior to overnight refrigeration. Aliquots were stored at –20 °C or colder.

**Stool Sample Processing**

**Hemoccult Testing (Inpatient Phase Only):** Hemoccult tests were performed during the inpatient phase as part of routine clinical monitoring.

**Preparation for Immunoblot and Other Assays:**
Approximately 2–3 grams of stool were placed into a sterile 15 mL conical tube (Corning) using a spatula. Buffered glycerol saline (BGS; 15.4 mM sodium chloride, 20% glycerol in water) was added at a volume of 2 mL per gram of stool. Samples were vortexed until fully homogenized, centrifuged at 180 × g in a swinging-bucket rotor at 4 °C for 10 minutes, and the supernatant aliquoted into cryovials for storage at –70 ± 10 °C until shipment or long-term storage.

**S. sonnei Anti-Lipopolysaccharide (LPS) IgG ELISA**
ELISA plates (Costar, 9018) were coated with 100 µL of *S. sonnei* LPS (WRAIR, Lot 092308) at 10 µg/mL in carbonate coating buffer (40·6 mM sodium carbonate, 35·7 mM sodium bicarbonate, 3 mM sodium azide; pH 9·8)and sealed overnight at 2–8 °C. The following day, antigen was decanted and plates tapped dry (not washed), then blocked with 300 µL of 5% blotto (w/v skim milk powder in wash buffer: 5·5 M NaCl, 23.5 mM $K_2HPO_4$, 103·4 mM $NaH_2PO_4$, 0·05% Tween-20) for 30 minutes to 2 hours at room temperature.
Thawed participant sera were centrifuged (16,000 × g) and diluted 2-fold in 5% blotto from 1:625 to 1:5000. Negative control serum was diluted 1:200; positive control serum was serially diluted 2-fold from 1:400 to 1:6400. After removing blocking solution and tapping plates dry (without washing), 100 µL of diluted sera or controls were added in duplicate, with 10 wells receiving 5% blotto only, and four wells receiving negative control.
Plates were incubated at room temperature for 2 ± 0·17 hours, washed five times with wash buffer, and incubated with 100 µL/well of alkaline phosphatase-conjugated goat anti-human IgG (SeraCare KPL, 5220-0351), diluted 1:250 in 5% blotto for 1 ± 0·08 hours. Plates were washed five times, followed by an overflow wash using a squirt bottle.
The substrate, pNPP (1 mg/mL in diethanolamine buffer: 10% v/v diethanolamine, 0·77 mM sodium azide, 0·12 mM magnesium chloride; pH 9·8), was prepared 10 minutes prior to use and protected from light. 100 µL per well was added, and plates incubated for 30 minutes at room temperature in the dark. Absorbance was read at 405 nm using a Neo2 plate reader (BioTek).

**S. sonnei Anti-LPS and Anti-Invaplex IgG and IgA ELISA**
ELISA plates (Costar, 9018) were coated with 100 µL of *S. sonnei* LPS (WRAIR, Lot 092308) at 10 µg/mL or Invaplex (WRAIR, Lot PP) at 0·25 µg/mL in carbonate coating buffer (40·6 mM sodium carbonate, 35·7 mM sodium bicarbonate, 3 mM sodium azide; pH 9·8) and sealed overnight at 2–8 °C. The following day, antigen was decanted and plates tapped dry (not washed), then blocked with 300 µL of 5% blotto (w/v skim milk powder in wash buffer: 5·5 M NaCl, 23·5 mM $K_2HPO_4$, 103·4 mM $NaH_2PO_4$, 0·05% Tween-20) for 30 minutes to 2 hours at room temperature.
Thawed participant sera were diluted 2-fold in 5% blotto from 1:100 to 1:3200. Negative control serum was diluted 1:1000 for IgG assays and 1:400 for IgA assays; positive control serum was serially diluted 2-fold from 1:400 to 1:12800 for IgG assays and 1:200 to 1:6400 for IgA assays. After removing blocking solution and tapping plates dry

(without washing), 100 μL of diluted sera or controls were added in duplicate, with 8 wells receiving 5% blotto only, and four wells receiving negative control.
Plates were incubated at room temperature for 2 ± 0·17 hours, washed five times with wash buffer, and incubated with 100 μL/well of alkaline phosphatase-conjugated goat anti-human IgG (SeraCare KPL, 5220-0351) or alkaline phosphatase-conjugated goat anti-human IgA (SeraCare KPL, 5220-0347), diluted 1:250 in 5% blotto for 1 ± 0.08 hours. Plates were washed five times, followed by an overflow wash using a squirt bottle.
The substrate, pNPP (1 mg/mL in diethanolamine buffer: 10% v/v diethanolamine, 0·77 mM sodium azide, 0·12 mM magnesium chloride; pH 9·8), was prepared 10 minutes prior to use and protected from light. 100 μL per well was added, and plates incubated for 30 minutes (IgG assays) or 60 minutes (IgA assays) at room temperature in the dark. Absorbance was read at 405 nm using a SpectraMax ABS Plus plate reader (Molecular Devices). Titers were determined to be the reciprocal of the last dilution above the cutoff value. All samples that were off-scale were retested at a higher dilution until resulted. Any samples that had a titer below 100 were reported as 50.

### Stool Culture and Agglutination Testing

Stool specimens and rectal swabs were cultured for *S. sonnei* within 2 hours of collection when possible, or stored at 2–8 °C for up to 4 hours prior to culture. If bulk stool was unavailable, rectal swabs were directly streaked onto Hektoen Enteric Agar (HEA) plates (Remel, ThermoFisher R01483). For bulk stool, sterile cotton swabs were dipped into at least four different regions of the specimen before streaking onto HEA plates.
HEA plates were incubated at 37 ± 1 °C for 16 ± 2 hours and either read immediately or stored at 2–8 °C for up to 24 hours. Plates with blue-green, non-lactose fermenting colonies were recorded as culture-positive. These colonies underwent confirmatory serogroup D agglutination using *S. sonnei* Type I and II antisera (Denka-Seiken, BD 228371). Yellow-orange lactose-fermenting colonies were considered negative. Plates with overgrowth were re-streaked for isolation.
For agglutination, 10 μL of *S. sonnei* antiserum and 10 μL of 0.85% saline (Hardy Diagnostics) were spotted onto cleaned glass slides. Blue-green colonies were mixed with antiserum or saline and stirred for 5–10 seconds. A reaction was deemed positive if visible agglutination occurred within 5 minutes by eye or under magnification. Positive control HEA plates were streaked with *S. sonnei* Moseley strain (WRAIR Lot #0794); negative controls included *S. flexneri* (WRAIR Lot #1637) or *Escherichia coli* (ATCC 8739). This methodology is consistent with prior protocols(2, 3)

## Statistical Analysis

Core analysis populations and the primary efficacy comparison are described in the main Methods. Briefly, the Full Analysis Population (FAP) population included all challenged participants with complete data for the primary efficacy endpoint and corresponds to the Full Analysis Population defined in the protocol and SAP; the per-protocol population excluded participants with prespecified protocol deviations. The Safety population included all participants who received at least one vaccination, and immunogenicity and shedding analyses included participants with available data for the relevant endpoint.
The original design planned enrolment of up to 120 participants to yield approximately 90 challenged participants across three arms. After Amendment 7, the prospectively defined primary comparison was revised to pooled 2-dose vaccine recipients versus placebo, with a target of approximately 60 challenged participants and about 89% power at two-sided $\alpha$=0·05 assuming a 70% placebo attack rate and 57% vaccine efficacy.
Vaccine efficacy was estimated as 1 minus the relative risk of shigellosis with 95% score confidence intervals. Arm-specific efficacy estimates were secondary and were not powered for formal between-arm comparisons. Sensitivity analyses included the per-protocol population, alternative shigellosis definitions, and site-stratified analyses using Cochran–Mantel–Haenszel methods with Breslow–Day tests for heterogeneity. The severe-shigellosis analysis was post hoc.
Safety and shedding endpoints were analysed descriptively, with no prespecified formal hypothesis testing. Immunogenicity analyses included geometric mean titers, seroconversion rates, and geometric mean fold rises based on log-transformed titers. No interim efficacy analyses were planned, and missing data were not imputed. No multiplicity adjustment was applied in the amended final primary analysis because there was one primary endpoint and one primary comparison.

## Supplemental References


1. MacLennan CA, Aguilar AO, Steele AD. Consensus Report on Shigella Controlled Human Infection Model: Introduction and Overview. Clin Infect Dis. 2019;69(Suppl 8):S577-s9.
2. Talaat KR, Bourgeois AL, Frenck RW, Chen WH, MacLennan CA, Riddle MS, et al. Consensus Report on Shigella Controlled Human Infection Model: Conduct of Studies. Clin Infect Dis. 2019;69(Suppl 8):S580-s90.
3. Frenck RW, Jr., Dickey M, Suvarnapunya AE, Chandrasekaran L, Kaminski RW, Clarkson KA, et al. Establishment of a Controlled Human Infection Model with a Lyophilized Strain of Shigella sonnei 53G. mSphere. 2020;5(5).

## Supplementary Figure

### Solicited Adverse Events

**A-**

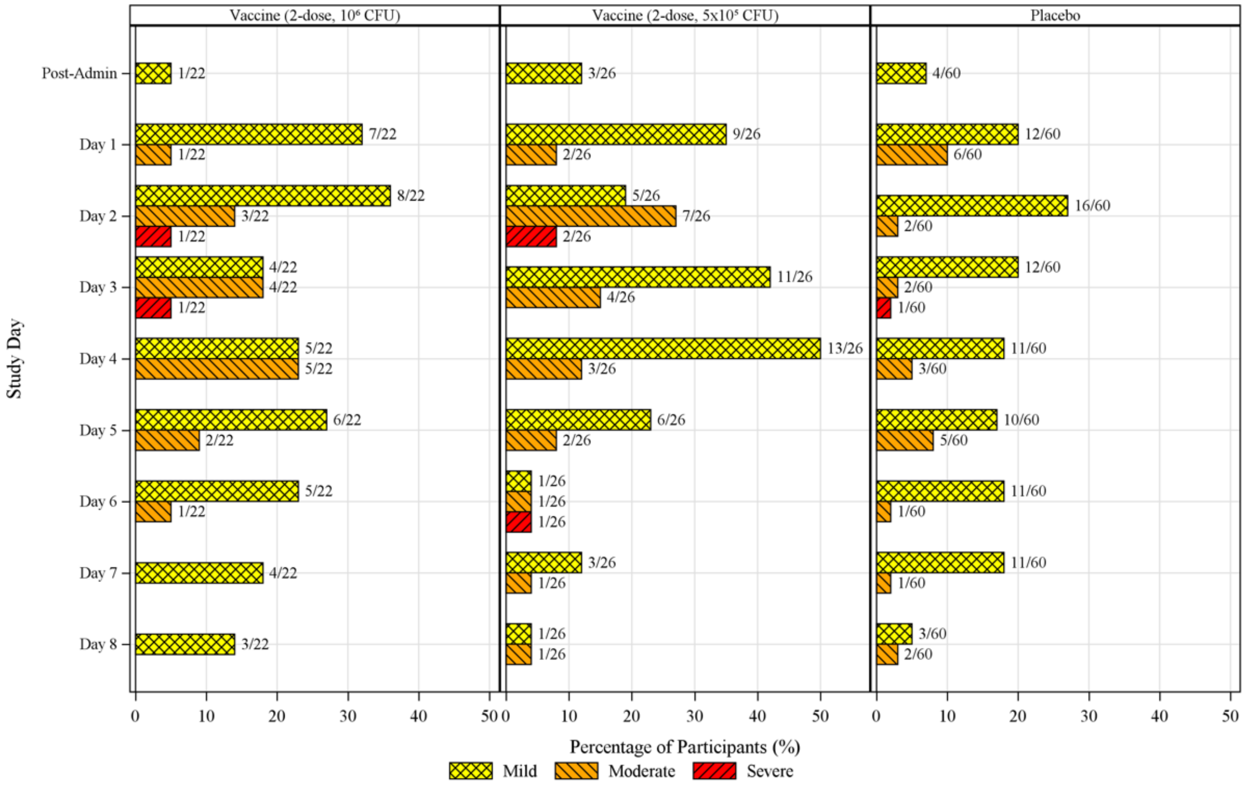

B-

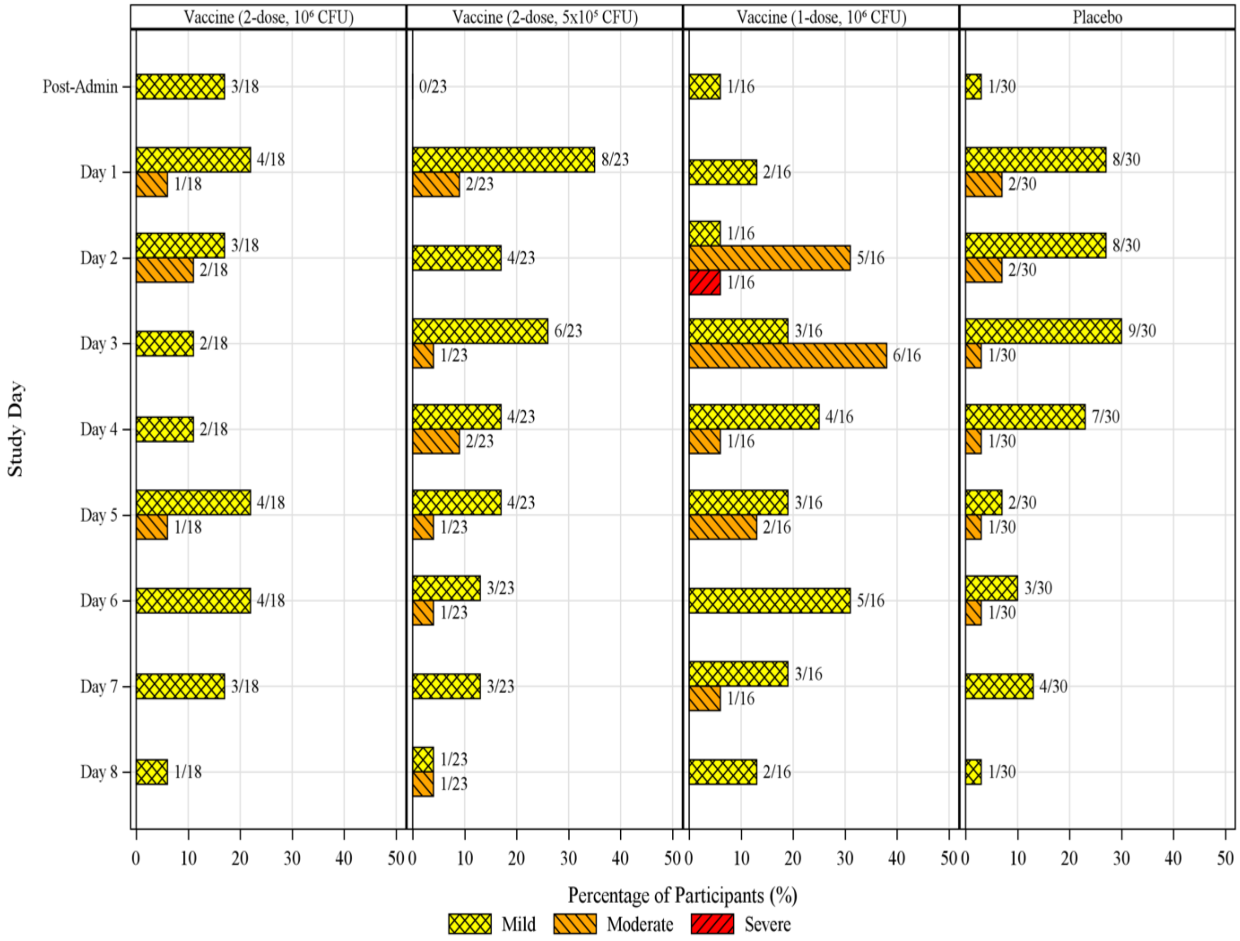


Maximum severity of solicited systemic events post-vaccination Dose 1 (Panel A) and Dose 2 (Panel B) per participant by study day and study arm in the safety population. CFU=colony-forming units.

## Supplementary tables

**Table S1: Baseline Demographic and Clinical Characteristics of All Dosed Participants (Vaccine and Placebo Arms)**

| Variable | Characteristic | 2-dose $10^6$ CFU (N=22) | 2-dose $5x10^5$ CFU (N=26) | 1-dose $10^6$ CFU (N=23) | Placebo (N=37) | All Participants (N=108) |
|---|---|---|---|---|---|---|
| Sex N (%) | Male | 9 (41%) | 15 (58%) | 11 (48%) | 15 (41%) | 50 (46%) |
| | Female | 13(59%) | 11 (42%) | 12 (52%) | 22 (59%) | 58 (54%) |
| Ethnicity N (%) | Not Hispanic or Latino | 20 (91%) | 24 (92%) | 22 (96%) | 33 (89%) | 99 (92%) |
| | Hispanic or Latino | 2 (9%) | 2 (8%) | 1 (4%) | 4 (11%) | 9 (8%) |
| | Not Reported | - | - | - | - | - |
| | Unknown | - | - | - | - | - |
| Race N (%) | American Indian or Alaska Native | - | - | - | 1 (3%) | 1 (<1%) |
| | Asian | - | 1 (4%) | - | - | 1 (<1%) |
| | Native Hawaiian or Other Pacific Islander | - | - | - | - | - |
| | Black or African American | 4 (18%) | 6 (23%) | 9 (39%) | 14 (38%) | 33 (31%) |
| | White | 17 (77%) | 18 (69%) | 12 (52%) | 18 (49%) | 65 (60%) |
| | Multi-Racial | - | 1 (4%) | 2 (9%) | 4 (11%) | 7 (6%) |
| | Unknown | 1 (5%) | - | - | - | 1 (<1%) |
| Age (years) | Mean | 32·0 | 31·8 | 38·3 | 31·2 | 33·0 |
| | Standard Deviation | 7·7 | 7·5 | 8·3 | 7·8 | 8·2 |
| | Median | 29·5 | 31·0 | 41·0 | 31·0 | 31·5 |
| | Q1-Q3 | 27·0, 38·0 | 27·0, 39·0 | 33·0, 45·0 | 25·0, 34·0 | 27·0, 40·0 |
| BMI[a] | Mean | 32·69 | 28·29 | 29·17 | 29·72 | 29·86 |
| | Standard Deviation | 8·33 | 5·17 | 5·93 | 6·89 | 6·74 |
| | Median | 31·70 | 26·95 | 28·40 | 28·60 | 28·80 |

| Variable | Characteristic | 2-dose $10^6$ CFU (N=22) | 2-dose $5x10^5$ CFU (N=26) | 1-dose $10^6$ CFU (N=23) | Placebo (N=37) | All Participants (N=108) |
|---|---|---|---|---|---|---|
| | Q1-Q3 | 27·40, 35.90 | 24·60, 31·60 | 23·20, 33·80 | 26·40, 31·30 | 25·20, 33·85 |

N=number of participants; BMI= Body Mass Index

**Table S2: Number and Percentage of Participants Meeting the Primary Endpoint Post-Challenge by Study Arm and Cohort for the Full Analysis Population**

| Primary endpoint | Dose (CFU) or Placebo | CCHMC, Cohorts, N, n (%) | | | Emory, Cohorts N, n (%) | | | Any N, n (%) |
|---|---|---|---|---|---|---|---|---|
| | | 1 | 2 | 3 | 1 | 2 | 3 | |
| Any Shigellosis | 2-dose $10^6$ | 4, 1 (25) | 5, 1 (20) | - | 4, 0 (0) | 3, 1 (33) | - | 16, 3 (19) |
| | 2-dose $5x10^5$ | - | - | 13, 1 (8) | - | - | 5, 0 (0) | 18, 1 (6) |
| | 1-dose $10^6$ | 3, 0 (0) | 6, 1 (17) | - | 1, 0 (0) | 3, 0 (0) | - | 13, 1, (8) |
| | Placebo | 4, 4, (100) | 6, 6 (100) | 7, 5 (71) | 4, 2 (50) | 2, 1 (50) | 3, 3 (100) | 26, 21 (81) |
| | All Participants | 11, 5 (45) | 17, 8 (47) | 20, 6 (30) | 9, 2 (22) | 8, 2 (25) | 8, 3, (38) | 73, 26, (36) |
| Severe Shigellosis | 2-dose $10^6$ | 4, 0 (0) | 5, 1 (20) | - | 4, 0 (0) | 3, 0 (0) | - | 16, 1 (6) |
| | 2-dose $5x10^5$ | - | - | 13, 0, (0) | - | - | 5, 0 (0) | 18, 0 (0) |
| | 1-dose $10^6$ | 3, 0 (0) | 6, 0 (0) | - | 1, 0 (0) | 3, 0 (0) | - | 13, 0, (0) |
| | Placebo | 4, 3 (75) | 6, 5 (83) | 7, 4 (57) | 4, 2 (50) | 2, 1 (50) | 3, 3 (100) | 26, 18 (69) |
| | All Participants | 11, 3 (27) | 17, 6 (35) | 20, 4 (20) | 9, 2 (22) | 8, 1 (13) | 8, 3 (38) | 73, 19 (26) |
| Moderate Shigellosis | 2-dose $10^6$ | 4, 1 (25) | 5, 0 (0) | - | 4, 0 (0) | 3, 0 (0) | - | 16, 1 (6) |
| | 2-dose $5x10^5$ | - | - | 13, 1 (8) | - | - | 5, 0 (0) | 18, 1 (6) |
| | 1-dose $10^6$ | 3, 0 (0) | 6, 1 (17) | - | 1, 0 (0) | 3, 0 (0) | - | 13, 1 (8) |
| | Placebo | 4, 1 (25) | 6, 1 (17) | 7, 1 (14) | 4, 0 (0) | 2, 0 (0) | 3, 0 (0) | 26, 3 (12) |
| | All Participants | 11, 2 (18) | 17, 2 (12) | 20, 2 (10) | 9, 0 (0) | 8, 0 (0) | 8, 0 (0) | 73, 6, (8) |
| Dysentery and/or Other Shigellosis Symptoms | 2-dose $10^6$ | 4, 0 (0) | 5, 0 (0) | - | 4, 0 (0) | 3, 1 (33) | - | 16, 1 (6) |
| | 2-dose $5x10^5$ | - | - | 13, 0 (0) | - | - | 5, 0 (0) | 18, 0 (0) |
| | 1-dose $10^6$ | 3, 0 (0) | 6, 0 (0) | - | 1, 0 (0) | 3, 0 (0) | - | 13, 0 (0) |
| | Placebo | 4, 2 (50) | 6, 4 (67) | 7, 4, (57) | 4, 1 (25) | 2, 1 (50) | 3, 2 (67) | 26, 14 (54) |
| | All Participants | 11, 2 (18) | 17, 4, (24) | 20, 4 (20) | 9,1 (11) | 8, 2 (25) | 8, 2, (25) | 73, 15 (21) |

N = Number of participants in the Full Analysis Population; n = Number of participants with shigellosis, as determined by programmatic definition.
CCHMC= Cincinnati Children's Hospital Medical Center; Emory=Hope Clinic of the Emory Vaccine Center; CFU= colony-forming units

**Table S3: Difference in Proportion of Participants Experiencing Solicited Events Post-Either Vaccination Dose by Study Arm, Safety Population**

| Symptom | Statistic | 2-dose $10^6$ CFU (N=22) | 2-dose $5x10^5$ CFU (N=26) | 1-dose $10^6$ cfu (N=22) | Placebo (N=38) |
|---|---|---|---|---|---|
| Any Symptom | Proportion (95%CI) | 0·95 (0·77, 1·00) | 0·88 (0·70, 0·98) | 0·59 (0·36, 0·79) | 0·79 (0·63, 0·90) |
| | Difference with Placebo group (95% CI) | 0·17 (-0·05, 0·34) | 0·10 (-0·11, 0·28) | -0·20 (-0·45, 0·05) | - |
| Anorexia/Loss of Appetite | Proportion (95%CI) | 0·36 (0·17, 0·59) | 0·42 (0·23, 0·63) | 0·18 (0·05, 0·40) | 0·26 (0·13, 0·43) |
| | Difference with Placebo group (95% CI) | 0·10 (-0·14, 0·35) | 0·16 (-0·08, 0·40) | -0·08 (-0·29, 0·16) | - |
| Arthralgia | Proportion (95%CI) | 0·36 (0·17, 0·59) | 0·27 (0·12, 0·48) | 0·18 (0·05, 0·40) | 0·21 (0·10, 0·37) |
| | Difference with Placebo group (95% CI) | 0·15 (-0·08, 0·40) | 0·06 (-0·16, 0·29) | -0·03 (-0·23, 0·21) | - |
| Chills | Proportion (95%CI) | 0·23 (0·08, 0·45) | 0·38 (0·20, 0·59) | 0·23 (0·08, 0·45) | 0·13 (0·04, 0·28) |
| | Difference with Placebo group (95% CI) | 0·10 (-0·11, 0·33) | 0·25 (0·03, 0·48) | 0·10 (-0·11, 0·33) | - |
| Diarrhea | Proportion (95%CI) | 0·50 (0·28, 0·72) | 0·58 (0·37, 0·77) | 0·27 (0·11, 0·50) | 0·05 (<0·01, 0·18) |
| | Difference with Placebo group (95% CI) | 0·45 (0·21, 0·67) | 0·52 (0·30, 0·72) | 0·22 (0·03, 0·45) | - |
| Fever | Proportion (95%CI) | 0·05 (<0·01, 0·23) | 0·19 (0·07, 0·39) | 0·05 (<0·01, 0·23) | 0·00 (0·00, 0·09) |
| | Difference with Placebo group (95% CI) | 0·05 (-0·05, 0·23) | 0·19 (0·06, 0·39) | 0·05 (-0·05, 0·23) | - |
| Headache | Proportion (95%CI) | 0·59 (0·36, 0·79) | 0·69 (0·48, 0·86) | 0·45 (0·24, 0·68) | 0·58 (0·41, 0·74) |
| | Difference with Placebo group (95% CI) | 0·01 (-0·25, 0·27) | 0·11 (-0·14, 0·35) | -0·12 (-0·38, 0·15) | - |
| Malaise/Fatigue | Proportion (95%CI) | 0·55 (0·32, 0·76) | 0·62 (0·41, 0·80) | 0·41 (0·21, 0·64) | 0·47 (0·31, 0·64) |
| | Difference with Placebo group (95% CI) | 0·07 (-0·19, 0·33) | 0·14 (-0·11, 0·38) | -0·06 (-0·32, 0·20) | - |
| Myalgia | Proportion (95%CI) | 0·36 (0·17, 0·59) | 0·31 (0·14, 0·52) | 0·14 (0·03, 0·35) | 0·21 (0·10, 0·37) |
| | Difference with Placebo group (95% CI) | 0·15 (-0·08, 0·40) | 0·10 (-0·12, 0·33) | -0·07 (-0·27, 0·16) | - |
| Nausea | Proportion (95%CI) | 0·23 (0·08, 0·45) | 0·27 (0·12, 0·48) | 0·27 (0·11, 0·50) | 0·34 (0·20, 0·51) |
| | Difference with Placebo group (95% CI) | -0·11 (-0·34, 0·15) | -0·07 (-0·30, 0·17) | -0·07 (-0·30, 0·19) | - |
| Pain/Abdominal Cramps | Proportion (95%CI) | 0·27 (0·11, 0·50) | 0·46 (0·27, 0·67) | 0·32 (0·14, 0·55) | 0·32 (0·18, 0·49) |
| | Difference with Placebo group (95% CI) | -0·04 (-0·27, 0·22) | 0·15 (-0·10, 0·39) | 0·00 (-0·24, 0·26) | - |
| Vomiting | Proportion (95%CI) | 0·09 (0·01, 0·29) | 0·04 (<0·01, 0·20) | 0·14 (0·03, 0·35) | 0·13 (0·04, 0·28) |
| | Difference with Placebo group (95% CI) | -0·04 (-0·21, 0·17) | -0·09 (-0·25, 0·08) | 0·00 (-0·18, 0·23) | - |

N = Number of participants in the Safety Population, CI=confidence interval, CFU=colony-forming units

**Table S4: Overall Summary of Adverse Events by Study Arm in the Safety Population**

| | 2-dose $10^6$ CFU (N=22) | | 2-dose $5x10^5$ CFU (N=26) | | 1-dose $10^6$ CFU (N=22) | | Placebo (N=38) | | All Participants (N=108) | |
|---|---|---|---|---|---|---|---|---|---|---|
| **Participants[a] with** | **n** | **%** | **n** | **%** | **n** | **%** | **n** | **%** | **n** | **%** |
| At least one systemic solicited adverse event post-first vaccination | 19 | 86 | 23 | 88 | 12 | 55 | 28 | 74 | 82 | 76 |
| At least one systemic solicited adverse event post-second vaccination | 14 | 78 | 17 | 74 | 11 | 69 | 22 | 73 | 64 | 74 |
| At least one systemic solicited adverse event post-any vaccination | 21 | 95 | 23 | 88 | 13 | 59 | 30 | 79 | 87 | 81 |
| At least one unsolicited adverse event | 16 | 73 | 14 | 54 | 15 | 68 | 25 | 66 | 70 | 65 |
| At least one vaccine-related unsolicited adverse event through 28 days post-last vaccination | 2 | 9 | 5 | 19 | 2 | 9 | - | - | 9 | 8 |
| Mild (Grade 1) | 1 | 5 | 1 | 4 | 1 | 5 | - | - | 3 | 3 |
| Moderate (Grade 2) | - | - | - | - | - | - | - | - | - | - |
| Severe (Grade 3) | 1 | 5 | 4 | 15 | 1 | 5 | - | - | 6 | 6 |
| At least one severe (Grade 3) unsolicited adverse event post-vaccination | 1 | 5 | 4 | 15 | 2 | 9 | 6 | 16 | 13 | 12 |
| Related | 1 | 5 | 4 | 15 | 1 | 5 | 5 | 13 | 11 | 10 |
| Unrelated | - | - | - | - | 1 | 5 | 1 | 3 | 2 | 2 |
| At least one severe (Grade 3) unsolicited adverse event post-challenge | - | - | - | - | - | - | 6 | 16 | 6 | 6 |
| Related | - | - | - | - | - | - | 5 | 13 | 5 | 5 |
| Unrelated | - | - | - | - | - | - | 1 | 3 | 1 | <1 |
| At least one serious adverse event | - | - | - | - | - | - | - | - | - | - |
| At least one related, serious adverse event | - | - | - | - | - | - | - | - | - | - |
| At least one adverse event leading to early termination | - | - | - | - | - | - | - | - | - | - |

N = Number of participants in the Safety Population. The denominator for percentages post-second vaccination is the number of participants who received both vaccinations corresponding to the study arm. CFU= colony-forming units.

[a] Participants are counted once for each category regardless of the number of events.

**Table S5: Pre-Challenge LPS-specific IgG GMT, GMFR, and Seroresponse (≥ 4-Fold Rise) Results with 95% Confidence Intervals by Time Point and Study Arm, Immunogenicity Population**

| Time Point | Statistic | 2-dose $10^6$ CFU (N=22) | 2-dose $5x10^5$ CFU (N=26) | 1-dose $10^6$ cfu (N=22) | Placebo (N=38) | First Dose Placebo All Participants (N=60) |
|---|---|---|---|---|---|---|
| Baseline | n | 22 | 25 | 22 | 36 | 58 |
| | GMT (95% CI) | 352·6 (220·2, 564·6) | 446·9 (307·0, 650·6) | 310·9 (219·3, 440·7) | 363·3 (275·8, 478·5) | 342·4 (277·4, 422·7) |
| Day 15 Post-Dose 1 | n | 22 | 25 | 22 | 36 | 58 |
| | GMT (95% CI) | 1651·2 (995·4, 2739·1) | 1738·8 (1129·2, 2677·5) | 320·8 (229·8, 448·0) | 349·6 (269·0, 454·2) | 338·4 (277·0, 413·3) |
| | GMFR[a] (95% CI) | 4·7 (3·1, 7·0) | 3·9 (2·8, 5·4) | 1·0 (0·9, 1·2) | 1·0 (0·9, 1·1) | 1·0 (0·9, 1·1) |
| | 4-Fold Rise[b] (95% CI) | 73 (52, 87) | 76 (57, 89) | 0 (0, 15) | 0 (0, 10) | 0 (0, 6) |
| Day 29 Pre-Dose 2 | n | 22 | 24 | 20 | 34 | 54 |
| | GMT (95% CI) | 1243·5 (800·7, 1931·3) | 1345·4 (921·3, 1964·8) | 348·2 (232·9, 520·6) | 368·7 (275·8, 492·8) | 361·0 (287·4, 453·3) |
| | GMFR[a] (95% CI) | 3·5 (2·5, 5·1) | 2·8 (2·1, 3·8) | 1·1 (0·9, 1·2) | 1·1 (1·0, 1·2) | 1·1 (1·0, 1·2) |
| | 4-Fold Rise[b] (95% CI) | 59 (39, 77) | 50 (31, 69) | 0 (0, 16) | 0 (0, 10) | 0 (0, 7) |
| Day 43 Post-Dose 2 | n | 21 | 24 | 18 | 35 | - |
| | GMT (95% CI) | 943·5 (618·6, 1439·2) | 1198·6 (807·7, 1778·8) | 1131·4 (656·0, 1951·2) | 341·4 (258·7, 450·6) | - |
| | GMFR[a] (95% CI) | 2·6 (1·8, 3·8) | 2·5 (1·9, 3·4) | 3·7 (1·8, 7·4) | 0·9 (0·8, 1·1) | - |
| | 4-Fold Rise[b] (95% CI) | 43 (24, 63) | 38 (21, 57) | 50 (29, 71) | 0 (0, 10) | - |
| Day 56 Post-Dose 2 | n | 20 | 23 | 18 | 33 | - |
| | GMT (95% CI) | 772·7 (525·1, 1137·2) | 1114·5 (737·5, 1684·1) | 831·4 (514·5, 1343·4) | 383·5 (284·0, 518·1) | - |
| | GMFR[a] (95% CI) | 2·3 (1·5, 3·4) | 2·3 (1·7, 3·1) | 2·7 (1·5, 4·9) | 1·1 (0·9, 1·2) | - |
| | 4-Fold Rise[b] (95% CI) | 40 (22, 61) | 30 (16, 51) | 39 (20, 61) | 0 (0, 10) | - |
| Maximum Titer Post-Vaccination | n | 22 | 25 | 22 | 36 | - |
| | GMT (95% CI) | 1758·6 (1081·3, 2860·1) | 1837·9 (1206·2, 2800·5) | 997·4 (624·4, 1593·2) | 423·8 (323·2, 555·7) | - |
| | GMFR[a] (95% CI) | 5·0 (3·4, 7·4) | 4·1 (3·1, 5·5) | 3·2 (1·8, 5·7) | 1·2 (1·1, 1·3) | - |
| | 4-Fold Rise[bc] (95% CI) | 77 (57, 90) | 80 (61, 91) | 45 (27, 65) | 0 (0, 10) | - |

N = Number of participants in the Immunogenicity Population. The denominator for percentages post-dose 2 is the number of participants who received both vaccinations corresponding to the study arm.

[a] GMFR represents the geometric mean fold rise in IgG antibody compared to pre-dose 1.

[b] 4-Fold Rise represents the percentage of participants with at least a 4-Fold Rise in IgG antibody compared to pre-dose 1.

[c] 4-Fold Rise at Maximum Titer Post-Vaccination represents the percentage of participants with at least a 4-Fold Rise in IgG antibody at the maximum titer value post-dose compared to pre-dose 1.
CFU= colony-forming units

**Table S6: Pre-Challenge LPS-specific IgA GMT, GMFR, and Seroresponse (≥ 4-Fold Rise) Results with 95% Confidence Intervals by Time Point and Study Arm, Immunogenicity Population**

| Time Point | Statistic | 2-dose $10^6$ CFU (N=22) | 2-dose $5x10^5$ cfu (N=26) | 1-dose $10^6$ cfu (N=22) | Placebo (N=38) | First Dose Placebo All Participants (N=60) |
|---|---|---|---|---|---|---|
| Baseline | n | 22 | 25 | 22 | 36 | 58 |
| | GMT (95% CI) | 88·2 (64·7, 120·1) | 87·1 (68·9, 110·0) | 80·2 (58·2, 110·5) | 92·6 (71·3, 120·3) | 87·7 (72·0, 106·8) |
| Day 15 Post-Dose 1 | n | 22 | 25 | 22 | 36 | 58 |
| | GMT (95% CI) | 1062·3 (593·6, 1901·1) | 1026·7 (710·4, 1483·9) | 82·8 (59·4, 115·3) | 85·7 (68·5, 107·4) | 84·6 (70·5, 101·4) |
| | GMFR[a] (95% CI) | 12·0 (7·0, 20·7) | 11·8 (8·4, 16·6) | 1·0 (0·9, 1·2) | 0·9 (0·8, 1·1) | 1·0 (0·9, 1·1) |
| | 4-Fold Rise[b] (95% CI) | 86 (67, 95) | 96 (80, 99) | 0 (0, 15) | 0 (0, 10) | 0 (0, 6) |
| Day 29 Pre-Dose 2 | n | 22 | 24 | 20 | 34 | 54 |
| | GMT (95% CI) | 310·9 (184·1, 525·1) | 400·0 (283·2, 564·9) | 87·1 (59·1, 128·3) | 88·5 (69·0, 113·5) | 88·0 (71·7, 108·0) |
| | GMFR[a] (95% CI) | 3·5 (2·2, 5·6) | 4·5 (3·3, 6·1) | 1·0 (0·8, 1·3) | 1·0 (0·8, 1·2) | 1·0 (0·8, 1·2) |
| | 4-Fold Rise[b] (95% CI) | 55 (35, 73) | 75 (55, 88) | 0 (0, 16) | 3 (1, 15) | 2 (0, 10) |
| Day 43 Post-Dose 2 | n | 21 | 24 | 18 | 35 | - |
| | GMT (95% CI) | 220·8 (149·5, 326·2) | 267·0 (200·7, 355·1) | 449·0 (245·0, 822·8) | 94·2 (73·1, 121·5) | - |
| | GMFR[a] (95% CI) | 2·4 (1·7, 3·5) | 3·0 (2·4, 3·8) | 5·7 (2·9, 11·0) | 1·0 (0·8, 1·2) | - |
| | 4-Fold Rise[b] (95% CI) | 43 (24, 63) | 50 (31, 69) | 72 (49, 88) | 3 (1, 15) | - |
| Day 56 Post-Dose 2 | n | 20 | 23 | 18 | 33 | - |
| | GMT (95% CI) | 156·9 (105·4, 233·5) | 188·3 (124·6, 284·7) | 233·3 (153·5, 354·7) | 90·0 (69·3, 116·9) | - |
| | GMFR[a] (95% CI) | 1·7 (1·2, 2·5) | 2·1 (1·5, 2·9) | 2·9 (2·0, 4·4) | 1·0 (0·8, 1·1) | - |
| | 4-Fold Rise[b] (95% CI) | 30 (15, 52) | 26 (13, 46) | 56 (34, 75) | 0 (0, 10) | - |
| Maximum Titer Post-Vaccination | n | 22 | 25 | 22 | 36 | - |
| | GMT (95% CI) | 1062·3 (593·6, 1901·1) | 1026·7 (710·4, 1483·9) | 341·7 (186·3, 626·9) | 110·1 (85·6, 141·6) | - |
| | GMFR[a] (95% CI) | 12·0 (7·0, 20·7) | 11·8 (8·4, 16·6) | 4·3 (2·3, 7·8) | 1·2 (1·0, 1·4) | - |
| | 4-Fold Rise[bc] (95% CI) | 86 (67, 95) | 96 (80, 99) | 59 (39, 77) | 6 (2, 18) | - |

N = Number of participants in the Immunogenicity Population. The denominator for percentages post-dose 2 is the number of participants who received both vaccinations corresponding to the study arm.

[a] GMFR represents the geometric mean fold rise in IgA antibody compared to pre-dose 1.

[b] 4-Fold Rise represents the percentage of participants with at least a 4-Fold Rise in IgA antibody compared to pre-dose 1.

[c] 4-Fold Rise at Maximum Titer Post-Vaccination represents the percentage of participants with at least a 4-Fold Rise in IgA antibody at the maximum titer value post-dose compared to pre-dose 1.
CFU= colony-forming units

**Table S7: Pre-Challenge Invaplex-specific IgG GMT, GMFR, and Seroresponse (≥ 4-Fold Rise) Results with 95% Confidence Intervals by Time Point and Study Arm, Immunogenicity Population**

| Time Point | Statistic | 2-dose $10^6$ CFU (N=22) | 2-dose $5x10^5$ CFU (N=26) | 1-dose $10^6$ CFU (N=22) | Placebo (N=38) | First Dose Placebo All Participants (N=60) |
|---|---|---|---|---|---|---|
| Baseline | n | 22 | 25 | 22 | 36 | 58 |
| | GMT (95% CI) | 705·3 (432·3, 1150·6) | 1691·2 (1093·1, 2616·6) | 602·5 (409·1, 887·3) | 1088·6 (769·0, 1541·2) | 869·8 (667·6, 1133·3) |
| Day 15 Post-Dose 1 | n | 22 | 25 | 22 | 36 | 58 |
| | GMT (95% CI) | 4974·1 (3051·4, 8108·3) | 5571·5 (3924·5, 7909·7) | 621·8 (427·8, 903·7) | 1047·5 (733·9, 1495·1) | 859·5 (660·2, 1118·9) |
| | GMFR[a] (95% CI) | 7·1 (4·4, 11·3) | 3·3 (2·3, 4·7) | 1·0 (0·8, 1·3) | 1·0 (0·8, 1·1) | 1·0 (0·9, 1·1) |
| | 4-Fold Rise[b] (95% CI) | 73 (52, 87) | 52 (33, 70) | 0 (0, 15) | 0 (0, 10) | 0 (0, 6) |
| Day 29 Pre-Dose 2 | n | 22 | 24 | 20 | 34 | 54 |
| | GMT (95% CI) | 3865·9 (2524·7, 5919·6) | 4525·5 (3137·9, 6526·7) | 606·3 (390·8, 940·5) | 1227·5 (838·3, 1797·3) | 945·3 (703·0, 1271·1) |
| | GMFR[a] (95% CI) | 5·5 (3·7, 8·1) | 2·7 (2·1, 3·6) | 0·9 (0·7, 1·2) | 1·1 (1·0, 1·3) | 1·1 (0·9, 1·2) |
| | 4-Fold Rise[b] (95% CI) | 73 (52, 87) | 46 (28, 65) | 0 (0, 16) | 0 (0, 10) | 0 (0, 7) |
| Day 43 Post-Dose 2 | n | 21 | 24 | 18 | 35 | - |
| | GMT (95% CI) | 3418·4 (2166·2, 5394·4) | 4271·5 (2934·6, 6217·4) | 2351·6 (1298·6, 4258·2) | 1098·3 (790·8, 1525·2) | - |
| | GMFR[a] (95% CI) | 4·7 (3·2, 7·0) | 2·6 (1·9, 3·5) | 3·8 (2·3, 6·3) | 1·0 (0·8, 1·2) | - |
| | 4-Fold Rise[b] (95% CI) | 67 (45, 83) | 42 (24, 61) | 50 (29, 71) | 3 (1, 15) | - |
| Day 56 Post-Dose 2 | n | 20 | 23 | 18 | 33 | - |
| | GMT (95% CI) | 2599·2 (1602·8, 4215·1) | 4325·5 (2922·4, 6402·2) | 1662·8 (926·5, 2984·4) | 1192·4 (846·8, 1679·0) | - |
| | GMFR[a] (95% CI) | 4·0 (2·6, 6·1) | 2·6 (2·0, 3·5) | 2·7 (1·6, 4·5) | 1·1 (0·9, 1·3) | - |
| | 4-Fold Rise[b] (95% CI) | 55 (34, 74) | 39 (22, 59) | 50 (29, 71) | 3 (1, 15) | - |
| Maximum Titer Post-Vaccination | n | 22 | 25 | 22 | 36 | - |
| | GMT (95% CI) | 5467·2 (3373·0, 8861·6) | 5571·5 (3924·5, 7909·7) | 1873·0 (1078·0, 3254·4) | 1371·6 (970·3, 1938·8) | - |
| | GMFR[a] (95% CI) | 7·8 (4·9, 12·3) | 3·3 (2·3, 4·7) | 3·1 (2·0, 4·9) | 1·3 (1·1, 1·4) | - |
| | 4-Fold Rise[bc] (95% CI) | 77 (57, 90) | 52 (33, 70) | 45 (27, 65) | 3 (0, 14) | - |

N = Number of participants in the Immunogenicity Population. The denominator for percentages post-dose 2 is the number of participants who received both vaccinations corresponding to the study arm.

[a] GMFR represents the geometric mean fold rise in IgG antibody compared to pre-dose 1.

[b] 4-Fold Rise represents the percentage of participants with at least a 4-Fold Rise in IgG antibody compared to pre-dose 1.
[c] 4-Fold Rise at Maximum Titer Post-Vaccination represents the percentage of participants with at least a 4-Fold Rise in IgG antibody at the maximum titer value post-dose compared to pre-dose 1.
CFU= colony-forming units

**Table S8: Pre-Challenge Invaplex-specific IgA GMT, GMFR, and Seroresponse (≥ 4-Fold Rise) Results with 95% Confidence Intervals by Time Point and Study Arm, Immunogenicity Population**

| Time Point | Statistic | 2-dose $10^6$ CFU (N=22) | 2-dose $5x10^5$ CFU (N=26) | 1-dose $10^6$ CFU (N=22) | Placebo (N=38) | First Dose Placebo All Participants (N=60) |
|---|---|---|---|---|---|---|
| Baseline | n | 22 | 25 | 22 | 36 | 58 |
| | GMT (95% CI) | 88·2 (59·9, 129·8) | 102·8 (73·5, 143·8) | 68·5 (51·8, 90·7) | 80·9 (63·0, 103·9) | 76·0 (63·2, 91·3) |
| Day 15 Post-Dose 1 | n | 22 | 25 | 22 | 36 | 58 |
| | GMT (95% CI) | 1814·9 (956·3, 3444·3) | 1432·0 (974·8, 2103·7) | 77·7 (57·1, 105·8) | 82·5 (64·8, 105·1) | 80·6 (67·1, 97·0) |
| | GMFR[a] (95% CI) | 20·6 (11·3, 37·4) | 13·9 (9·2, 21·1) | 1·1 (0·9, 1·4) | 1·0 (0·9, 1·1) | 1·1 (1·0, 1·2) |
| | 4-Fold Rise[b] (95% CI) | 91 (72, 97) | 96 (80, 99) | 0 (0, 15) | 0 (0, 10) | 0 (0, 6) |
| Day 29 Pre-Dose 2 | n | 22 | 24 | 20 | 34 | 54 |
| | GMT (95% CI) | 426·0 (239·4, 758·0) | 616·9 (412·2, 923·1) | 78·5 (58·0, 106·2) | 83·2 (63·2, 109·6) | 81·4 (66·7, 99·5) |
| | GMFR[a] (95% CI) | 4·8 (2·8, 8·3) | 6·3 (4·2, 9·7) | 1·1 (0·9, 1·4) | 1·0 (0·9, 1·2) | 1·1 (0·9, 1·2) |
| | 4-Fold Rise[b] (95% CI) | 64 (43, 80) | 88 (69, 96) | 0 (0, 16) | 3 (1, 15) | 2 (0, 10) |
| Day 43 Post-Dose 2 | n | 21 | 24 | 18 | 35 | - |
| | GMT (95% CI) | 387·0 (249·2, 601·1) | 423·8 (282·9, 634·8) | 544·3 (284·2, 1042·6) | 96·1 (70·4, 131·1) | - |
| | GMFR[a] (95% CI) | 4·3 (2·7, 6·8) | 4·4 (2·9, 6·7) | 7·7 (3·6, 16·3) | 1·2 (0·9, 1·5) | - |
| | 4-Fold Rise[b] (95% CI) | 76 (55, 89) | 63 (43, 79) | 72 (49, 88) | 9 (3, 22) | - |
| Day 56 Post-Dose 2 | n | 20 | 23 | 18 | 33 | - |
| | GMT (95% CI) | 229·7 (137·8, 383·1) | 232·5 (149·4, 361·9) | 329·9 (203·2, 535·7) | 86·3 (64·8, 115·0) | - |
| | GMFR[a] (95% CI) | 2·5 (1·7, 3·8) | 2·4 (1·5, 3·8) | 4·7 (2·9, 7·4) | 1·0 (0·8, 1·3) | - |
| | 4-Fold Rise[b] (95% CI) | 45 (26, 66) | 39 (22, 59) | 72 (49, 88) | 6 (2, 20) | - |
| Maximum Titer Post-Vaccination | n | 22 | 25 | 22 | 36 | - |
| | GMT (95% CI) | 1814·9 (956·3, 3444·3) | 1472·3 (1005·5, 2155·9) | 426·0 (221·0, 821·3) | 110·1 (80·5, 150·6) | - |
| | GMFR[a] (95% CI) | 20·6 (11·3, 37·4) | 14·3 (9·5, 21·6) | 6·2 (3·2, 12·0) | 1·4 (1·1, 1·7) | - |
| | 4-Fold Rise[bc] (95% CI) | 91 (72, 97) | 96 (80, 99) | 68 (47, 84) | 11 (4, 25) | - |

: N = Number of participants in the Immunogenicity Population. The denominator for percentages post-dose 2 is the number of participants who received both vaccinations corresponding to the study arm.

[a] GMFR represents the geometric mean fold rise in IgA antibody compared to pre-dose 1.
[b] 4-Fold Rise represents the percentage of participants with at least a 4-Fold Rise in IgA antibody compared to pre-dose 1.
[c] 4-Fold Rise at Maximum Titer Post-Vaccination represents the percentage of participants with at least a 4-Fold Rise in IgA antibody at the maximum titer value post-dose compared to pre-dose 1.
CFU= colony-forming units

**Table S9: Post-Challenge LPS-specific IgG GMT, GMFR, and Seroresponse (≥ 4-Fold Rise) Results with 95% Confidence Intervals by Time Point and Study Arm, Immunogenicity Population**

| Time Point | Statistic | 2-dose $10^6$ CFU (N=16) | 2-dose $5x10^5$ CFU (N=18) | 1-dose $10^6$ CFU (N=13) | Placebo (N=26) |
|---|---|---|---|---|---|
| Day 64 | n | 16 | 18 | 13 | 26 |
| | GMT (95% CI) | 766·1 (436·0, 1346·1) | 898·0 (615·0, 1311·1) | 681·7 (406·4, 1143·7) | 482·1 (360·1, 645·3) |
| | GMFR[a] (95% CI) | 1·1 (0·9, 1·5) | 0·9 (0·7, 1·1) | 0·9 (0·6, 1·3) | 1·4 (1·0, 1·8) |
| | 4-Fold Rise[b] (95% CI) | 6 (1, 28) | 0 (0, 18) | 0 (0, 23) | 12 (4, 29) |
| Day 71 | n | 15 | 18 | 12 | 26 |
| | GMT (95% CI) | 1055·6 (544·7, 2045·8) | 1175·8 (805·7, 1715·9) | 800·0 (486·8, 1314·8) | 2145·3 (1419·7, 3241·5) |
| | GMFR[a] (95% CI) | 1·6 (1·1, 2·3) | 1·2 (0·9, 1·5) | 1·0 (0·7, 1·4) | 6·1 (4·0, 9·4) |
| | 4-Fold Rise[b] (95% CI) | 13 (4, 38) | 6 (1, 26) | 0 (0, 24) | 77 (58, 89) |
| Day 85 | n | 16 | 18 | 12 | 26 |
| | GMT (95% CI) | 835·4 (467·9, 1491·5) | 1047·5 (732·7, 1497·5) | 475·7 (288·2, 785·3) | 1477·0 (1016·0, 2147·3) |
| | GMFR[a] (95% CI) | 1·2 (1·0, 1·6) | 1·0 (0·8, 1·3) | 0·6 (0·4, 1·0) | 4·2 (2·9, 6·1) |
| | 4-Fold Rise[b] (95% CI) | 6 (1, 28) | 6 (1, 26) | 0 (0, 24) | 62 (43, 78) |
| Day 113 | n | 14 | 18 | 12 | 26 |
| | GMT (95% CI) | 656·3 (354·2, 1215·8) | 864·0 (584·9, 1276·4) | 599·3 (386·5, 929·4) | 990·2 (716·0, 1369·4) |
| | GMFR[a] (95% CI) | 1·0 (0·7, 1·4) | 0·9 (0·7, 1·1) | 0·8 (0·4, 1·5) | 2·8 (2·1, 3·8) |
| | 4-Fold Rise[b] (95% CI) | 7 (1, 31) | 0 (0, 18) | 8 (1, 35) | 46 (29, 65) |
| Maximum Titer Post-Challenge | n | 16 | 18 | 13 | 26 |
| | GMT (95% CI) | 1233·8 (709·0, 2147·1) | 1175·8 (805·7, 1715·9) | 843·8 (528·9, 1346·2) | 2145·3 (1419·7, 3241·5) |
| Peak-Fold Rise | n | 16 | 18 | 13 | 26 |
| | GMFR[a] (95% CI) | 1·8 (1·3, 2·6) | 1·2 (0·9, 1·5) | 1·1 (0·8, 1·6) | 6·1 (4·0, 9·4) |
| | 4-Fold Rise[bc] (95% CI) | 25 (10, 49) | 6 (1, 26) | 8 (1, 33) | 77 (58, 89) |

N = Number of participants in the Immunogenicity Population who received challenge.
[a] GMFR represents the geometric mean fold rise in IgG antibody compared to pre-challenge (Day 56).
[b] 4-Fold Rise represents the percentage of participants with at least a 4-Fold Rise in IgG antibody compared to pre-challenge (Day 56).
[c] 4-Fold Rise at Peak-Fold Rise represents the percentage of participants with at least a 4-Fold Rise in IgG antibody at the maximum titer value post-dose compared to pre-challenge (Day 56).
CFU= colony-forming units

**Table S10: Post-Challenge LPS-specific IgA GMT, GMFR, and Seroresponse (≥ 4-Fold Rise) Results with 95% Confidence Intervals by Time Point and Study Arm, Immunogenicity Population**

| Time Point | Statistic | 2-dose $10^6$ CFU (N=16) | 2-dose $5x10^5$ CFU (N=18) | 1-dose $10^6$ CFU (N=13) | Placebo (N=26) |
|---|---|---|---|---|---|
| Day 64 | n | 16 | 18 | 13 | 26 |
| | GMT (95% CI) | 218·1 (123·3, 385·7) | 178·2 (109·0, 291·1) | 234·7 (139·9, 393·7) | 254·2 (160·3, 403·3) |
| | GMFR[a] (95% CI) | 1·3 (0·9, 1·8) | 1·0 (0·7, 1·3) | 1·0 (0·6, 1·7) | 3·1 (2·0, 4·9) |
| | 4-Fold Rise[b] (95% CI) | 13 (3, 36) | 0 (0, 18) | 8 (1, 33) | 50 (32, 68) |
| Day 71 | n | 15 | 18 | 12 | 26 |
| | GMT (95% CI) | 438·7 (201·2, 956·6) | 342·9 (195·6, 601·2) | 188·8 (113·0, 315·3) | 964·1 (609·4, 1525·3) |
| | GMFR[a] (95% CI) | 2·6 (1·5, 4·7) | 1·9 (1·0, 3·3) | 0·7 (0·4, 1·3) | 11·9 (7·0, 20·4) |
| | 4-Fold Rise[b] (95% CI) | 47 (25, 70) | 39 (20, 61) | 8 (1, 35) | 92 (76, 98) |
| Day 85 | n | 16 | 18 | 12 | 26 |
| | GMT (95% CI) | 218·1 (125·3, 379·6) | 185·2 (125·4, 273·5) | 112·2 (68·7, 183·4) | 314·7 (208·6, 474·6) |
| | GMFR[a] (95% CI) | 1·3 (1·0, 1·7) | 1·0 (0·7, 1·5) | 0·5 (0·4, 0·8) | 3·9 (2·7, 5·6) |
| | 4-Fold Rise[b] (95% CI) | 6 (1, 28) | 11 (3, 33) | 0 (0, 24) | 65 (46, 81) |
| Day 113 | n | 14 | 18 | 12 | 26 |
| | GMT (95% CI) | 164·1 (107·0, 251·7) | 103·9 (73·7, 146·6) | 126·0 (67·0, 237·1) | 157·3 (105·1, 235·5) |
| | GMFR[a] (95% CI) | 0·9 (0·7, 1·2) | 0·6 (0·4, 0·8) | 0·5 (0·3, 0·7) | 1·9 (1·5, 2·5) |
| | 4-Fold Rise[b] (95% CI) | 0 (0, 22) | 0 (0, 18) | 0 (0, 24) | 27 (14, 46) |
| Maximum Titer Post-Challenge | n | 16 | 18 | 13 | 26 |
| | GMT (95% CI) | 436·2 (211·3, 900·4) | 400·0 (229·7, 696·5) | 275·4 (166·7, 454·9) | 1044·4 (697·0, 1564·9) |
| Peak-Fold Rise | n | 16 | 18 | 13 | 26 |
| | GMFR[a] (95% CI) | 2·6 (1·5, 4·4) | 2·2 (1·3, 3·6) | 1·2 (0·7, 2·0) | 12·9 (8·2, 20·3) |
| | 4-Fold Rise[bc] (95% CI) | 44 (23, 67) | 39 (20, 61) | 15 (4, 42) | 92 (76, 98) |

N = Number of participants in the Immunogenicity Population who received challenge.

[a] GMFR represents the geometric mean fold rise in IgA antibody compared to pre-challenge (Day 56).

[b] 4-Fold Rise represents the percentage of participants with at least a 4-Fold Rise in IgA antibody compared to pre-challenge (Day 56).

[c] 4-Fold Rise at Peak-Fold Rise represents the percentage of participants with at least a 4-Fold Rise in IgA antibody at the maximum titer value post-dose compared to pre-challenge (Day 56).

CFU= colony-forming units

**Table S11: Post-Challenge Invaplex-specific IgG GMT, GMFR, and Seroresponse (≥ 4-Fold Rise) Results with 95% Confidence Intervals by Time Point and Study Arm, Immunogenicity Population**

| Time Point | Statistic | 2-dose $10^6$ cfu (N=16) | 2-dose $5x10^5$ CFU (N=18) | 1-dose $10^6$ CFU (N=13) | Placebo (N=26) |
|---|---|---|---|---|---|
| Day 64 | n | 16 | 18 | 13 | 26 |
| | GMT (95% CI) | 2690·9 (1399·4, 5174·1) | 3879·4 (2731·5, 5509·8) | 1363·5 (632·8, 2938·0) | 1327·6 (935·4, 1884·4) |
| | GMFR[a] (95% CI) | 1·1 (0·9, 1·5) | 0·9 (0·8, 1·1) | 0·9 (0·7, 1·0) | 1·2 (0·9, 1·6) |
| | 4-Fold Rise[b] (95% CI) | 6 (1, 28) | 0 (0, 18) | 0 (0, 23) | 15 (6, 34) |
| Day 71 | n | 15 | 18 | 12 | 26 |
| | GMT (95% CI) | 3849·7 (1799·7, 8234·6) | 4525·5 (2896·3, 7071·2) | 1795·9 (831·2, 3880·6) | 6231·6 (4114·5, 9438·1) |
| | GMFR[a] (95% CI) | 1·6 (1·0, 2·5) | 1·1 (0·9, 1·4) | 0·9 (0·7, 1·3) | 5·7 (3·6, 9·0) |
| | 4-Fold Rise[b] (95% CI) | 27 (11, 52) | 6 (1, 26) | 0 (0, 24) | 77 (58, 89) |
| Day 85 | n | 16 | 18 | 12 | 26 |
| | GMT (95% CI) | 2934·4 (1584·6, 5434·2) | 3879·4 (2584·3, 5823·6) | 1198·6 (559·5, 2567·8) | 4902·3 (3198·6, 7513·5) |
| | GMFR[a] (95% CI) | 1·2 (0·9, 1·7) | 0·9 (0·8, 1·1) | 0·7 (0·6, 0·9) | 4·5 (3·0, 6·7) |
| | 4-Fold Rise[b] (95% CI) | 6 (1, 28) | 0 (0, 18) | 0 (0, 24) | 58 (39, 74) |
| Day 113 | n | 14 | 18 | 12 | 26 |
| | GMT (95% CI) | 2049·4 (1005·0, 4179·3) | 3456·2 (2339·7, 5105·4) | 1269·9 (556·1, 2899·8) | 3200·0 (2188·9, 4678·2) |
| | GMFR[a] (95% CI) | 0·8 (0·6, 1·1) | 0·8 (0·7, 1·0) | 0·7 (0·5, 1·1) | 2·9 (2·2, 3·8) |
| | 4-Fold Rise[b] (95% CI) | 0 (0, 22) | 0 (0, 18) | 0 (0, 24) | 54 (35, 71) |
| Maximum Titer Post-Challenge | n | 16 | 18 | 13 | 26 |
| | GMT (95% CI) | 4525·5 (2245·5, 9120·5) | 4887·8 (3237·6, 7379·2) | 1600·0 (717·4, 3568·2) | 6400·0 (4273·9, 9583·8) |
| Peak-Fold Rise | n | 16 | 18 | 13 | 26 |
| | GMFR[a] (95% CI) | 1·9 (1·3, 2·8) | 1·2 (0·9, 1·5) | 1·0 (0·7, 1·3) | 5·8 (3·7, 9·0) |
| | 4-Fold Rise[bc] (95% CI) | 31 (14, 56) | 6 (1, 26) | 0 (0, 23) | 77 (58, 89) |

N = Number of participants in the Immunogenicity Population who received challenge.

[a] GMFR represents the geometric mean fold rise in IgG antibody compared to pre-challenge (Day 56).

[b] 4-Fold Rise represents the percentage of participants with at least a 4-Fold Rise in IgG antibody compared to pre-challenge (Day 56).

[c] 4-Fold Rise at Peak-Fold Rise represents the percentage of participants with at least a 4-Fold Rise in IgG antibody at the maximum titer value post-dose compared to pre-challenge (Day 56).

CFU= colony-forming units

**Table S12: Post-Challenge Invaplex-specific IgA GMT, GMFR, and Seroresponse (≥ 4-Fold Rise) Results with 95% Confidence Intervals by Time Point and Study Arm, Immunogenicity Population**

| Time Point | Statistic | 2-dose $10^6$ cfu (N=16) | 2-dose $5x10^5$ cfu (N=18) | 1-dose $10^6$ cfu (N=13) | Placebo (N=26) |
|---|---|---|---|---|---|
| Day 64 | n | 16 | 18 | 13 | 26 |
| | GMT (95% CI) | 322·1 (130·7, 793·7) | 207·9 (125·0, 345·5) | 290·5 (209·9, 402·1) | 282·8 (166·0, 481·8) |
| | GMFR[a] (95% CI) | 1·4 (0·9, 2·1) | 0·9 (0·8, 1·1) | 0·7 (0·5, 1·1) | 3·3 (2·0, 5·5) |
| | 4-Fold Rise[b] (95% CI) | 19 (7, 43) | 0 (0, 18) | 0 (0, 23) | 50 (32, 68) |
| Day 71 | n | 15 | 18 | 12 | 26 |
| | GMT (95% CI) | 364·7 (133·7, 995·1) | 466·6 (237·7, 916·1) | 299·7 (201·6, 445·5) | 1477·0 (842·2, 2590·3) |
| | GMFR[a] (95% CI) | 1·6 (0·8, 3·3) | 2·1 (1·0, 4·2) | 0·7 (0·4, 1·3) | 17·3 (9·7, 30·9) |
| | 4-Fold Rise[b] (95% CI) | 33 (15, 58) | 44 (25, 66) | 8 (1, 35) | 85 (66, 94) |
| Day 85 | n | 16 | 18 | 12 | 26 |
| | GMT (95% CI) | 270·9 (120·6, 608·2) | 224·5 (133·6, 377·1) | 168·2 (127·9, 221·1) | 482·1 (300·6, 773·0) |
| | GMFR[a] (95% CI) | 1·1 (0·7, 2·0) | 1·0 (0·6, 1·6) | 0·5 (0·3, 0·8) | 5·7 (3·6, 8·9) |
| | 4-Fold Rise[b] (95% CI) | 19 (7, 43) | 17 (6, 39) | 0 (0, 24) | 85 (66, 94) |
| Day 113 | n | 14 | 18 | 12 | 26 |
| | GMT (95% CI) | 190·3 (107·0, 338·6) | 147·0 (91·3, 236·6) | 126·0 (75·8, 209·5) | 189·6 (122·1, 294·5) |
| | GMFR[a] (95% CI) | 0·7 (0·5, 1·0) | 0·7 (0·5, 0·9) | 0·3 (0·2, 0·5) | 2·2 (1·6, 3·2) |
| | 4-Fold Rise[b] (95% CI) | 0 (0, 22) | 0 (0, 18) | 0 (0, 24) | 35 (19, 54) |
| Maximum Titer Post-Challenge | n | 16 | 18 | 13 | 26 |
| | GMT (95% CI) | 590·7 (219·3, 1591·0) | 565·7 (301·8, 1060·4) | 323·2 (225·9, 462·3) | 1557·9 (929·8, 2610·3) |
| Peak-Fold Rise | n | 16 | 18 | 13 | 26 |
| | GMFR[a] (95% CI) | 2·5 (1·4, 4·5) | 2·5 (1·4, 4·5) | 0·8 (0·5, 1·3) | 18·3 (10·6, 31·5) |
| | 4-Fold Rise[bc] (95% CI) | 44 (23, 67) | 44 (25, 66) | 8 (1, 33) | 88 (71, 96) |

N = Number of participants in the Immunogenicity Population who received challenge.
[a] GMFR represents the geometric mean fold rise in IgA antibody compared to pre-challenge (Day 56).
[b] 4-Fold Rise represents the percentage of participants with at least a 4-Fold Rise in IgA antibody compared to pre-challenge (Day 56).
[c] 4-Fold Rise at Peak-Fold Rise represents the percentage of participants with at least a 4-Fold Rise in IgA antibody at the maximum titer value post-dose compared to pre-challenge (Day 56).
CFU= colony-forming units

**Table S13: Summary of the Duration (Days) of *S. sonnei* Shedding Pre-Challenge by Study Arm for the Shedding Analysis Population**

| Evaluation | Statistic | 2-dose $10^6$ or $5x10^5$ CFU (N=41) | 2-dos $10^6$ CFU (N=18) | 2-dose $5x10^5$ CFU (N=23) | 1-dose $10^6$ CFU (N=16) | Placebo (N=30) |
|---|---|---|---|---|---|---|
| Culture | n | 36 | 14 | 22 | 11 | 1 |
| | Mean | 18·2 | 21·9 | 15·9 | 13·3 | 1·0 |
| | Standard Deviation | 15·0 | 14·6 | 15·0 | 10·3 | - |
| | Median | 14·5 | 29·0 | 11·0 | 11·0 | 1·0 |
| | IQR | 23·0 | 25·0 | 21·0 | 20·0 | - |
| | Min, Max | 1, 49 | 1, 41 | 1, 49 | 1, 26 | 1, 1 |
| Immunoblot | n | 30 | 13 | 17 | 10 | 1 |
| | Mean | 15·8 | 15·2 | 16·4 | 5·8 | 1·0 |
| | Standard Deviation | 14·7 | 16·5 | 13·7 | 5·7 | - |
| | Median | 12·0 | 6·0 | 15·0 | 2·5 | 1·0 |
| | IQR | 28·0 | 28·0 | 18·0 | 11·0 | - |
| | Min, Max | 1, 42 | 1, 40 | 1, 42 | 1, 13 | 1, 1 |

N = Number of participants in the Shedding Analysis Population who received both vaccinations corresponding to the study arm.
n = Restricted to participants with Shigella detected by the given assay any time post-vaccination but prior to challenge period (Days 1 to 56).
CFU= colony-forming units; IQR=Interquartile Range; Min, Max=Minimum and Maximum values.

**Table S14: Summary of the Duration (Days) of *S. sonnei* Shedding Post-Challenge by Culture and Immunoblot by Study Arm for the Full Analysis Population**

| Evaluation | Statistic | 2-dose $10^6$ or $5x10^5$ CFU (N=34) | 2-dose $10^6$ CFU (N=16) | 2-dose $5x10^5$ CFU (N=18) | 1-dose $10^6$ CFU (N=13) | Placebo (N=26) |
|---|---|---|---|---|---|---|
| Culture | n | 18 | 10 | 8 | 8 | 23 |
| | Mean | 2·6 | 2·6 | 2·6 | 2·5 | 3·1 |
| | Standard Deviation | 1·2 | 1·3 | 1·2 | 1·3 | 1·3 |
| | Median | 3·0 | 3·0 | 2·5 | 2·5 | 4·0 |
| | IQR | 1·0 | 3·0 | 1·0 | 1·5 | 2·0 |
| | Min, Max | 1, 5 | 1, 4 | 1, 5 | 1, 5 | 1, 5 |
| Immunoblot | n | 18 | 9 | 9 | 5 | 23 |
| | Mean | 2·7 | 2·3 | 3·1 | 1·6 | 3·0 |
| | Standard Deviation | 1·4 | 1·5 | 1·2 | 1·3 | 1·2 |
| | Median | 3·0 | 2·0 | 3·0 | 1·0 | 3·0 |
| | IQR | 1·0 | 2·0 | 1·0 | 0·0 | 2·0 |
| | Min, Max | 1, 5 | 1, 5 | 2, 5 | 1, 4 | 1, 5 |

N = Number of participants in the Shedding Analysis Population who received challenge.
n = Restricted to participants with Shigella detected by the given assay any time post-challenge.
CFU= colony-forming units; IQR=Interquartile Range; Min, Max=Minimum and Maximum values.

**Table S15: Summary of Maximum *S. sonnei* Colony Forming Units per Gram of Stool by Immunoblot Post-Challenge by Study Arm for the Full Analysis Population.**

| Evaluation | Parameter | Statistic | 2-dose ($10^6$ or $5x10^5$ CFU) (N=34) | 2-dose ($10^6$ CFU) (N=16) | 2-dose ($5x10^5$ CFU) (N=18) | 1-dose ($10^6$ CFU) (N=13) | Placebo (N=26) |
|---|---|---|---|---|---|---|---|
| Immunoblot | Peak Concentration (CFU/g) | n | 18 | 9 | 9 | 5 | 23 |
| | | Geometric Mean | 410165·7 | 370397·7 | 454203·4 | 63135·8 | 4546451·9 |
| | | 95% CI | 121149·0, 1388669·8 | 33883·8, 4048966·3 | 117971·8, 1748729·4 | 1973·1, 2020278·3 | 2179543·5, 9483740·4 |
| | | Min, Max | 11400, 71400000 | 11400, 71400000 | 16600, 4000000 | 1140, 1430000 | 74000, 70000000 |
| | Day of Peak Concentration | n | 18 | 9 | 9 | 5 | 23 |
| | | Mean (SD) | 61.0 (1·6) | 61.3 (0·7) | 60.7 (2·1) | 61·6 (0.9) | 60.4 (1·1) |
| | | Median (IQR) | 61.5 (1·0) | 61.0 (1·0) | 62.0 (1·0) | 62.0 (0) | 60.0 (1·0) |
| | | Min, Max | 57, 62 | 60, 62 | 57, 62 | 60, 62 | 59, 62 |

N = Number of participants in the Full Analysis Population. n = Restricted to participants with Shigella detected by the given assay any time post-challenge but prior to discharge (Days 57 to 65). Concentrations below the LLOQ but above the LOD were set to ½*LLOQ. Concentrations above the ULOQ were set to the ULOQ. CFU=colony-forming units; g=gram; SD =Standard Deviation; IQR= Interquartile Range; CI= Confidence Interval; Min, Max – Minimum, Maximum

## Data Sharing Statement

Data describing the trial is shared in the ClinicalTrials.gov database at the US National Library of Medicine using the identifier NCT 04242264. De-identified individual-participant data and a data dictionary defining the dataset will be made available after publication of the primary outcomes of this trial. The study protocol, statistical analysis plan, and informed consent form will also be available. Data and supportive documents will be made available on request to the NIAID Office of Data Science and Emerging Technologies: datascience@niaid.nih.gov. A data sharing agreement must be signed before data are shared.
