# Supplementary material for: Efficacy of WRSs2, a live-attenuated Shigella sonnei vaccine, against shigellosis in a controlled human infection model in the USA: a phase 2, double-blind, randomised, placebo-controlled trial
Source: Lancet Infect Dis. Author manuscript; Available in PMC 2026 Aug 4. (PMC13435326; doi:10.1016/S1473-3099(26)00224-0)
Supplement: MMC1 [file NIHMS2193422-supplement-MMC1.pdf]

THE LANCET
Infectious Diseases

Supplementary appendix 1

This appendix formed part of the original submission and has been peer reviewed. We post it as supplied by the authors.

Supplement to: Rouphael N, Baqar S, Dickey M, et al. Efficacy of WRSs2, a live-attenuated *Shigella sonnei* vaccine, against shigellosis in a controlled human infection model in the USA: a phase 2, double-blind, randomised, placebo-controlled trial. *Lancet Infect Dis* 2026; published online June 30. https://doi.org/10.1016/S1473-3099(26)00224-0.

A Double-Blind Placebo Controlled, Randomized Phase 2 Trial to Evaluate the Safety, Reactogenicity and Immunogenicity of a Live-Attenuated *Shigella sonnei* Vaccine, WRSs2 and Determine its Efficacy in a Challenge Model of *S. sonnei* 53G in Healthy Adults

DMID Protocol Number: 17-0112

DMID Funding Mechanism: HHSN272201300016I/HHSN27200014
FY.2018.A1B1C1D1.0077

Pharmaceutical Support Provided by: Department of Defense

IND Sponsor: NIAID/DMID

Lead Principal Investigator: Robert W. Frenck, Jr, M.D.

DMID Clinical Project Manager: Krista Cato, MHA, BSN, RN

Draft or Version Number: 8.0

14Sept2023

STATEMENT OF ASSURANCE

Each Institution will hold a current Federal Wide Assurance (FWA) issued by the Office of Human Research Protections (OHRP) for federally-funded human subjects research. Each FWA will designate at least one Institutional Review Board (IRB)/Independent Ethics Committee (IEC) registered with OHRP, for which the research will be reviewed and approved by the IRB/IEC and will be subject to continuing review [45 CFR 46.103(b)]. The IRB/IEC designated under an FWA may include an institution's IRB/IEC, an independent IRB/IEC, or an IRB/IEC of another institution after establishing a written agreement with that other institution.

STATEMENT OF COMPLIANCE

The trial will be carried out in accordance with Good Clinical Practice (GCP) and as required by the following:

United States Code of Federal Regulations (CFR) 45 CFR Part 46: Protection of Human Subjects

Food and Drug Administration (FDA) Regulations, as applicable: 21 CFR Part 50 (Protection of Human Subjects), 21 CFR Part 54 (Financial Disclosure by Clinical Investigators), 21 CFR Part 56 (Institutional Review Boards), 21 CFR Part 11, and 21 CFR Part 312 (Investigational New Drug Application), 21 CFR 812 (Investigational Device Exemptions)

International Conference on Harmonisation: Good Clinical Practice (ICH E6); 62 Federal Register 25691 (1997); and future revisions

Belmont Report: Ethical Principles and Guidelines for the Protection of Human Subjects of Research, Report of the National Commission for the Protection of Human Subjects of Biomedical and Behavioral Research

National Institutes of Health (NIH) Office of Extramural Research, Research Involving Human Subjects, as applicable

National Institute of Allergy and Infectious Diseases (NIAID) Clinical Terms of Award, as applicable

Applicable Federal, State, and Local Regulations and Guidance

SIGNATURE PAGE

The signature below provides the necessary assurance that this trial will be conducted according to all stipulations of the protocol, including all statements regarding confidentiality, and according to local legal and regulatory requirements and applicable US federal regulations and ICH E6 Good Clinical Practice (GCP) guidelines.

I agree to conduct the study in compliance with GCP and applicable regulatory requirements.

I agree to conduct the study in accordance with the current protocol and will not make changes to the protocol without obtaining the sponsor's approval and IRB/IEC approval, except when necessary to protect the safety, rights, or welfare of subjects.

Site Investigator Signature:

Signed: ____ Date: ________________

Name

Title

TABLE OF CONTENTS

STATEMENT OF ASSURANCE....2
STATEMENT OF COMPLIANCE....3
SIGNATURE PAGE....4
TABLE OF CONTENTS....5
LIST OF TABLES....10
LIST OF FIGURES....11
LIST OF ABBREVIATIONS....12
PROTOCOL SUMMARY....16
1 KEY ROLES....20
2 BACKGROUND AND SCIENTIFIC RATIONALE....21
2.1 Background....21
2.2 Scientific Rationale....25
2.2.1 Scientific Innovation....26
2.2.2 Purpose of Study....26
2.2.3 Study Population....26
2.3 Potential Risks and Benefits....27
2.3.1 Potential Risks....27
2.3.2 Potential Benefits....31
3 STUDY DESIGN, OBJECTIVES AND ENDPOINTS OR OUTCOME MEASURES....31
3.1 Study Design Description....31
3.2 Study Objectives....37
3.2.1 Primary....37
3.2.2 Secondary....**Error! Bookmark not defined.**
3.2.3 Exploratory....37
3.2.4 Secondary Research....37
3.3 Study Endpoints or Outcome Measures....38

3.3.1 Primary ...38
3.3.2 Secondary ...38
3.3.3 Exploratory ...39
3.3.4 Secondary Research...40
4 INTERVENTION/INVESTIGATIONAL PRODUCT...40
4.1 Study Product Description...40
4.1.1 Product Storage and Stability ...42
4.2 Acquisition/Distribution ...43
4.3 Protocol-Specified Medications/Treatments other than Study Products...45
4.4 Dosage/Regimen, Preparation, Dispensing and Administration of Study Intervention/Investigational Product ...45
4.5 Accountability Procedures for the Study Intervention/Investigational Product(s)...47
5 SELECTION OF SUBJECTS AND STUDY ENROLLMENT AND WITHDRAWAL ...48
5.1 Eligibility Criteria...48
5.1.1 Subject Inclusion Criteria ...48
5.1.2 Subject Exclusion Criteria ...49
5.2 Withdrawal from the Study, Discontinuation of Study Product, or Study Termination...51
5.2.1 Withdrawal from the Study or Discontinuation of the Study Product...51
5.2.2 Handling of Withdrawals...52
5.2.3 Study Termination ...52
5.2.4 Study Discontinuation ...53
6 STUDY PROCEDURES...53
6.1 Screening ...53
6.2 Enrollment ...55
6.3 Planned Study Visits...55
6.3.1 Follow-up...68
6.3.2 Final Study Visit...70

6.3.3 Early Termination Visit 70
6.4 Unscheduled Study Visits 71
6.5 Protocol Deviations 71
7 DESCRIPTION OF CLINICAL AND LABORATORY EVALUATIONS 72
7.1 Clinical Evaluations 72
7.1.1 Assessment of Concomitant Medications/Treatments other than Study Product 73
7.1.2 Non-Research Standard of Care 74
7.2 Laboratory Evaluations 74
7.2.1 Clinical Laboratory Evaluations 74
7.2.2 Secondary Research Assays 76
7.2.3 Laboratory Specimen Preparation, Handling, and Storage 76
7.2.4 Laboratory Specimen Shipping 77
8 ASSESSMENT OF SAFETY 77
8.1 Assessing and Recording Safety Parameters 77
8.1.1 Adverse Events (AEs) 78
8.1.2 AE Grading 78
8.1.3 Reactogenicity 80
8.1.4 Serious Adverse Events (SAEs) 80
8.2 Specification of Safety Parameters 81
8.2.1 Solicited Events 81
8.2.2 Unsolicited Events 81
8.3 Reporting Procedures 81
8.3.1 Reporting SAEs 81
8.3.2 Regulatory Reporting for Studies Conducted Under DMID-Sponsored IND 82
8.3.3 Reporting of Pregnancy 83
8.4 Type and Duration of Follow-up of Subjects after AEs 83
8.5 Procedures to be Followed in the Event of Abnormal Laboratory Test Values or Abnormal Clinical Findings 83

8.6 Study Halting Rules 84
8.6.1 Individual Halting Criteria 84
8.7 Safety Oversight (DSMB, as applicable) 84
8.7.1 Data and Safety Monitoring Board (DSMB) 84
9 HUMAN SUBJECTS PROTECTION 86
9.1 Institutional Review Board/Independent Ethics Committee 86
9.2 Informed Consent Process 86
9.3 Consent for Secondary Research of Residual Specimens and Data 88
9.4 Exclusion of Women, Minorities, and Children (Special Populations) 89
9.5 Subject Confidentiality 89
9.6 Certificate of Confidentiality 90
9.7 Costs, Subject Compensation, and Research Related Injuries 91
10 STATISTICAL CONSIDERATIONS 91
10.1 Study Hypotheses 91
10.2 Sample Size Considerations 91
10.3 Treatment Assignment Procedures 92
10.3.1 Randomization Procedures 92
10.3.2 Masking Procedures 93
10.4 Planned Interim Analyses 93
10.4.1 Interim Safety Review 93
10.4.2 Interim Immunogenicity or Efficacy Review 94
10.5 Final Analysis Plan 94
10.5.1 Endpoint Review Committee 94
10.5.2 Analysis Populations 94
10.5.3 Primary Efficacy Analysis 95
10.5.4 Safety Data 95
10.5.5 Immunogenicity Data 96
10.5.6 Missing Values and Outliers 96

11 SOURCE DOCUMENTS AND ACCESS TO SOURCE DATA/DOCUMENTS 97
12 QUALITY CONTROL AND QUALITY ASSURANCE 97
13 DATA HANDLING AND RECORD KEEPING 98
13.1 Data Management Responsibilities 98
13.2 Data Coordinating Center/Biostatistician Responsibilities 98
13.3 Data Capture Methods 99
13.4 Types of Data 99
13.5 Study Records Retention 99
14 CLINICAL MONITORING 99
15 PUBLICATION POLICY 100
16 LITERATURE REFERENCES 101
17 APPENDICES 104
Appendix A: Schedule of Events 105
Appendix B: Toxicity Table/Laboratory AE Grading Scale 113
Appendix C: Vital Signs ADVERSE EVENT Grading Scale 114
Appendix D: Diarrhea Classification 115
Appendix E: Reactogenicity Grading Scale 116

LIST OF TABLES

Table 1: *S. sonnei* strain 53G Controlled Human Infection Clinical Outcome 25
Table 2: Maximum Severity of Solicited Adverse Events in Subjects Receiving WRSs2 28
Table 3: Vaccine Administration, Challenge and Follow-up 32
Table 4: Primary Endpoint...38

LIST OF FIGURES

Figure 1: Schematic of Study Design....................19

LIST OF ABBREVIATIONS

AE	Adverse Event
ALS	Antibody Lymphocyte Supernatant
ALT	Alanine Aminotransferase
ANC	Absolute Neutrophil Count
ASC	Antibody Secreting Cell
AST	Aspartate Aminotransferase
BPM	Beats Per Minute
BUN	Blood Urea Nitrogen
°C	Degrees Celsius
CCHMC	Cincinnati Children's Hospital Medical Center
CMS	Clinical Materials Services
CFR	Code of Federal Regulations
CFU	Colony Forming Units
COVID-19	Coronavirus Disease 2019
CRF	Case Report Form
CVD	Center for Vaccine Development
DCC	Data Coordinating Center
DSMB	Data and Safety Monitoring Board

eCRF	Electronic Case Report Form
ELISA	Enzyme-Linked Immunosorbent Assay
ERC	Endpoint Review Committee
°F	Degrees Fahrenheit
FDA	Food and Drug Administration
FWA	Federal Wide Assurance
GCP	Good Clinical Practice
HEENT	Head, Eyes, Ears, Nose, Throat
Hgb	Hemoglobin
HIV	Human Immunodeficiency Virus
IBD	Inflammatory Bowel Disease
IBS	Irritable Bowel Syndrome
ICF	Informed Consent Form
ICH	International Conference on Harmonisation
IDES	Internet Data Entry System
IEC	Independent or Institutional Ethics Committee
IgA	Immunoglobulin A
IgG	Immunoglobulin G
IND	Investigational New Drug Application

IpaB	Invasion plasmid antigen B
IpaC	Invasion plasmid antigen C
IRB	Institutional Review Board
KI	Killing Index
MAAE	Medically Attended Adverse Event
MOP	Manual of Procedures
N	Number (typically refers to subjects)
NDA	New Drug Application
NPO	Nothing by mouth
OHRP	Office for Human Research Protections
ORS	Oral Rehydration Solution
PBF	Pilot Bioproduction Facility
PBMC	Peripheral Blood Mononuclear Cell
PBS	Phosphate Buffered Saline
PE	Physical Exam
QA	Quality Assurance
QC	Quality Control
PI-IBS	Post-Infectious Irritable Bowel Syndrome
qPCR	quantitative Polymerase Chain Reaction

RPR	Rapid Plasma Reagin
SAE	Serious Adverse Event
SARS-CoV-2	Severe Acute Respiratory Syndrome Coronavirus 2
SBA	Serum Bactericidal Assay
SDCC	Statistical Data Coordinating Center
SOP	Standard Operating Procedure
SOC	System Organ Class
SWI	Sterile Water for Injection, USP
UC	Ulcerative Colitis
ULN	Upper Limit of Normal
WBC	White Blood Cell
WRAIR	Walter Reed Army Institute of Research

PROTOCOL SUMMARY

Title: A Double-Blind Placebo Controlled, Randomized Phase 2 Trial to Evaluate the Safety, Reactogenicity and Immunogenicity of a Live-Attenuated *Shigella sonnei* Vaccine, WRSs2 and Determine its Efficacy in a Challenge Model of *S. sonnei* 53G in Healthy Adults

Design of the Study: This is a double-blind, placebo controlled, randomized study to test the safety, reactogenicity, immunogenicity and efficacy of up to a 10^6 cfu dose of an oral live-attenuated *Shigella sonnei* vaccine (WRSs2) to protect against shigellosis after a targeted oral challenge with *S. sonnei* 53G, a virulent strain of *S sonnei*. Up to 120 subjects will be enrolled and randomized in this study. Using the 10^6 CFU dose, subjects were randomized 1:1:1 into one of three vaccination arms to receive 2 doses of study agent 28 days apart. Arm 1 received 2 doses of WRSs2, Arm 2 received placebo followed by WRSs2 and Arm 3 received 2 doses of placebo. Twenty-eight ($\pm$2) days after the second dose of study agent, subjects were admitted to the inpatient unit and given an oral challenge of approximately 1500 cfu of 53G. The goal was to have 90 subjects (30 per arm) receive a challenge dose of 53G.

After the initiation of the study, two participants had Grade 3 diarrhea and/or vomiting in the days following vaccination. This triggered a halting rule. To meet the recommendations of the DSMB and evaluate the safety and efficacy signal of a lower dose, several changes to the protocol were made. The vaccination dose was reduced to $5x10^5$, enrollment was changed to 2 arms and randomized 2:1 (vaccine: placebo). Furthermore, participants with morbid obesity were excluded and weight loss medications prohibited. No changes in sample collection or challenge phase were made.

Study Phase: Phase 2

Study Population: Up to 120 healthy male and non-pregnant females, 18-49 years inclusive at the time of first vaccination, recruited from the general population at the participating VTEU site(s)

Number of Sites: Up to 3 sites

Description of Study Product or Intervention: Up to a 10^6 cfu oral dose of live attenuated *S. sonnei* vaccine WRSs2, 0.9% sterile normal saline as placebo and targeted oral challenge of approximately $1.5x10^3$ cfu of wild type *S. sonnei* 53G.

For this amendment, the remaining subjects to be randomized will receive $5x10^5$ cfu vaccine dose or placebo.

Study Objectives:

Primary:

Estimate combined vaccine efficacy of 2 doses of WRSs2 (10^6 cfu or $5x10^5$ cfu) in preventing shigellosis, as defined in Table 4, following challenge with *S. sonnei* strain 53G

Secondary:

Estimate vaccine efficacy of 1 dose of 10^6 cfu, 2 doses of 10^6 cfu, and 2 doses of $5x10^5$ cfu of WRSs2 in preventing shigellosis following challenge with *S. sonnei* strain 53G

Safety evaluations of WRSs2

Evaluate immune responses following vaccination (immunogenicity) with WRSs2 and after challenge with *S. sonnei* strain 53G by serum anti-LPS and anti-Invaplex IgG and IgA by ELISA

Determine fecal shedding of *S. sonnei* after WRSs2 vaccination and 53G challenge by qualitative stool culture

Exploratory:

Evaluate fecal shedding of *S. sonnei* post vaccination and post challenge by colony immunoblot and PCR

Evaluate *S. sonnei* antigen (LPS and Invaplex) specific IgG and IgA-ASC following vaccination and challenge

Evaluate fecal IgA following vaccination and challenge

Secondary Research:

Blood and stool samples will be collected and stored to characterize in depth innate and acquired immune response to WRSs2 and *Shigella* infection.

**Duration of Individual Subject Participation:**	Approximately 8 months
**Estimated Time to Last Subject/Last Day:**	Approximately 24 months

Figure 1: Schematic of Study Design

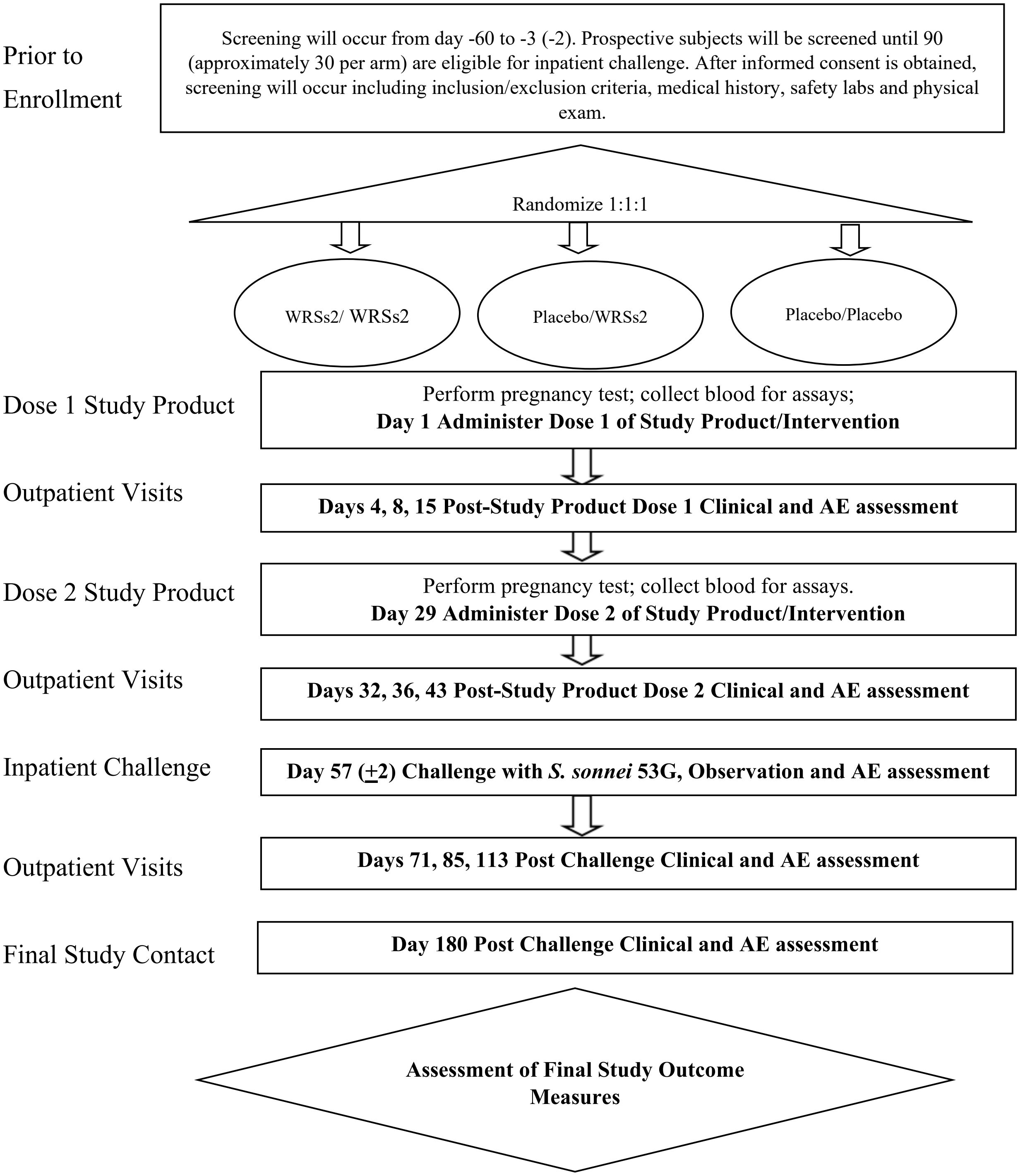

Figure 2: Change in Schematic of Study Design

Following changes will be implemented in the schematic design of the study as described in Figure 1.

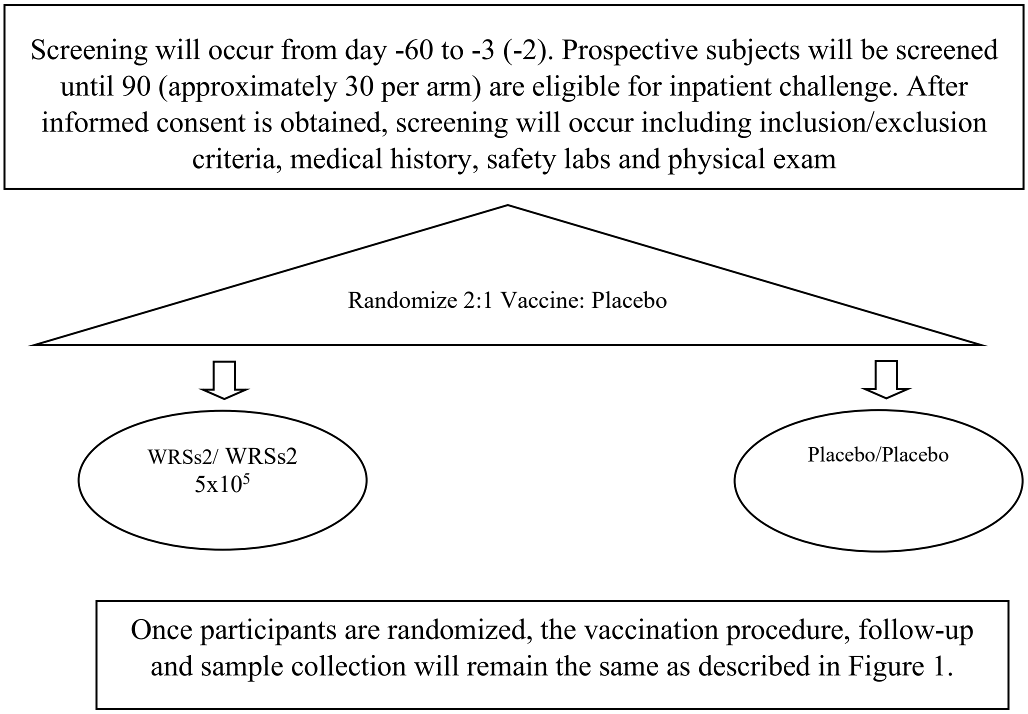

1 KEY ROLES

**Lead Principal Investigator:**	Robert W. Frenck, Jr., MD<br>Director, Gamble Vaccine Research Center<br>Cincinnati Children's Hospital Medical Center<br>3333 Burnet Ave, Cincinnati, OH, 45229<br>Ph: 513-636-4463<br>robert.frenck@cchmc.org
**DMID Clinical Project Manager:**	Krista Cato, MHA, BSN, RN<br>5601 Fishers Lane Room 8F56 Bethesda, MD 20892-9825<br>Ph:240-747-7567<br>Krista.cato@nih.gov
**Statistical and Data Coordinating Center**	The Emmes Company, LLC<br>401 N. Washington St., Suite 700<br>Rockville, MD 20850<br>Phone: 301-251-1161<br>Email: enterics@emmes.com

2 BACKGROUND AND SCIENTIFIC RATIONALE

2.1 Background

Diarrheal diseases continue to be major public health problems throughout the world. According to the 2015 Global Burden of Disease (GBD) study, diarrhea was estimated to be a leading cause of death (1.31 million deaths) among all ages as well as a leading cause of disability adjusted life years (DALYs) (71.59 million DALYs)[1]. After rotavirus, *Shigella* was the second leading cause

of diarrheal deaths (~164,300)[1]. Additionally, diarrhea caused by *Shigella* continues to be a common cause of mortality and morbidity in children <5 years of age in developing countries where malnutrition and unsafe water and sanitation constituted leading risk factors for diarrheal deaths and morbidity[2]. More recent estimates of mortality rates are lower, however epidemiological information on incidence rates of *Shigell*a-mediated diarrhea from much of Asia and Africa is missing[3].

Shigella is comprised of four species (*S. dysenteriae*, *S. flexneri*, *S. boydii* and *S. sonnei)* with *S. sonnei* being the most common species of *Shigella*-associated diarrhea in developed countries and *S. flexneri* being the most common species of *Shigella*-associated diarrhea in the developing world[4].

Shigella is a low-inoculum infection transmitted by the fecal-oral route with as few as 10 colony forming units (cfu) sufficient to cause disease[5]. Due to efficiency of transmission, multiple secondary cases from an initial case are common[5]. Infections caused by *Shigella* can range from mild watery diarrhea to severe disease characterized by fever, headaches, abdominal cramps, and dysentery (frequent small volume stools containing blood and mucus). Although *Shigella*-associated diarrhea typically is self-limiting, infection frequently is debilitating for 5-7 days if untreated[3, 6].

Currently, the mainstays against *Shigella* are prevention and treatment of infections. Improved sanitation and education about improved hygienic practices are the principal means of prevention while treatment typically requires administration of antimicrobial therapy[7, 8]. Improving sanitation is difficult, particularly in resource-limited areas thus decreasing the effectiveness of prevention. Additionally, the emergence of multidrug-resistant *Shigella* strains has complicated the treatment of shigellosis[9-11]. Thus, methods to prevent infection, such as vaccines, are critically needed.

A live-attenuated *S. sonnei* vaccine candidate, WRSS1 was derived from a virulent strain of *S. sonnei* called Moseley. The principal attenuating feature of WRSS1 is the loss of the virulence-plasmid encoded protein VirG (IcsA). *Shigella* lacking VirG (IcsA) have decreased intracellular motility and movement from one cell to another which inhibits bacterial spread within the host tissue. Loss of VirG (IcsA) results in a significantly attenuated bacterial strain[12-16].

WRSS1 underwent Phase 1 trials in the US (inpatient) and in Israeli adults (outpatient). The vaccine was highly immunogenic when orally administered at a dose of 10^3 to 10^4 cfu[16, 17]. However, of the subjects who received a $1x10^4$ cfu dose of WRSS1, 15% to 20% developed diarrhea and fever, albeit mild and transient[16, 17]. The safety and efficacy of WRSS1 also was tested among Thai adults[18]. Thirteen adults were given a 1.6 $x10^4$ cfu dose of WRSS1 and then, 60 days after vaccination, administered a $1.67x10^3$ cfu dose of *S. sonnei* 53G to evaluate efficacy

of WRSS1 in protecting against dysentery and shigellosis. Similar to US and Israeli studies, 4 of the 13 (31%) Thai subjects immunized with WRSS1 developed fever or diarrhea, although symptoms were described as mild and not affecting the daily routine of the subjects[18].

Although WRSS1 has been immunogenic, the vaccine was unacceptably reactogenic at doses higher than 10^4 cfu leading to the development of more attenuated vaccine candidates; WRSs2 and WRSs3. The goal for these VirG (IcsA)-based second generation candidates was to maintain immunogenicity while having a better safety profile. In addition to the loss of the *virG (icsA)* gene, WRSs2 has two additional deletions in the genes *senA* and *senB*, that are present on the virulence plasmid[19, 20]. The gene *senA* (also known as *shet2-1* and *ospD3*) encodes the enterotoxin SenA (ShET2-1 and OspD3), which has been shown to cause fluid accumulation in rabbit ileal loops[21, 22]. The gene *senB* (also known as *shet2-2* and *ospD2*) encodes SenB (or ShET 2-2 and OspD2) a similarly sized protein as SenA with 40% identity with SenA at the amino acid level. A recent study using cell culture assays has indicated that SenB may inhibit epithelial cell death, implicating that one of its functions is to facilitate bacterial colonization[23]. The enterotoxic activity of SenB remains to be demonstrated[19, 24]. WRSs3 includes the added deletion of the virulence plasmid-based *msbB*2 gene. Lack of the *msbB*2 gene product has previously been shown to produce a less toxic lipopolysaccharide (LPS) molecule, which may reduce the proportion of vaccinated subjects with fever[19, 24]. Animal safety, immunogenicity and efficacy studies have shown that WRSs2 and WRSs3 compare favorably with WRSS1.

A Phase 1 trial evaluating the safety and immunogenicity of WRSs2 and WRSs3 was recently completed[25]. Healthy adults 18-45 years of age, assigned to 5 cohorts of 18 subjects each (WRSs2 (n=8), WRSs3 (n=8) or placebo (n=2) were housed in an inpatient facility and administered a single oral dose of study agent 5 minutes after ingestion of oral bicarbonate. Ascending dosages of vaccine (from 10^3 cfu to 10^7 cfu) were evaluated. On day 8, treatment with ciprofloxacin (500 mg BID for 3 days) was initiated and subjects were discharged home 2 days after completing antibiotics. Subjects returned for outpatient visits on day 14, 28 and 56 post-vaccination for monitoring and collection of stool and blood samples.

Both WRSs2 and WRSs3 were generally well-tolerated and safe over the entire dose range. Among the 80 vaccinees, 11 subjects developed diarrhea, 8 of which were mild and did not affect daily activities. At the 10^7 cfu dose, moderate diarrhea occurred in one subject receiving WRSs2 while at the same dose of WRSs3, two subjects had moderate to severe diarrhea (see Risk Section for more detailed description of AEs associated with WRSs2). As predicted, doses of WRSs2 and WRSs3 that were 2 logs higher than what was overly reactogenic for WRSS1 were well-tolerated by subjects. Additionally, the attenuation of both WRSs2 and WRSs3 was demonstrated by the fact that despite close housing for 9 days with subjects who received and

excreted either WRSs2 or WRSs3, none of the 9 placebo subjects shed the vaccine candidate nor did placebo recipients develop any symptoms during the inpatient stay.

Humans are the only natural host for *Shigella* making it more difficult to study the prevention and/or treatment of infections with the organism. While non-human primate models have been developed, inoculums that are multiple logs higher than needed to cause an infection in humans, are required to reproducibly induce infection in monkeys. In a rhesus monkey model, 2 $x10^9$ cfu of *Shigella dysenteriae* 1 were required to reproducibly induce disease[26]. Similarly, a dose of $1x10^{11}$ cfu of *Shigella flexneri* 2a was needed to induce infection in an *Aotus nancymaae* monkey model[27]. The limitations of the currently available animal models reinforce the need for a safe and reproducible human challenge model.

While human challenge studies of *Shigella* have produced important data, many concerns have been raised about these data including; lack of standard inoculum, lack of reproducibility, small sample size of study subjects, varying clinical outcome definitions, and minimal understanding of the immune response to infection[28]. Additionally, the method of preparation of the *Shigella* inoculum from a bacterial suspension of freshly harvested cells from agar plates with colony count estimated from a fixed optical density has been cited as a barrier to standardization and reproducibility[28].

A Phase 1 study recently completed, established a controlled human infection model (CHIM) for *S. sonnei* using a lyophilized, standardized inoculum of strain 53G to address some of the concerns raised about earlier studies[29]. The lot of 53G used in the study (lot #1794) was grown, harvested and lyophilized at the WRAIR Pilot Bioproduction Facility under conditions of cGMP. The study was designed as an ascending dose study with the goal to identify the dose of 53G that would induce shigellosis in approximately 60% of subjects. The study outcome is summarized below in Table 1.

Table 1: *S. sonnei* strain 53G Controlled Human Infection Clinical Outcome

	Dose of 53G (n)				
Symptom (%)	500 CFU (10)	800 CFU (10)	900 CFU (10)	1100 CFU (16)	1760 CFU (10)
Diarrhea (any)	20	60	70	75	70
Severe Diarrhea	0	40	30	44	60
Dysentery	0	60	40	38	50
Fever	0	30	10	19	40
Abd pain/cramps	10	90	70	56	70
Headache	50	70	80	63	70
Severe GI Sxs.	0	20	0	6	20

Overall, of subjects receiving the 1.1-1.7 $x10^3$ cfu of 53G, approximately 63% developed one of the primary endpoints of shedding *Shigella* and either having mod-severe diarrhea and fever or mod-severe diarrhea and dysentery with more than one constitutional symptom of at least moderate severity. Therefore, a targeted approximately 1.5 $x10^3$ cfu dose of *S. sonnei* 53G was determined to be the optimal dose to be used in future CHIM studies.

Based on the safety and immunological profile of WRSs2 and the availability of a robust challenge model, we originally planned to evaluate the safety, immunogenicity and protective efficacy of 1 and 2 doses of WRSs2 (10^6 cfu) in a human challenge model in protecting subjects against shigellosis (see Table 4 for definitions) after challenge with a targeted approximately 1.5 $x10^3$ cfu dose of *S. sonnei* 53G (lot# 1794). After the initiation of the study using $1x10^6$ (see version 7.0 for prior study design), two participants had Grade 3 diarrhea and/or vomiting in the days following receipt of study product. This triggered a stopping rule. To meet the recommendations of the DSMB and evaluate the safety and efficacy signal of a lower dose, several changes to the protocol were made. The vaccination dose was reduced to $5x10^5$, enrollment was changed to only two arms and randomized 2:1 (vaccine: placebo). Furthermore, participants with morbid obesity were excluded and weight loss medications prohibited.

2.2 Scientific Rationale

Shigella are exclusively human pathogens. While improved sanitation and education on safe eating practices could lessen the frequency of *Shigella*-associated diarrhea, these methods have been difficult to achieve. Thus, alternative methods to prevent infection, such as vaccines, are

critically needed. As animal models of *Shigella* do not closely mimic infections in humans; to assess the effectiveness of a vaccine against *Shigella*, a human model of infection is required.

2.2.1 Scientific Innovation

Currently, no licensed vaccine exists to prevent infection with *Shigella*. In a dose escalation study, a live attenuated *S. sonnei* vaccine candidate, WRSs2, was shown to be both safe and immunogenic. The next step will be to evaluate the efficacy of WRSs2 using a human challenge infection model (CHIM). In contrast to previous CHIM studies where the challenge strain typically has been obtained by harvesting confluent bacterial lawns from agar plates that were grown from thawed glycerol stocks or starter cultures from one or more earlier passages; in the current project a lyophilized preparation of *S. sonnei* strain 53G (lot# 1794) will be used for the challenge. The use of a lyophilized product provides many advantages over the plating-and-harvesting the bacterial lawn method including a) consistency and reproducibility between the inoculum used for challenge, b) no need to plate-harvest the inoculum for challenge, and c) minimizing the error related to handling viscous and incompletely homogenized bacterial suspensions.

S. sonnei vaccine candidate WRSs2 (lot# 1501) and challenge strain 53G (lot# 1794) have been successfully produced and lyophilized by WRAIR and demonstrated highly reproducible colony counts in a recently completed series of *S. sonnei* vaccine trials at Cincinnati Children's Hospital Medical Center (CCHMC)[25, 29].

Another critical area to explore is the "systems biology" to an infection with *Shigella*. In contrast to previous "reductionist" methods of study, systems biology is a field of study that focuses on complex interactions within biological systems, using a holistic approach. By studying the "signature of infections", it may be possible to predict people immune to a specific microorganism as well as who may be at increased risk for developing severe infection. This approach may allow us to better understand the immune response to natural infections which could result in vaccines targeted to stimulate those portions of the immune system.

2.2.2 Purpose of Study

The study is designed to evaluate the safety, reactogenicity, immunogenicity, and efficacy of WRSs2, a live oral *S. sonnei* vaccine candidate to protect against shigellosis. Efficacy will be determined by challenging WRSs2-vaccinated and control subjects with a dose of *S. sonnei* 53G.

2.2.3 Study Population

Healthy males and non-pregnant females, 18-49 years inclusive at the time of first vaccination, regardless of religion, sex, or ethnic background who meet all of the inclusion and none of the

exclusion criteria. Subjects will be recruited from the general population of the participating VTEU sites.

2.3 Potential Risks and Benefits

2.3.1 Potential Risks

Blood Collection/Intravenous Catheter Placement

Drawing blood may cause transient discomfort and may cause people to feel faint. Bruising at the blood draw site may occur but can be prevented or lessened by applying pressure for several minutes. Infection at the site of blood collection is a rare but possible event. If required for intravenous fluid hydration, placement of an intravenous catheter may be associated with mild pain, redness and bruising which may take up to 14 days to resolve. Rarely, infection or a blood clot may develop. Risks of blood collection and intravenous catheter placement will be minimized by using sterile technique and having experienced personnel perform the blood collection.

Study Agent (Live, Attenuated WRSs2 Oral *S. sonnei* Vaccine)

WRSs2 is an attenuated strain, no cases of shigellosis are expected from the vaccine when given at the planned dose of up to 10^6 cfu. Nausea, vomiting, abdominal pain/cramping, headache, anorexia/loss of appetite, chills, muscle and/or joint aches and malaise may occur.

As WRSs2 is derived from a virulent *S. sonnei* strain Moseley, subjects potentially could be at risk for developing the signs and symptoms associated with a *Shigella* infection. However, our experience with the vaccine has demonstrated that, at the dose proposed for the current study, AEs were generally uncommon, of mild severity and resolved spontaneously without treatment.

In a recently completed Phase 1 study, forty subjects (8 subjects per dose) received a single dose of WRSs2 ranging from 10^3 – 10^7 cfu[25]. The vaccine was generally safe and well-tolerated. No subject developed shigellosis, dysentery or dehydration and there were no vaccine-related SAEs. Except for the symptom of moderate diarrhea at the highest vaccine dose of 10^7 cfu, vaccine-associated AEs at lower doses were mild, short-lived and resolved without treatment. Of the six subjects who developed diarrhea after the doses of WRSs2, 5 were mild, characterized by 2-3 loose stools of small volume (average weight 108 g) over one 24-hour period that did not affect the daily routine (Table 2). At the highest WRSs2 dose, one subject had 4 loose stools over a 24-hour period with a total weight of 178 grams with one of the stools being 0.2 grams in weight. On the same day, this subject also experienced a single episode of mild fever (38.4°C) for which he was given a single 200 mg dose of ibuprofen. The fever resolved within 5 hours and never recurred.

Table 2: Maximum Severity of Solicited Adverse Events in Subjects Receiving WRSs2

WRSs2 Dose (n)	Diarrhea		Fever	Headache	Cramps	Vomiting	Myalgia	Arthralgia
	Mild	Mod-Severe	Any	Any	Any	Any	Any	Any
10^3 (8)	1	0	0	1	1	0	1	0
10^4 (8)	1	0	0	3	2	1	0	1
10^5 (8)	0	0	0	4	2	0	0	0
10^6 (8)	1	0	0	1	1	0	0	0
10^7 (8)	2	1	1 (38.4°C)	4	2	0	1	0
Placebo (9)	0	0	0	2	1	0	1	1

S. sonnei 53G

S. sonnei strain 53G is a virulent strain that causes illness ranging from mild (watery diarrhea being the main symptom) to severe (fever and abdominal cramping with frequent small volume blood streaked stools containing mucus). Complaints may include nausea, vomiting, headache, anorexia, muscle and/or joint aches and malaise. Symptoms typically are self-resolving in 5-7 days. However, administration of an effective antibiotic (as will occur in the current study) will significantly reduce, or eliminate, symptoms within 24 hours of initiation. Subjects will be closely monitored, and anyone determined by a study physician to meet criteria for severe illness will be offered antibiotic treatment at that time rather than the scheduled day 5.

Shigella is not commonly associated with significant dehydration as stool volume typically is not large. The *S. sonnei* 53G challenge strain is known to be susceptible to multiple antimicrobial agents including TMP-SMX and quinolones. There is a risk of bacteremia, although *Shigella* bacteremia rarely occurs in adult populations with wild-type *Shigella*[3]. In a study we recently conducted to determine the dose of 53G that would produce illness in approximately 60% of recipients, 56 subjects were given an oral dose of 53G ranging from $0.5x10^3$ cfu to $1.76x10^3$ cfu[29]. Symptoms experienced by subjects were typical of *Shigella* and are listed above in Table 1.

Other *Shigella*-related AEs

Post-Infectious Irritable Bowel Syndrome (PI-IBS)

PI-IBS, a functional bowel disorder characterized by unexplained abdominal discomfort or pain associated with changes in normal bowel patterns, has been described to occur 6-7 times more frequently after an acute enteric infection compared to similar matched controls without such a history[30, 31]. The risk of PI-IBS associated with an attenuated *Shigella* vaccine is unknown. To minimize study risks, subjects with a history of abnormal bowel patterns who might be at higher risk for this post-infectious sequelae will be excluded.

Post-Infectious Reactive Arthritis/Arthropathy

A post-dysenteric reactive arthritis, with ocular and/or urethral inflammation, which has an onset typically 1 to 3 weeks after the onset of diarrhea, has been reported to occur in patients with shigellosis. Data suggest that the risk of reactive arthritis following shigellosis may be higher in persons carrying the human leukocyte antigen (HLA)-B27, thus the exclusion of subjects with a positive HLA-B27 test at screening.

Secondary Spread of Vaccine

Our experience, and that of others, have shown that secondary spread of the vaccine strain is highly unlikely. As part of the WRSs2 study discussed above; 40 subjects received WRSs2, 40 received WRSs3 and 9 received placebo. Subjects were housed in an inpatient unit with antibiotics prescribed to everyone on Day 8 post vaccination. Of the WRSs2 vaccinees, 28 (70%) shed the vaccine while 25 (63%) of the subjects who received WRSs3 shed the strain. Subjects typically shed the vaccine strain in their stool for 2-7 days although some subjects shed until the initiation of antibiotic treatment. In contrast, despite the daily close contact, no placebo subject shed *Shigella* in their stool, strongly suggesting that the risk of secondary transmission is low.

Similarly, a previous live oral *S. sonnei* vaccine candidate, WRSS1, that was derived from the same parental strain Moseley as WRSs2 was evaluated in an inpatient and multiple outpatient Phase 1 trials to further support the contention that secondary spread of the vaccine strain is extremely rare. WRSS1 initially was tested in clinic-based trials at the University of Maryland Center for Vaccine Development[16]. Subsequently, a community-based, outpatient trial of WRSS1, under FDA oversight, was conducted in Tel Aviv, Israel[17]. Three groups of 15 subjects received a dose of WRSS1 ranging from 10^3 to 10^5 cfu in a dose-escalating manner. For each vaccinee, a person who did not receive the vaccine but shared the same living space and toilet facilities was enrolled. Both vaccinees and housemates were asked to collect a daily rectal swab for up to 28 days that was immediately placed in Cary-Blair transport media and stored in a cool

place until delivered to the clinic for culture. While most vaccine recipients shed WRSS1 for approximately 5-6 days, WRSS1 was not isolated from any household contact.

The combination of data from these various *S. sonnei* live-attenuated vaccine trials strongly indicate that secondary spread of the vaccine strain is extremely unlikely and thus the vaccine is safe to administer in the outpatient setting.

Acquisition/Spread of COVID-19

To minimize the risk of subjects acquiring and/or spreading COVID-19, all subjects will be encouraged to be vaccinated against SARS-CoV-2. Additionally, all subjects must have a negative test for SARS-CoV-2 at the time of admission to the inpatient unit. SARS-CoV-2 testing will be repeated at any time a subject exhibits symptoms of COVID-19 illness during the inpatient stay. Subjects with symptoms of COVID-19 will be quarantined to their room until results of testing are available. Anyone testing positive for COVID-19 will remain quarantined in their room until it is medically safe for the subject to be escorted off the inpatient unit. Current COVID-19 guidelines for the study site will be followed.

Other risks

The inclusion and exclusion criteria have been constructed as to select for a population of healthy adults which should minimize the risks to study subjects. However, unrecognized risks still may be associated with administration of the vaccine. Because the risks of the vaccine to a pregnant or nursing woman or fetus are unknown, pregnancy is an exclusion and women of childbearing potential will be counseled against becoming pregnant during their participation in the study.

Rectal swabs

In the event a subject is unable to produce a bulk stool, rectal swabs will be collected. Rectal swabs may cause discomfort for patients and in case of special conditions (e.g., hemorrhoids, and rectal polyps) bleeding might happen. Rectal swabs are only collected when it is not possible to obtain a stool sample that will be needed to document presence/absence of the vaccine and/or challenge strain in the stool. Study staff will instruct the subjects on how to self-collect the rectal swab sample.

Bicarbonate administration

Mild gastrointestinal symptoms may occur due to ingestion of the bicarbonate solution.

Risk related to antibacterial therapy

Ciprofloxacin is the desired antibiotic given to all subjects on the 5th day after challenge and is generally well-tolerated. The most frequently reported drug related events for ciprofloxacin therapy have been nausea, diarrhea, abnormal liver function tests, vomiting, and rash which are all mild to moderate in most cases. Fluoroquinolones, including ciprofloxacin, are associated with an increased risk of tendonitis and tendon rupture in all ages, and in high-risk patients can increase risk of aortic tear or rupture, and QT prolongation. Other symptoms rarely seen include hypersensitivity, dizziness, pseudomembranous colitis, and peripheral neuropathy.

In the case of a subject being allergic to ciprofloxacin, trimethoprim-sulfamethoxazole (Bactrim) will be the alternate antibiotic administered. The most common adverse effects of Bactrim are gastrointestinal disturbances (nausea, vomiting, and anorexia) and allergic skin reactions (such as rash and urticaria).

Spread of the *S. sonnei* 53G Challenge Strain

To minimize the likelihood of transmission of bacteria from a subject to a study staff, "Contact Precautions" (good hand hygiene along with wearing gowns and gloves for activities involving contact with subjects) will be observed by all study personnel. If the staff are working with a subject who is actively vomiting, face masks will be worn.

Subjects will be advised to wash their hands frequently, especially after toilet use and before meals. Subjects will receive counseling and education about gastroenteritis and the necessary precautions to minimize the potential for spread of disease to others prior to discharge.

2.3.2 Potential Benefits

There are no direct medical benefits to the subject as a result of study participation.

The subject, by participating in this study will help provide data about the tolerability, immunogenicity, and efficacy of a candidate vaccine against *S. sonnei* infection. These data will be instrumental in further development of a *Shigella* vaccine for licensure.

3 STUDY DESIGN, OBJECTIVES AND ENDPOINTS OR OUTCOME MEASURES

3.1 Study Design Description

This is a double-blind, placebo controlled, randomized trial to evaluate the safety, reactogenicity, immunogenicity and efficacy of up to a 10^6 cfu dose of an oral live-attenuated oral *S. sonnei*

vaccine candidate, WRSs2, in males and non-pregnant females aged 18-49, inclusive at the time of the first vaccination. This is a two-phase study, an outpatient WRSs2 vaccination phase and an inpatient *S. sonnei* 53G challenge phase (Table 3).

Table 3A: Initial Vaccine Administration, Challenge and Follow-up

Initial Vaccine Administration						
Arm	Outpatient (Vaccination Phase)			Inpatient (CCHMC) (Challenge Phase)		Outpatient
	Study Day		Day 4-57<br>7 visits	Day 57<br>(±2)	Day 58-65 (planned discharge on study day 65)	Day 71-180<br>3 visits, final contact
	1	29 +2d				
1	WRSs2	WRSs2	Safety, vaccine fecal shedding, blood, and stool collection for immunogenicity	challenge with *S. sonnei* 53G	Safety, challenge, fecal shedding, blood, and stool collection for immune responses post challenge	Blood and stool collection for immune responses post challenge<br>Final contact for safety
2	Placebo	WRSs2				
3	Placebo	Placebo				

Up to 120 subjects will be enrolled and randomized 1:1:1 into 3 vaccination arms. Arm 1 received 2 doses of WRSs2, Arm 2 received placebo followed by WRSs2, and Arm 3 received two doses of placebo. Twenty-eight (±2) days after the second dose of study agent, subjects will be admitted to the inpatient unit and given an oral challenge of approximately 1500 cfu of 53G. The goal will be to have 90 subjects receive a challenge dose of 53G.

For the amendment, the remainder of subjects will be randomized into 2 arms at 2:1 (vaccine: placebo) to receive two doses of $5x10^5$ vaccine or placebo. Neither the vaccination regimen nor the 53G challenge dose are being amended. The table below outlines the updated study design.

Table 4B: Amended Vaccine Administration, Challenge and Follow-up

Amended Vaccine Administration						
Arm	Outpatient (Vaccination Phase)			Inpatient (CCHMC) (Challenge Phase)		Outpatient
	Study Day		Day 4-57<br>7 visits	Day 57<br>(±2)	Day 58-65 (planned discharge on study day 65)	Day 71-180<br>3 visits, final contact
	1	29 +2d				
1	WRSs2	WRSs2	Safety, vaccine fecal shedding, blood, and stool collection for immunogenicity	challenge with *S. sonnei* 53G	Safety, challenge, fecal shedding, blood, and stool collection for immune responses post challenge	Blood and stool collection for immune responses post challenge<br>Final contact for safety
2	Placebo	Placebo				

Screening Phase (Outpatient)

During the screening period, Day -60 to Day -1 subjects providing informed consent will have their medical history recorded and be screened for general health status, vital signs, and abbreviated physical exam. In addition, subjects will be asked to provide specimens for urine, blood, and stool testing. Subjects unable to produce a stool will be asked to use a swab to collect the sample to be used for detection of *Shigella* in the stool. In addition, serum samples will be collected to determine eligibility based on *Shigella* LPS-specific IgG levels.

Enrollment/Vaccination Phase (Outpatient)

Eligibility for enrollment will be confirmed by reviewing inclusion and exclusion criteria, review of medical history and concomitant medications, vital signs assessment, negative pregnancy test

for females of child-bearing potential within 24 hours prior to dosing, and targeted physical exam to ensure that there are no contraindications for vaccination.

Vaccination Days 1 and 29

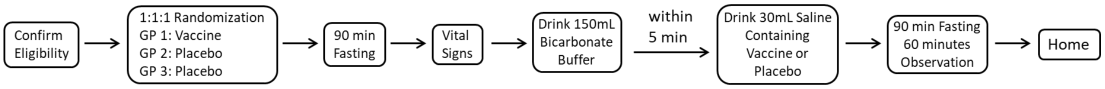

Eligible subjects will be randomized to a vaccination arm per the Internet Data Entry System (IDES) in a 1:1:1 ratio to receive either WRSs2/WRSs2; Placebo/ WRSs2; or Placebo/Placebo.

All participants enrolled after update of protocol V7.0 will be randomized 2:1 in vaccine or placebo groups.

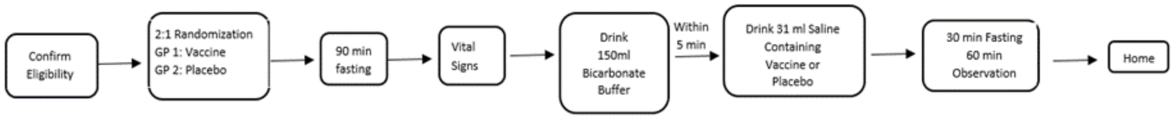

Subjects will be required to be NPO for 90 minutes prior to dosing. Prior to administration of the vaccine or placebo, subjects will drink approximately 150mL of bicarbonate buffer solution to neutralize the stomach acidity. Within 5 minutes, subjects will drink approximately 31 mL of saline containing the vaccine, or placebo (see current MOP for detail instruction of vaccine dilution and administration). Subjects who vomit the bicarbonate buffer solution before drinking the study agent (vaccine or placebo) may be re-dosed with bicarbonate. If a subject vomits the vaccine (WRSs2 or placebo) within 5 minutes of ingestion, the subject may be re-dosed once and allowed to remain in the study.

Subjects will be asked to remain on site and observed for AEs for at least 90 minutes following vaccination to ensure post dose NPO status, have vital signs reassessed at the end of the 90-minute observation period, and to ensure they are stable before they go home.

Subjects will be provided with a thermometer, memory aid and instructions and site contact information. The memory aid will be used to collect all solicited and unsolicited AEs, and medications for the first 7 days after each vaccination starting the evening of administration of the vaccine/placebo. From 8 days until 28 days following each vaccination, only unsolicited

AEs, solicited adverse reactions that continue beyond the 7th day, and related medications will be collected during follow up.

Also, subjects will be instructed on stool collection and storage as well as instructed on the days to return to the clinic with their stool samples.

Through 7 days following each vaccination, if by the evening a subject has not entered data for the day, the subject will be sent automatic email reminders to enter their data.

Subjects will be asked to follow up at the outpatient center post vaccination doses for safety, vaccine fecal shedding, and blood and stool collection for immunogenicity per schedule in Appendix A1 .

Challenge Phase (Inpatient)

Approximately 28 days after the 2nd dose of study agent (28 ± 2 days), subjects will be scheduled for admission to the inpatient unit for about 9 days for challenge with live *S. sonnei* 53G by oral administration. Upon arrival to the inpatient unit, subjects will be assessed as to whether they meet all criteria for the challenge phase. Those who meet criteria for challenge will have SARS-CoV-2 testing performed the result of which must be negative to be eligible to receive the challenge dose of 53G. Any subject positive for SARS-CoV-2 on the test prior to challenge will remain quarantined in room until it is medically safe to be escorted off the inpatient unit and will not receive a challenge dose. However, the subject still would be asked to complete safety follow-ups per protocol.

Biological sample collection will occur per the schedule of events on Day 56 or Day 57 prior to challenge.

Female subjects of child-bearing potential must have a negative pregnancy test within 24 hours prior to the challenge.

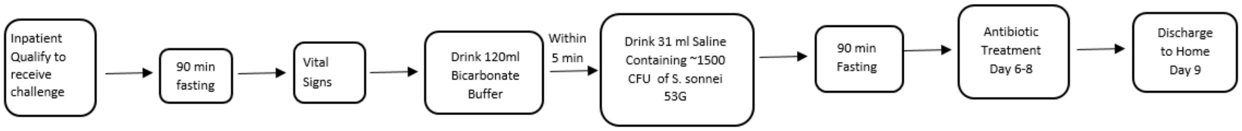

Subjects will be NPO for 90 minutes prior to receiving the *S. sonnei* 53G challenge inoculum. A targeted oral challenge dose of approximately $1.5x10^3$ cfu of *S. sonnei* 53G will be administered per MOP instructions.

Subjects who vomit the bicarbonate buffer solution before drinking the study agent (challenge) may be re-dosed with bicarbonate. A subject who vomits following ingestion of the challenge agent will **not** be re-dosed with the challenge agent. A note will be made in the subject source

documents regarding the apparent amount of the vomitus (small, moderate, or large) along with the time after challenge that the vomiting started. The subject will continue in the study and undergo all study related procedures.

Subjects will be observed for at least 90 minutes following challenge and have vital signs taken at the end of the observation period and be assessed for any AEs.

Post-Challenge (Day 57 to Discharge Day 65(±1)), blood and stool collections will occur, and subjects will be monitored according to the schedule of events. If a subject is unable to produce a stool each day between midnight and 2100, a rectal swab will be obtained to be used for qualitative culturing of *Shigella.* To be eligible for discharge, the subject must have two stool samples that are culture negative for *Shigella* to confirm that the subject is no longer shedding.

On the 5th day after challenge (Day 62), subjects will begin to receive 500 mg of ciprofloxacin twice daily for 3 days. Subjects with contraindication to ciprofloxacin may receive as second-line treatment trimethoprim-sulfamethoxazole (160 mg/800 mg twice daily for 5 days), or any antibiotic that is deemed suitable according to local guidelines and the investigator's assessment can be used.

A subject may be treated with antibiotics **before** the 5th day after challenge if any of the following occur[32]:

1. Subject meets the primary endpoint of shigellosis and continues to feel ill,

   OR

2. In the judgement of the study physician, the subject is too ill to continue without medical intervention.

A subject receiving early antibiotic treatment will remain on the unit until the planned day of discharge. On that day, if a subject has met discharge criteria (clinically well and has had at least 2 consecutive negative stool cultures, collected at least 6 hours apart, for *S. sonnei)*, she/he will be discharged.

A subject that, on the planned day of discharge, still has *S. sonnei* isolated from their stools will be asked to remain in the unit until she/he is no longer shedding. A subject still shedding 48 hours after initiation of ciprofloxacin will be switched to trimethoprim-sulfamethoxazole. If a subject is still positive 48 hours after the second line antibiotic is administered, a culture and antibiotic sensitivity test will be obtained, and therapy initiated based on sensitivity results. If a subject has two consecutive negative stool cultures and meets the criteria to be discharged from the inpatient stay but did not finish the treatment course, she/he can be given the remaining doses of antibiotics to complete at home.

Following discharge, subjects will return at Study Day 71, Study Day 85, and Study Day 113 post challenge for outpatient assessment, occurrence of pregnancy, AE and SAE, and collection of study samples. On Day 180, subjects will complete a final safety contact to assess for occurrence of pregnancy and SAE.

3.2 Study Objectives

3.2.1 Primary

Estimate combined vaccine efficacy of 2 doses of WRSs2 (10^6 cfu or $5x10^5$ cfu) in preventing shigellosis, following challenge with *S. sonnei* strain 53G.

3.2.2 Secondary

Estimate vaccine efficacy of 1 dose of 10^6 cfu, 2 doses of 10^6 cfu, and 2 doses of $5x10^5$ cfu of WRSs2 in preventing shigellosis following challenge with *S. sonnei* 53G

Safety evaluations of WRSs2

Evaluate immune responses following vaccination (immunogenicity) with WRSs2 and after challenge with *S. sonnei* strain 53G by serum anti-LPS and anti-Invaplex IgG and IgA by ELISA

Determine fecal shedding of *S. sonnei* after WRSs2 vaccination and 53G challenge by qualitative stool culture

3.2.3 Exploratory

Evaluate fecal shedding of *S. sonnei* post vaccination and post challenge by colony immunoblot and PCR

Evaluate *S. sonnei* antigen (LPS and Invaplex) specific IgG and IgA-ASC following vaccination and challenge

Evaluate fecal IgA following vaccination and challenge

3.2.4 Secondary Research

Blood and stool samples will be collected and stored to characterize in depth innate and acquired immune response to WRSs2 and *Shigella* infection.

3.3 Study Endpoints or Outcome Measures

3.3.1 Primary

The occurrence of shigellosis (as outlined in Table 4) following challenge with *S. sonnei* strain 53G through Day 63 in the pooled group of subjects receiving two doses of 10^6 cfu or $5x10^5$ cfu of WRSs2 compared to subjects receiving two doses of placebo

Table 5: Primary Endpoint

Shigellosis	Definition
1. Severe Diarrhea	≥6 stools classified as 3-5 in consistency in 24 hours **OR** >800 gm stool classified as 3-5 in 24 hours
2. Moderate Diarrhea with additional signs/symptoms	4-5 stools classified as 3-5 in consistency in 24 hours **OR** 400-800 gm stools classified as 3-5 in 24 hours **with one or more of the following:** oral temperature ≥38.0˚C†; ≥1 moderate constitutional/enteric symptom‡; ≥2 episodes of vomiting in 24 hours
3. Dysentery with additional signs/symptoms	≥2 stools classified as 3-5 in consistency with gross blood (hemoccult positive) in 24 hours **with one or more of the following:** oral temperature ≥38.0˚C; ≥1 moderate constitutional/enteric symptom; ≥2 episodes of vomiting in 24 hours

† Confirmed by two separate readings at least five minutes apart

‡ Moderate constitutional/enteric symptoms include nausea, abdominal pain/cramping, myalgia/arthralgia, malaise (does not include anorexia, chills, headache)

3.3.2 Secondary

The occurrence of shigellosis following challenge with *S. sonnei* strain 53G through Day 63 in subjects receiving 1 dose of 10^6 cfu, 2 doses of 10^6 cfu, or 2 doses of $5x10^5$ cfu of WRSs2 compared to subjects receiving two doses of placebo

Occurrence of solicited systemic AEs through 7 days after each study vaccination

Occurrence of vaccine-related unsolicited AEs through 28 days post last vaccination

Occurrence of SAEs through Study Day 180 or until resolution or stabilization even if this extends beyond the study-reporting period

Number of subjects with ≥ 4-fold rise from pre-vaccination in *Shigella sonnei* LPS-specific and Invaplex-specific serum IgG and IgA by ELISA at Days 15, 29, 43, and 56

Maximum *S. sonnei* LPS-specific and Invaplex-specific serum IgG and IgA titer by ELISA post-vaccination through Day 56

Peak fold-rise in *S. sonnei* LPS-specific and Invaplex-specific serum IgG and IgA titer by ELISA from pre-vaccination through Day 56

Number of subjects with ≥ 4-fold rise from pre-challenge (Day 56) in *S. sonnei* LPS-specific and Invaplex-specific serum IgG and IgA by ELISA at Days 64, 71, 85, and 113

Maximum *S. sonnei* LPS-specific and Invaplex-specific serum IgG and IgA titer by ELISA post-challenge through Day 113

Peak fold-rise in *S. sonnei* LPS-specific and Invaplex-specific serum IgG and IgA titer by ELISA from pre-challenge through Day 113

Number of subjects shedding vaccine strain in their stool by culture pre-vaccination as well as at Days 4, 8, 15, 29, 32, 34, 36 and 43

Number of subjects shedding 53G in their stool by culture post-challenge through Day 65

Duration of shedding *S. sonnei* post-vaccination through Day 56 by culture

Duration of shedding 53G post-challenge through Day 65 by culture

3.3.3 Exploratory

Duration of *S. sonnei* shedding post-vaccination through Day 56 by immunoblot and PCR

Maximum *S. sonnei* CFU per gram of stool post-vaccination through Day 56 by immunoblot

Duration of shedding 53G post-challenge through Day 65 by immunoblot and PCR

Maximum *S. sonnei* CFU per gram of stool post-challenge through Day 65 by immunoblot

Number of *S. sonnei* LPS-specific and Invaplex-specific IgG and IgA ASCs per 10^6 PBMCs by ELISpot pre-vaccination and at Days 8, 29, 36, 56, 60, and 64

Number of subjects with ≥10 IgG or IgA ASCs per 10^6 PBMCs (*S. sonnei* LPS-specific and Invaplex-specific) by ELISpot at any time post-vaccination through Day 56

Number of subjects with ≥10 IgG or IgA ASCs per 10^6 PBMCs (*S. sonnei* LPS-specific and Invaplex-specific) by ELISpot at any time post-challenge through Day 64

Number of subjects with ≥4-fold rise from pre-vaccination in *S. sonnei* LPS-specific and Invaplex-specific fecal IgA and total fecal IgA by ELISA at Day 8, 15, 29, 36, 43, 56, 61, 64, 71, and 85

Maximum fold rise post-vaccination of *S. Sonnei* LPS-specific and Invaplex-specific fecal IgA and total fecal IgA by ELISA through Day 56

3.3.4 Secondary Research

To be determined, endpoints vary based on innate and acquired immunological assays to be used.

4 INTERVENTION/INVESTIGATIONAL PRODUCT

4.1 Study Product Description

WRSs2, *S. sonnei* vaccine candidate, is a live, attenuated vaccine that has been manufactured under cGMP conditions at the WRAIR PBF and it must only be administered to subjects participating in an FDA-approved clinical study according to the inclusion and exclusion criteria specified in the study protocol.

The wild type *S. sonnei* strain was isolated from a laboratory worker "Moseley," accidentally infected in 1975 with a laboratory *S. sonnei* strain obtained from an infected monkey. The infected worker showed classic signs and symptoms of shigellosis such as fever, severe intestinal cramps, diarrhea, and dysentery. The isolated strain termed Moseley is stored as lyophilized vials at WRAIR, and this material was used to manufacture (8 June 2000) a Production Cell Bank (PCB) of Moseley Lot #0794, which is stored at -80°C at the WRAIR, PBF.

A vial (#56) of Moseley strain Lot #0794, received on July 2004 was the starting material for the construction of WRSs2. Specific gene deletion was carried out to obtain the research seed of WRSs2[33]. The method of lambda red recombineering was used to sequentially delete the entire open reading frames (ORFs) of *senA*, *senB*, and *virG(icsA)*. Finally, the strain was made tetracycline sensitive (tet-S) by growth on fusaric acid[33]. The loss of these 3 genes is expected to make this strain when given orally, to be safe, immunogenic, and protective against challenge in volunteers. It is also sensitive to tetracycline and other commonly used antibiotics. The strain constructed was named WRSs2[15, 25, 33].

Research seed of WRSs2 was used to manufacture the lyophilized WRSs2 vaccine products for clinical use. Master Cell Bank (MCB) seeds and PCB seeds were manufactured and stored at -80ºC ± 10ºC and in liquid nitrogen as 1 mL glycerol cultures. A single vial of PCB was used to carry out a 30-liter fermentation, and the contents were lyophilized after the harvested cultures

were suspended in a cryopreservative composed of 7.5% Dextran T10, 2% sucrose and 1.5% glycerol in Dulbecco's phosphate buffered saline (Ca^{++}, Mg^{++} free PBS). The product WRSs2 Lot #1501, was lyophilized in 2 mL aliquots (4.8 x 10^8 cfu/mL) and is stored at -80ºC ± 10ºC under continuous temperature monitoring at WRAIR, PBF. All vials were inspected under extra light and magnification and small (size ranging from 30–400 µm) dark particles were occasionally detected in the lyophilized cake. The content of WRSs2 Lot #1501 vials appears as a solid white cake containing total of 9.6x10^8 cfu/vial.

Lyophilized, frozen vials of WRSs2 (Lot #1501), are labeled as:
Shigella sonnei WRSs2
BPR No.: BPR-887-00 Lot No.: 1501
Contents: 2.0 ml ± 5% (Lyoph.) Storage: -80 ± 10°C
Caution: New drug limited by Federal Law to investigational use only.
Date of Mfg.: 04 Aug 2008
Manufactured By: WRAIR, Silver Spring, MD 20910

Placebo (0.9% Sterile Normal Saline, USP)

0.9% sterile normal saline for human use will be used as the placebo for this trial. The USP grade 0.9% sodium chloride (normal saline) is a sterile, nonpyrogenic, isotonic solution; each mL of fluid contains 9 mg of sodium chloride. It contains no bacteriostatic agent, antimicrobial agent, preservatives, or added buffer and is supplied only in single-dose containers. The solution may contain hydrochloric acid and/or sodium hydroxide for pH adjustment (pH 5.3, range 4.5-7.0).

0.9% sterile normal saline will be stored at 20ºC to 25ºC (68°F to 77ºF) [See USP Controlled Room Temperature]. Study product will remain on site stored as indicated until final accountability is completed. Used and unused product vials will remain on site stored as indicated until final accountability is completed. At study completion the site is given direction from DMID regarding disposition of the product.

The study product will be labeled according to manufacturer or regulatory specifications and include the statement "Caution: New Drug – Limited by Federal Law to Investigational Use."

Challenge (*S. sonnei* 53G)

53G is a virulent *S. sonnei* strain that was initially isolated from a child with diarrhea in Tokyo in 1954. The seed was maintained at the Center for Vaccine Development (CVD), University of Maryland. In Sep 1998, a research seed vial was streaked out on a Congo Red (CR) Tryptic Soy Agar (TSA) plate at the CVD and transferred to WRAIR PBF for the purpose of manufacturing a

MCB (lot# 0593) and PCB (lot# 0599) under cGMP. The MCB and PCB are 1 mL glycerol cultures that are stored at -80ºC ±10ºC and in liquid nitrogen at the WRAIR PBF. In 2013, one vial of the PCB (lot# 0599) was plated on Congo Red plates and isolated Form I colonies were used to grow 53G in 30L of media in a fermentor and the contents harvested and lyophilized as 2 mL aliquots in a medium containing 7.5% Dextran T10, 2% sucrose, and 1.5% glycerol in Dulbecco's phosphate buffered saline (PBS). This standardized, lyophilized lot of *S. sonnei* 53G lot # 1794 was manufactured 25 Feb. 2013 under cGMP conditions at the WRAIR PBF and is stored at -80ºC ± 10ºC at the PBF.

The *S. sonnei* 53G Lot #1794 was lyophilized in 2 mL aliquot (2 x 10^9 cfu/mL) and content of the vials appears as a solid white cake containing total of 4.0 x 10^9 cfu/vial.

The study product is labeled according to manufacturer or regulatory specifications and include the statement "Caution: New Drug – Limited by Federal Law to Investigational Use." (See label below).

***Shigella sonnei* 53G**
BPR No.: **BPR-1094-00** Lot No.: **1794**
Contents: 2.0 ml (Lyoph.) Storage: -80 ± 10 °C
Caution: New Drug – Limited by Federal (or United States) law to investigational use.
Date of Mfg.: 25 Feb 13
Manufactured By: WRAIR, Silver Spring, MD 20910

4.1.1 Product Storage and Stability

The temperature of the storage units at study sites must be manually recorded daily (excluding non-business days and holidays, as applicable) and continuously monitored and recorded during the course of this trial per the participating VTEU site standard operating procedures (SOPs), and documentation will be maintained. If the temperature fluctuates outside of the required range, the affected study product(s) must be quarantined at the correct storage temperature and labeled as 'Do Not Use' (until further notice). The participating VTEU site's research pharmacist must alert the site principal investigator and study coordinator, if the temperature fluctuates outside of the required range. In the event the temperature fluctuates outside of the required range, including accidental deep-freezing or disruption of the cold chain, the affected study product(s) must not be administered. The site principal investigator or responsible person should immediately contact the DMID Product Support Team at DMIDProductSupportTeam@niaid.nih.gov for further instructions before any additional study products are administered. Based on the information collected, DMID and/or the manufacturer will determine whether the affected study product(s) can be used. If it cannot be used, the site will receive specific instructions on how to return the affected study product(s) to the DMID CMS or destroy it on site. Additional instructions for quarantine and DMID contact information are provided in the protocol-specific MOP.

WRSs2 Vaccine Stability

The study vaccine, *Shigella sonnei* WRSs2, lot#1501, is stored at -80°C ±10°C. Once reconstituted, vaccine must be kept on ice and used within 2 hours.

Details regarding results of Viability and Stability Testing are provided in the WRSs2 Vaccine Investigator's Brochure.

Challenge strain (*S. sonnei* 53G)

The challenge material, *Shigella sonnei* strain 53G, is stored at -80 °C ±10°C. Once reconstituted, the maximum hold time is 2 hours on ice between the reconstitution and administration of the product to the subjects.
Details regarding results of Viability and Stability Testing are provided in the *Shigella sonnei* strain 53G Investigator's Brochure.

Placebo (0.9% Sterile Normal Saline, USP)

0.9% sterile normal saline will be stored at 20ºC to 25ºC (68°F to 77ºF) [See USP Controlled Room Temperature]. For excursions between 15-30°C (59°F to 86°F), the product can continue to be used (no quarantine) but the site needs to complete the DMID Study Product Support Team Temperature Excursion Reporting Form and submit to PST. Based on USP guidelines, excursions between 15°C and 30°C are allowed and will not be considered deviations. Study product will remain on site stored as indicated until final accountability is completed. Used and unused product vials will remain on site stored as indicated until final accountability is completed. At study completion the site is given direction from DMID regarding disposition of the product. See protocol-specific MOP for further instructions.

4.2 Acquisition/Distribution

Vaccine (WRSs2, lot# 1501)

The product will be shipped on dry ice with a temperature monitor from WRAIR to the DMID Clinical Agents Repository Contract, Fisher BioServices, which will then distribute them to the study site(s). Upon delivery to sites, the vaccine vials will be stored in the Investigational Pharmacy in a -80°C ±10°C freezer that is consistently monitored for temperature.

Placebo (0.9% Sterile Normal Saline, USP)

0.9% sterile normal saline will be stored at 20ºC to 25ºC (68°F to 77ºF) [See USP Controlled Room Temperature]. Study product will remain on site stored as indicated until final accountability is completed. Used and unused product vials will remain on site stored as

indicated until final accountability is completed. At study completion the site is given direction from DMID regarding disposition of the product. See protocol-specific MOP for further instructions.

Challenge strain (*S. sonnei* 53G, Lot# 1794)

The product will be shipped on dry ice with a temperature monitor from WRAIR to the DMID Clinical Agents Repository Contract, Fisher BioServices, which will then distribute them to the study site(s). Upon delivery to sites, the 53G challenge strain vials will be stored at the Investigational Pharmacy in a -80°C ±10°C freezer that is consistently monitored for temperature.

Upon request by DMID, WRSs2 and 53G, will be transferred to the following address:

DMID Clinical Materials Services (CMS) Contract

Fisher BioServices

20439 Seneca Meadows Parkway

Germantown, MD 20876

Phone: 240-477-1350

Fax: 240-477-1360

Email: DMID.CMS@thermofisher.com

0.9% sterile normal saline will be obtained by the DMID Clinical Materials Services (CMS) Contract, Fisher BioServices.

WRSs2, *S. sonnei* 53G and 0.9% sterile normal saline will be provided through the DMID CMS to the participating VTEU sites prior to the start of this trial upon request and with prior approval from DMID. Should the site principal investigator require additional WRSs2, *S. sonnei* 53G and 0.9% sterile normal saline during this trial, further instructions are provided in the protocol-specific MOP.

Once received, the vaccine, placebo (0.9% sterile normal saline), and challenge material will be stored in and dispensed by the Investigational Pharmacy.

Sterile water for injection (SWI), sodium bicarbonate, ciprofloxacin 500 mg tablets, and trimethoprim-sulfamethoxazole will be provided by the study site, stored per manufacturer instructions, and dispensed by the Investigational Pharmacy.

4.3 Protocol-Specified Medications/Treatments other than Study Products

Ciprofloxacin (500 mg tabs) for human use will be purchased from a commercial vendor with packaging, labeling and storage as per manufacturer's instructions. Subjects will receive 500 mg of ciprofloxacin twice daily for 3 days.

Bactrim (trimethoprim (160)-sulfamethoxazole (800)) for human use will be purchased from a commercial vendor with packaging, labeling and storage as per manufacturer's instructions. Trimethoprim-sulfamethoxazole will be dosed as 1 pill twice daily for 5 days, or any antibiotic that is deemed suitable according to local guidelines and the investigator's assessment can be used.

Sterile water for injection (SWI) for human use will be purchased by the study site and will be stored and prepared per manufacturer's instructions.

Sodium bicarbonate for human use will be purchased by the study site and will be stored and prepared per manufacturer's instructions.

4.4 Dosage/Regimen, Preparation, Dispensing and Administration of Study Intervention/Investigational Product

Vaccine WRSs2

Lyophilized, frozen vials of *S. sonnei* WRSs2 (lot# 1501) will be maintained in Investigational Pharmacy and stored at -80°C ±10°C until the day of vaccination. On the day of vaccination, the requisite number of vials will be removed from the freezer and placed on ice and allowed to thaw for 30 minutes. Then 2 mL of sterile water for injection (SWI) will be added to each vial, this reconstitution will result in $4.8x10^8$ cfu/mL. To ensure complete rehydration of the vaccine strain along with homogeneous mixing of each vial's contents, the vials will remain on ice for another 15 minutes with intermittent swirling of the suspension. All vials then will be combined and diluted in sterile normal saline 0.9% to arrive at a concentration of $5x10^5$ cfu/mL. The diluted vaccine will be kept on ice until administration. At the time of reconstitution, an aliquot will be removed for testing by colony count to be able to document the actual dose administered to the subjects. Time of reconstitution will be noted. A second aliquot will be collected and frozen at -80°C ±10°C until testing is performed to confirm that the bacteria administered to the subjects still possessed the original mutations. Detailed vaccine dilution procedure is described in the current version of the MOP.

One mL of saline containing 10^6 cfu as per version 7.0, and for the final cohort, $5x10^5$ cfu of the WRSs2 vaccine, will be added to 30 mL of sterile normal saline (0.9% NaCl) and placed in a

container. 150 mL of a sodium bicarbonate solution (2 grams of sodium bicarbonate, $NaHCO_3$, in 150 mL of SWI) will be placed in a second container. Placebo will consist of 31 mL sterile normal saline.

Subjects will be NPO for 90 minutes prior to and after receiving the vaccine. At the indicated time, the subjects will be gathered together, and doses distributed. Subjects will drink the sodium bicarbonate solution to neutralize gastric acidity and then drink the **vaccine suspension** or placebo within 5 minutes of drinking the sodium bicarbonate. Care must be taken to ensure that a minimum amount of time, **not to exceed 2 hours**, is spent between the reconstitution of the vaccine and the oral administration of the vaccine to the subjects.

Challenge *S. sonnei* 53G

Lyophilized, frozen vials of *S. sonnei* 53G will be maintained in Investigational Pharmacy and stored at -80°C ±10°C until the day of challenge.

On the day of challenge, one vial will be removed from the freezer and placed on ice and allowed to thaw for 30 minutes. Then 2 mL of cold sterile water (2-8°C) for injection (SWI) will be added to the vial. To ensure complete rehydration of the vaccine strain along with homogeneous mixing of the vial's contents, the vial will remain on ice for another 15 minutes with intermittent swirling of the suspension. The content of the vial will then be diluted in cold, sterile normal saline 0.9% to arrive at the desired concentration of approximately $1.5x10^3$ cfu/mL. The diluted challenge strain will be kept on ice until administration. At the time of reconstitution, an aliquot will be removed for testing by colony count to be able to document the actual dose administered to the subjects. Time of reconstitution will be noted. The maximum hold time is 2 hours on ice between the reconstitution and administration of the challenge product to the subjects. See MOP for details.

One mL of prepared challenge will be added to 30 mL of sterile USP 0.9% saline in a plastic drinking cup. The maximum hold time for placebo will be 2 hours.

Subjects will be NPO for 90 minutes prior to receiving approximately 120 mL solution of bicarbonate buffer (2 gm bicarbonate in 120 mL water). Within 5 minutes of the buffer, subjects will drink approximately 31 mL of saline containing approximately $1.5x10^3$ cfu of the *S. sonnei* 53G suspension.

Pre-determined Modification of Study Intervention/Investigational Product for an Individual Subject

If a subject vomits the vaccine (WRSs2 or placebo) within 5 minutes of ingestion, the subject may be re-dosed once and allowed to remain in the study. Subjects who vomit the dose of study agent the second time will not be administered additional doses of study agent, nor will they be replaced, but will be asked to remain in the study to follow for safety. Subjects who vomit after receipt of the challenge dose of 53G will not be re-dosed due to safety concerns for potentially administering higher than intended dose of virulent *Shigella*.

4.5 Accountability Procedures for the Study Intervention/Investigational Product(s)

The Food and Drug Administration (FDA) requires accounting for the disposition of all investigational products. The Investigator is responsible for ensuring that a current record of product disposition is maintained, and product is dispensed only at an official study site by authorized personnel as required by applicable regulations and guidelines. Records of product disposition, as required by federal law, consist of the date received, date administered, quantity administered, and the subject number to whom the drug was administered.

The Investigational Pharmacist will be responsible for maintaining accurate records of the shipment and dispensing of the investigational product. The pharmacy records must be available for inspection by the DMID monitoring contractors and is subject to inspection by a regulatory agency (e.g., FDA) at any time. An assigned Study Monitor will review the pharmacy records. Used and unused of study products will be retained until monitored and released for disposition, as applicable. Final disposition of the unused study products will be determined by DMID and communicated to the participating sites by the DMID Clinical Project Manager.

The WRSs2 (Lot# 1501**)** vaccine vials will be stored in the Investigational Pharmacy in a -80± 10 °C freezer that is under a temperature-monitoring program. All vials of used and unused product will remain on site stored as indicated until final accountability is completed. At study completion the site is given direction from DMID regarding disposition of the product.

Lyophilized, frozen vials of *S. sonnei* 53G (Lot# 1794) will be maintained in Investigational Pharmacy and stored at -80 °C ±10 °C. Used and unused product vials will be stored in the Investigational Pharmacy until final accountability is completed. At study completion the site is given direction from DMID regarding disposition of the product.

0.9% sterile normal saline will be stored at 20ºC to 25ºC (68°F to 77ºF) [See USP Controlled Room Temperature]. Study product will remain on site stored as indicated until final accountability is completed. Used and unused product vials will remain on site stored as indicated until final accountability is completed. At study completion the site is given direction from DMID regarding disposition of the product.

5 SELECTION OF SUBJECTS AND STUDY ENROLLMENT AND WITHDRAWAL

5.1 Eligibility Criteria

Up to 120 individuals, male or non-pregnant female 18 to 49 years, inclusive at the time of the first vaccination, who are in good health by history and screening values and meet all of the inclusion criteria and none of the exclusion criteria, will be enrolled and randomized to a study group using IDES.

Inclusion and exclusion criteria are applied for enrollment of subjects into the study. The target population should reflect the community at large at each of the participating VTEU sites.

Subject inclusion and exclusion criteria must be assessed by a study clinician licensed to make medical diagnoses and listed on the Form FDA 1572 as the site principal investigator or sub-investigator.

No exemptions are granted on subject inclusion or exclusion criteria in DMID-sponsored studies.

Questions about eligibility should be directed toward the DMID Medical Officer.

5.1.1 Subject Inclusion Criteria

1. Provide informed consent prior to initiation of any study procedures.
2. Are able to understand and comply with planned study procedures and be available for all study visits.
3. Is 18-49 years of age inclusive and in sufficiently good health* to be safely enrolled in this study as determined by medical history, medication use, and abbreviated physical exam.

   *Good health is defined by the absence of any exclusionary medical conditions. If the subject has another current, ongoing medical condition, the condition cannot meet any of the following criteria: 1) first diagnosed within 3 months of enrolment; 2) is worsening in terms of clinical outcome in last 6 months; or 3) involves need for medication that may pose a risk to subject's safety or impede assessment of AEs or immunogenicity if they participate in the study. Topical, nasal, and inhaled medications (with the exception of inhaled corticosteroids as outlined in the Subject Exclusion Criteria [see Section 5.1.2]), herbals, vitamins, and supplements are permitted.

4. Oral temperature is less than 100.4°F.
5. Pulse is 50 to 100 beats per minute (bpm), inclusive.
6. Systolic blood pressure is 90 to 140 mmHg, inclusive.
7. Diastolic blood pressure is 55 to 90 mmHg, inclusive.

8. Females of childbearing potential** may enroll if subject has practiced adequate contraception*** $\geq$ 30 days prior to enrollment and agrees to continue adequate contraception for the entire study.

   **Child-bearing potential is defined as not sterilized via tubal ligation, bilateral oophorectomy, salpingectomy, hysterectomy, or successful Essure® placement (permanent, non-surgical, non-hormonal sterilization) with documented radiological confirmation test at least 90 days after the procedure, and still menstruating or <1 year of the last menses if menopausal.

   ***Adequate contraception includes non-male sexual relationships, abstinence from sexual intercourse with a male partner, monogamous relationship with vasectomized partner who has been vasectomized for 180 days or more prior to the subject enrollment, barrier methods such as condoms or diaphragms **with** spermicide, effective intrauterine devices, NuvaRing®, and licensed hormonal methods such as implants, injectables, or oral contraceptives ("the pill").

9. Females of childbearing potential must have a negative urine or serum pregnancy test within 24 hours prior to enrollment.
10. Drug screen for opiates is negative.
11. BMI between 18 and 40 kg/m^2.

5.1.2 Subject Exclusion Criteria

1. Have any disease or medical condition that, in the opinion of the site principal investigator or appropriate sub-investigator, is a contraindication to study participation[+].

+Including acute or chronic disease or medical condition that would place the subject at an unacceptable risk of injury, render the subject unable to meet the requirements of the protocol, or may interfere with the evaluation of responses or the subject's successful completion of this trial. These include:

- History of inflammatory bowel disease (IBD) (including ulcerative colitis, Crohn's disease, indeterminate colitis, or celiac disease).
- Irritable bowel syndrome (IBS) within the past 12 months or any active uncontrolled gastrointestinal disorders or diseases as assessed by the investigator. Including: symptoms or evidence of active gastritis or gastroesophageal reflux disease, gastric surgery, or gastric acid hyper-secretory disorders (e.g., Zollinger-Ellison syndrome), gastrointestinal obstruction, ileus, gastric retention, bowel perforation, toxic colitis, persistent infectious gastroenteritis, persistent or chronic diarrhea of unknown etiology, *Clostridium difficile* infection.
- Known active neoplastic disease^, a history of any hematologic malignancy, or have used anticancer chemotherapy/radiation therapy (cytotoxic) within 3 years prior to study enrollment.

  ^Non-melanoma, treated, skin cancers are permitted.

- Personal or family history of reactive arthritis.
- Reported history of HIV, Hepatitis B, or Hepatitis C

- History of immunodeficiency due to congenital or hereditary causes, underlying illness, or treatment.

2. Positive serology results for HLA-B27, HIV, HBsAg, or HCV antibodies.
3. Have clinically significant abnormalities as determined by study investigator in other screening laboratory tests, as outlined in the protocol.
4. Have participated in a previous *Shigella* challenge study or reports having received vaccination for *Shigella* previously.
5. Have a previously laboratory confirmed case of disease caused by *S. sonnei* or serology positive (>1:2500) for anti *S. sonnei* LPS IgG ELISA titer at screening.
6. Has a history of diarrhea in the 14 days prior to enrollment.
7. Have fewer than 3 stools per week or more than 3 stools per day as the usual frequency.
8. Recent history/current use of immunosuppressive/immunomodulating disease therapy.
9. Known hypersensitivity to ciprofloxacin or trimethoprim-sulfamethoxazole; sodium bicarbonate; or any components of vaccine, placebo, or challenge material.
10. Received or plan to receive a licensed live vaccine within 30 days prior to enrollment.
11. Received or plan to receive a licensed, inactivated vaccine, COVID-19 vaccine, or an influenza vaccine within ± 7 days from receipt of study product.
12. Have a history of severe reactions following previous immunization with any licensed or unlicensed vaccine.
13. Received Ig or other blood products (with exception of Rho D Ig) within 90 days prior to enrollment.
14. Have taken oral or parenteral (including intra-articular) corticosteroids of any dose, or high-dose inhaled corticosteroids[=] within 30 days prior to enrollment.
    [=]High-dose defined per age as using inhaled high dose per reference chart Estimated Comparative Daily Dosages
    https://www.nhlbi.nih.gov/files/docs/guidelines/asthma_qrg.pdf
15. Have taken systemic antibiotics within 7 days prior to enrollment.
16. Have taken prescription and/or OTC medication containing loperamide, acetaminophen, aspirin, ibuprofen, or other non-steroidal anti-inflammatory ≤ 48 hours prior to enrollment.
17. Have a history of alcohol or drug abuse within 1 year prior to enrollment.
18. Have been hospitalized for psychiatric illness, history of suicide attempt, or confinement for danger to self or others within 5 years prior to enrollment.
19. Work or plan to work in either a health care setting, day care center, or as a food handler or have known daily contact with individuals with possible increased susceptibility[$] to Shigella within 14 days after discharge from inpatient challenge.

    [$]Immunocompromised, elderly persons aged 70 years or more, diapered individuals, persons with disabilities, children <2 years old, a woman known to be pregnant or nursing, or anyone with diminished

immunity. Known daily contact includes contact at home, school, day-care, nursing home, or similar places.

20. Are pregnant, breastfeeding, or plan to become pregnant or breastfeed at any given time during the study.
21. Have fever or an acute illness[!] as determined by the site principal investigator or appropriate sub-investigator, within 72 hours prior to enrollment

! An acute illness which is nearly resolved with only minor residual symptoms remaining is allowable if, in the opinion of the site principal investigator or appropriate sub-investigator, the residual symptoms will not interfere with the ability to assess safety parameters as required by the protocol.

22. Received an investigational agent[&] within 30 days prior to the first study vaccination or expect to receive an investigational product during the study period which might affect safety or assessment of study endpoints.

&Including vaccine, drug, biologic, device, blood product, or medication, other than from participation in this trial.

23. Taking prescription or over-the-counter medication for weight reduction.

5.2 Withdrawal from the Study, Discontinuation of Study Product, or Study Termination

5.2.1 Withdrawal from the Study or Discontinuation of the Study Product

Subjects may voluntarily withdraw their consent for study participation at any time without penalty or loss of benefits to which they are otherwise entitled.

An investigator may also withdraw a subject from receiving the study product for any reason. Follow-up safety evaluations will be conducted if the subject agrees. If a subject withdraws or is withdrawn prior to completion of the study, the reason for this decision must be recorded in the case report forms (CRFs).

The reasons for withdrawal or discontinuation of study product, might include, but are not limited to the following:

Subject meets halting criteria

Subject becomes noncompliant

Disease or condition, or new clinical finding(s) for which continued participation, in the opinion of the investigator might compromise the safety of the subject, interfere with the subject's successful completion of this study, or interfere with the evaluation of responses

Subject lost to follow-up

Subject becomes pregnant

Determined by a physician's discretion to require additional therapy not indicated in the protocol to ensure subject's health and well-being (or treatment failure, if applicable)

The investigator should be explicit regarding study follow-up (e.g., safety follow-up) that might be carried out despite the fact the subject will not receive further study product. If the subject consents, every attempt will be made to follow all AEs through to resolution or stabilization.

The investigator will inform the subject that already collected samples and data will be retained and analyzed even if the subject withdraws from this study.

5.2.2 Handling of Withdrawals

In the case of subjects who are lost to follow-up, extensive effort (i.e., three documented contact attempts via phone calls, emails, text messages, etc., made on separate occasions and followed by a certified letter) will be made to locate or recall them, or at least to determine their health status. These efforts will be documented. Every attempt will be made to follow all AEs, including solicited systemic AEs, unsolicited non-serious AEs, and SAEs, ongoing at the time of early withdrawal through resolution as per applicable collection times defined for the specific type of AE.

Subjects who withdraw, or are withdrawn from this study, or are lost to follow-up after signing the informed consent form (ICF) and after administration of the study product will not be replaced. Subjects who withdraw, or are withdrawn from this study, or are lost to follow-up after signing the ICF but before administration of the study product may be replaced.

5.2.3 Study Termination

If the study is prematurely terminated by the sponsor, any regulatory authority, or the investigator for any reason, the investigator will promptly inform the study subjects and assure appropriate therapy or follow-up for the subjects, as necessary. The investigator will provide a detailed written explanation of the termination to the IRB/IEC. If any subject's private information will continue to be collected for this study, the IRB/IEC must approve a consent form with the study procedures, any risks and discomforts, and applicable elements, and the investigator or designee will re-consent the subjects as approved by the IRB/IEC.

5.2.4 Study Discontinuation

Each subject has the right to withdraw from the trial at any time for any reason without affecting their right to treatment by the investigator. The investigator also has the right to withdraw the subjects in the event of intercurrent illnesses, AEs, a subject's failure to comply with study procedures, or if it is determined to be in the subject's best interest. If a subject withdraws for safety reasons, an AE or SAE will be reported as described in the protocol.

If a subject withdraws for other reasons, the withdrawal will be reported as part of the annual review process. If a subject has been vaccinated but does not participate in the challenge phase, they will be discontinued and followed for safety including an approximately 6-month post vaccination study contact. If a subject chooses to withdraw from the study after challenge but before the time of planned administration of antibiotics, they will be asked to inform either the PI for the trial or an Associate investigator of the study and antibiotic therapy will be initiated. They will be encouraged to remain in the Inpatient Unit until a three-day course of treatment can be completed. If they choose not to, the study staff will observe the first dose of antibiotics and instruct the subjects how to complete the antibiotic therapy. They will also be encouraged to complete all remaining outpatient visits and be followed for safety including a 6-month study contact. No further blood samples for immunology will be collected if a subject is terminated at any time during the study.

6 STUDY PROCEDURES

6.1 Screening

After signing consent, demographic information will be obtained, and subjects will be carefully screened to ensure they are in good physical and mental health. Screening may be completed between Day -60 and Day -3 (-2), at one or more visits, depending on the subject and the timing of specimen collection.

Screening evaluations will include:

Obtaining informed consent followed by review of the inclusion/exclusion criteria, collection of demographic data, measurement of vital signs (including temperature, blood pressure, pulse, height, and weight) and collection of medical history, including concomitant medications.

An abbreviated physical examination will be performed.

Urine will be collected for health screening values. Blood will be collected to be used for health screening labs as well as baseline values for immunological response to vaccine. Stool will be collected to be used for immunological response and shedding of vaccine.

Additionally, subjects will be provided educational materials.

No subject may be screened **more than twice due to a screening failure result as defined above.** The Study Sponsor and Primary Investigator will be contacted for clarification or questions regarding screening failures. All eligibility criteria must be satisfied before a subject is enrolled.

Labs collected pre-vaccination include:

- Urine
  - Urine dip for protein and urine toxicology screen for opiates.
  - Urine pregnancy testing (human chorionic gonadotropin/ hCG) for women of child-bearing potential.
- Blood:
  - Hematology (white cell count, hemoglobin, platelets, absolute neutrophil count/ANC).
  - Chemistry (total bilirubin, ALT, creatinine, sodium, potassium).
  - Serology including HIV antibodies, HBsAg, and HCV antibodies.
  - HLA-B27.
  - Antibody (IgA and IgG) serology assays
  - ASC
  - Secondary research samples (per Appendix A1)
- Stool:
  - Culture for shedding of *Shigella*
  - Fecal IgA
  - Secondary research (per Appendix A1)

Acceptable screening values, including laboratory values are those that do not fall within Grade 2 or 3 toxicity ranges as defined in Appendix B. A low creatinine value, low bilirubin, or a low ALT value are acceptable for study inclusion as they are not considered to be clinically significant. Subjects with a laboratory abnormality may be re-screened once if there is a suspected inter-current, short-term medical illness or a suspected laboratory error. If the repeat value remains out of range but determined by the study investigator not to be clinically significant, the subject may continue the screening process.

Subjects will be provided educational materials and administered a written test of understanding. Subjects must pass with a score of at least 70%. Anyone not passing the test initially may retake the test. Subjects will have 2 chances to pass successfully. If subjects are unable to score 70% or greater on the test after the second attempt, they will be excluded from the study.

A subject may be re-screened if screened eligible previously but was not enrolled (e.g., scheduling conflict with inpatient admission days, vacation).

If screening labs are collected more than 60 days before vaccination, the screening labs may be re-collected to determine if the subject remains eligible for study participation. HLA-B27 will not be repeated on rescreen labs if drawn with initial screen.

Subjects will be informed of their screening test results by a member of the study team. Those with results that exclude them from eligibility and are sensitive in nature will be asked to return to the clinic to receive test results and counseling regarding follow-up care in the clinic.

6.2 Enrollment

Subjects who pass screening and meet all of the inclusion criteria and none of the exclusion criteria may be considered for study enrollment.

6.3 Planned Study Visits

VACCINATION VISITS

Study Day 1 (Enrollment/Vaccination Dose 1)

A two-day time period is provided to accommodate the vaccination of all eligible subjects of a cohort. However, there is no window for this visit.

Review of inclusion and exclusion criteria will be completed to confirm eligibility.

An interim medical history and review of concomitant medications will be completed, and if indicated, a targeted physical exam will be performed.

After eligibility has been confirmed, subjects will be randomized in Advantage eClinical® prior to receiving study product.

Pre-vaccination and post-vaccination reactogenicity assessment and vital signs will be obtained.

Urine will be collected within 24 hours of vaccination for βHCG pregnancy testing on females of child-bearing potential.

If not collected during the screening visit, blood and stool will be collected (prior to vaccination) for:

- **Blood**:
  - Antibody (IgA and IgG) serology assays
  - ASC
  - Secondary research (per Appendix A1)
- **Stool**:
  - Culture for shedding of *Shigella*
  - Fecal IgA
  - Secondary research (per Appendix A1)

Subjects will remain in clinic for at least 90 minutes prior to dosing and after dosing to ensure they remain NPO for at least 90 minutes before and after dose is given. Vaccination will be given orally; procedures for vaccine preparation and administration are detailed in the MOP. Subjects will remain in clinic at least 90 minutes post vaccination to also observe for immediate reactions, complete a post vaccination assessment and record any AE/SAEs on the appropriate data collection form prior to discharge from clinic.

Staff will review with subjects use of memory aid, how to complete a web based "e-Memory aid," and are expected to enter information in the e-Memory aid each day.

Subjects using the e-Memory aid will also be provided a paper memory aid for their use in the event they are unable to access the web-based system. The subjects will be asked to enter the information from the paper memory aid into the e-Memory aid once they are able to access the web-based system. The paper memory aids will not be retained as a source document.

Subjects also will be given a thermometer and instructed how to obtain a temperature and record the result.

Subjects will be asked to complete the memory aid from Day 1 through 7 days post vaccination (Day 8).

Subjects will be instructed to contact the site for any Grade 3 event, changes to health, emergency room visit or hospitalization, pregnancy, or SAE.

Study Days 1 through 7 following vaccination (Contact/Memory Aid)

Subjects will be prompted via the memory aid to complete daily reactogenicity assessment. Additionally, the memory aid will include a reminder on when the subject should collect a stool and bring the stool to clinic visits 03 (study day 4±1) and 05 (study day 8±1). (See appendix A1).

Study Day 4 ± 1 (3 days following Vaccination Dose 1)

An interim medical history and review of concomitant medications will be completed, if indicated.

Vital signs and a targeted physical exam will be performed, if either are indicated.

Staff will review and confirm memory aid information.

Subjects will be reminded to complete the memory aid.

A stool collection kit and instructions will be given to the subjects.

Review changes to health, pregnancy, AE, or SAE.

Subjects will be instructed to contact the site for any Grade 3 event, changes to health, emergency room visit or hospitalization, pregnancy, or SAE.

Samples will be collected for:

- **Blood**
  - Secondary research (per Appendix A1)
- **Stool**
  - Culture for shedding of *Shigella*
  - immunoblot and PCR
  - Secondary research (per Appendix A1)

Study Day 8 ±1 (7 days following Vaccination Dose 1)

An interim medical history and review of concomitant medications will be completed.

Vital signs and a targeted physical exam will be performed, if either are indicated.

Staff will review and confirm memory aid information.

Review changes to subject's health, pregnancy, AE, or SAE.

Subjects will be instructed to contact the site for any Grade 3 event, changes to health, emergency room visit or hospitalization, pregnancy, or SAE.

A stool collection kit and instructions will be given to the subjects, and they will be asked to bring a stool sample with them for the next study visit.

Samples will be collected for:

- **Blood**
  - Safety labs
    - Hematology, chemistry
  - ASC
  - Secondary research (per Appendix A1)
- **Stool**
  - Culture for shedding of *Shigella*
  - immunoblot and PCR
  - Fecal IgA
  - Secondary research (per Appendix A1)

Study Day 15 ±2 (14 days following Vaccination Dose 1)

An interim medical history and review of concomitant medications will be completed.

Vital signs and a targeted physical exam will be performed, if either are indicated.

Review changes to subject's health, pregnancy, AE, or SAE.

Subjects will be instructed to contact the site for any Grade 3 event, changes to health, emergency room visit or hospitalization, pregnancy, or SAE.

A stool collection kit and instructions will be given to the subjects, and they will be asked to bring a stool sample with them for the next study visit.

Samples will be collected for:

- **Blood**
  - Antibody (IgA and IgG) serology assays
  - Secondary research (per Appendix A1)
- **Stool**
  - Culture for shedding of *Shigella*
  - immunoblot and PCRFecal IgA
  - Secondary research (per Appendix A1)

Study Day 29 (+2) (Vaccination Dose 2/28 days following Vaccination Dose 1)

An interim medical history and review of concomitant medications will be completed, and if indicated, a targeted physical exam will be performed. If the subject is noted to have any disease

or medical condition that, in the opinion of the site principal investigator or appropriate sub-investigator, is a contraindication to further study participation, Dose 2 will not be given. In addition, a subject will not be given Dose 2 if any of the following are noted:

- Have diarrhea within 14 days before Dose 2
- Use of immunosuppressive/immunomodulating disease therapy since enrollment
- Received or plan to receive a licensed live vaccine within 30 days prior to Dose 2
- Received or plan to receive a licensed, inactivated vaccine, COVID-19 vaccine, or an influenza vaccine ± 7 days of Dose 2.
- Received Ig or other blood products (with exception of Rho D Ig) since enrollment
- Have taken oral or parenteral (including intra-articular) corticosteroids of any dose, or high-dose inhaled corticosteroids within 30 days before Dose 2
- Have taken systemic antibiotics within 7 days before Dose 2
- Have taken prescription and/or OTC medication containing loperamide, acetaminophen, aspirin, ibuprofen, or other non-steroidal anti-inflammatory ≤48 hours prior to Dose 2
- Have fever or an acute illness within 72 hours before Dose 2
- Received an investigational agent (including vaccine, drug, biologic, device, blood product, or medication outside of the current study) since enrollment which might affect safety or assessment of study endpoints.

If a previous AE is ongoing at dose 2, the subject may be vaccinated if the AE does not exceed the definition of AE Grade 1 and the AE is considered to be stable by a study investigator. If a Grade 2 or Grade 3 AE occurs between vaccine dose 1 and 2 and is determined not to be related to investigational product and returns to Grade 1 or lower before the next dose, the subject may receive the second dose.

Review changes to subject's health, pregnancy, AE, or SAE.

Subjects will be instructed to contact the site for any Grade 3 event, changes to health, emergency room visit or hospitalization, pregnancy, or SAE.

Pre-vaccination and post-vaccination reactogenicity assessment and vital signs will be obtained.

Samples (prior to vaccination) will be collected for:

- **Blood**
    - Antibody (IgA and IgG) serology assays
    - ASC
    - Secondary research (per Appendix A1)
- **Urine** βHCG pregnancy test (for females of child-bearing potential)
- **Stool**

- Culture for shedding of *Shigella*
- immunoblot and PCR
- Fecal IgA
- Secondary research (per Appendix A1)

Subjects will remain in clinic for at least 90 minutes prior to dosing and after dosing to ensure they remain NPO for at least 90 minutes before and after dose is given. Vaccination will be given orally; procedures for vaccine preparation and administration are detailed in the MOP. Subjects will remain in clinic at least 90 minutes post vaccination to also observe for immediate reactions and to complete a post vaccination assessment prior to discharge from clinic.

Staff will review with subjects use of memory aid, how to complete a web based "e-Memory aid" and are expected to enter information in the e-Memory aid each day.

Subjects using the e-Memory aid will also be provided a paper memory aid for their use in the event they are unable to access the web-based system. The subjects will be asked to enter the information from the paper memory aid into the e-Memory aid once they are able to access the web-based system. The paper memory aids will not be retained.

Subjects also will be given a thermometer and instructed how to obtain a temperature and record the result.

Subjects will be asked to complete the memory aid from Day 29 (±1) through 7 days post vaccination.

A stool collection kit and instructions will be given to the subjects, and they will be asked to attempt to collect stool samples per study schedule and bring them for the next study visit.

Study Day 30 through 35 following vaccination (Contact/Memory Aid)

Subjects will be prompted via the e-Memory aid to complete daily reactogenicity assessment. Additionally, the e-Memory aid will include home stool collection reminders. The memory aid will be brought to the clinic at visits 09 (study day 32±1) and 11 (study day 36±1) for review by staff. (See appendix A1).

Study Day 32±1 (3 days following Vaccination Dose 2)

An interim medical history and review of concomitant medications will be completed, if indicated.

Vital signs and a targeted physical exam will be performed, if indicated.

Staff will review and confirm memory aid information.

Subjects will be reminded to complete the memory aid.

Stool collection kits and instructions will be given to the subjects, and they will be asked to collect stool samples per study schedule and bring with them to the next study visit.

Review changes to subject's health, pregnancy, or SAE.

Subjects will be instructed to contact the site for any Grade 3 event, changes to health, emergency room visit or hospitalization, pregnancy, or SAE.

Samples will be collected for:

- **Blood**
    - Secondary research (per Appendix A1)
- **Stool**
    - Culture for shedding of *Shigella*
    - immunoblot and PCR
    - Secondary research (per Appendix A1)

Study Day 36 ±1 (7 Days Following Vaccination Dose 2)

An interim medical history and review of concomitant medications will be completed.

Vital signs and a targeted physical exam will be performed, if either are indicated.

Review changes to subject's health, pregnancy, AE, or SAE.

Subjects will be instructed to contact the site for any Grade 3 event, changes to health, emergency room visit or hospitalization, pregnancy, or SAE.

Staff will review and confirm memory aid information.

A stool collection kit and instructions will be given to the subjects, and they will be asked to bring a stool sample with them for the next study visit.

Samples will be collected for:

- **Blood**
    - Safety labs
        - Hematology, chemistry

- ASC
- Secondary research (per Appendix A1)

- **Stool**
  - Culture for shedding of *Shigella*
  - immunoblot and PCR
  - Fecal IgA
  - Secondary research (per Appendix A1)

Study Day 43 ±2 (14 days following Vaccination Dose 2)

An interim medical history and review of concomitant medications will be completed.

Vital signs and a targeted physical exam will be performed, if either are indicated.

Review changes to subject's health, pregnancy, AE, or SAE.

Subjects will be instructed to contact the site for any Grade 3 event, changes to health, emergency room visit or hospitalization, pregnancy, or SAE.

A stool collection kit and instructions will be given to the subjects, and they will be asked to bring a stool sample with them for the next study visit.

Samples will be collected for:

- **Blood**
  - Antibody (IgA and IgG) serology assays
  - Secondary research (per Appendix A1)
- **Stool**
  - Culture for shedding of *Shigella*
  - immunoblot and PCR
  - Fecal IgA
  - Secondary research (per Appendix A1)

CHALLENGE ADMISSION (Study Day 56)

Challenge will be administered 28 days (+/-2) after 2nd vaccination.

During the stay in the inpatient facility, visitors will not be permitted, and subjects will not be allowed to leave the inpatient facility until they have been cleared by a member of the study team.

Meals and any medications needed to treat clinical symptoms associated with *Shigella* illness will be provided for the study subjects during their stay in the inpatient facility. Prescribed medicines routinely taken by subjects may be taken per their usual routine and are reviewed by the investigator.

Subjects will be challenged as a single group.

Upon arrival to the inpatient unit, the subject will have SARS-CoV-2 testing performed. Subjects with a negative result will be eligible for study continuation.

Upon admission to the inpatient unit the following will be performed prior to challenge administration:

An interim medical history and review of concomitant medications will be completed. If the subject is noted to have any disease or medical condition that, in the opinion of the site principal investigator or appropriate sub-investigator, is a contraindication to further study participation, challenge will not be given. In addition, a subject will not be given challenge if any of the following are noted:

- Have diarrhea within 14 days before challenge
- Use of immunosuppressive/immunomodulating disease therapy since Dose 2
- Received or plan to receive a licensed live vaccine within 30 days prior to challenge
- Received or plan to receive a licensed, inactivated vaccine, COVID-19 vaccine, or an influenza vaccine within 7 days of challenge.
- Received Ig or other blood products (with exception of Rho D Ig) since Dose 2
- Have taken oral or parenteral (including intra-articular) corticosteroids of any dose, or high-dose inhaled corticosteroids within 30 days before challenge
- Have taken systemic antibiotics within the past 7 days before challenge
- Have taken prescription and/or OTC medication containing loperamide, acetaminophen, aspirin, ibuprofen, or other non-steroidal anti-inflammatory <48 hours prior to challenge
- Have fever or an acute illness within the past 72 hours of challenge
- Have positive SARS-CoV-2 test at the time of admission to the inpatient unit.
- Received an investigational agent (including vaccine, drug, biologic, device, blood product, or medication outside of the current study) since Dose 2 which might affect safety or assessment of study endpoints.
- Work or plan to work in either a health care setting, day care center, or as a food handler, or have known daily contact with individuals with possible increased susceptibility[$] to *Shigella* within 14 days after discharge from inpatient challenge
  [$]Immunocompromised, elderly persons aged 70 years or more, diapered individuals, persons with disabilities, children <2 years old, a woman known to be pregnant or nursing, or anyone with diminished

immunity. Known daily contact includes contact at home, school, day-care, nursing home, or similar places.

If a previous AE is ongoing at the time of challenge, the subject may be given the challenge dose if the AE does not exceed the definition of AE Grade 1 and the AE is considered to be stable by a study investigator. If a Grade 2 or Grade 3 AE occurs between vaccine dose 2 and challenge and is determined not to be related to investigational product and returns to Grade 1 or lower before the challenge, the subject may receive the challenge dose.

Perform a urine pregnancy test for women of childbearing potential (test may be done up to 24 hours prior to challenge). The pregnancy result must be negative and known prior to challenge.

Perform an abbreviated physical exam and record pre-challenge weight.

Collect vital signs including temperature, blood pressure, and pulse.

Pre-challenge samples will be collected for:

- **Blood**
  - Safety labs
    - Hematology, chemistry
  - Antibody (IgA and IgG) serology assays
  - ASC
  - Secondary research (per Appendix A2)
- **Urine** βHCG pregnancy test (for females of child-bearing potential)
- **Stool**
  - Culture for shedding of *Shigella*
  - Fecal IgA
  - Secondary research (per Appendix A2)
- SARS-CoV-2 testing

Challenge (Day 57)

Pre-challenge and post-challenge reactogenicity assessment and vital signs will be obtained.

Dietary Requirements Prior to Challenge

A light, clear liquid breakfast may be consumed the morning of challenge, then nothing by mouth at least 90 minutes before and after the Challenge Dose.

Administer 120 mL of a sodium bicarbonate solution (2 g in 120 mL of SWI) by mouth approximately five minutes before the Challenge Dose. Challenge dose will be given orally; procedures for challenge inoculum preparation and administration are detailed in the MOP.

Challenge Dose

Nothing by mouth for 90 minutes after dosing.

Procedures following the Challenge Dose

Subjects will be observed closely during the 90 minutes after receipt of the challenge by a study team member to ensure NPO status is maintained and to detect and treat any immediate adverse reactions. Vital signs will be taken at approximately 90 minutes after challenge.

Assessments and symptom directed physical examinations will be performed daily, or more often as clinically indicated, by qualified medical staff.

Assessment of reactogenicity symptoms (e.g., fever, headache, arthralgia, nausea, pain/abdominal cramps, myalgia, malaise/fatigue, anorexia/loss of appetite, chills) will be completed once in the evening of challenge administration and then at least daily until discharge. Vomiting (number of episodes per day and weights), and diarrhea (as defined per protocol) with stool consistency, number of stools per day, and stool weights during the inpatient stay will be recorded. Stool will be collected for *Shigella* detection.

Review of AEs and SAEs

Stool collection:

All stools will be classified for consistency (see Appendix D, Diarrhea Grading Classification for details), and all stools consistency classified as 3-5 (i.e., those that conform to the shape of the container) will be weighed and visually assessed for gross blood. Samples with gross blood will be confirmed by a hemoccult test.

For each subject, at least one stool sample per day will be saved for testing (the first stool produced each 24-hour period) and up to two stools with blood present by visual inspection in a 24- hour period will be tested by Hemoccult.

For each stool saved, aliquots will be made, labeled, and stored at -70 °C or colder. Any remaining stool will be disinfected with bleach in the plastic stool collector and discarded in the hazardous waste.

Inpatient Post-Challenge (Days 58-65)

Subjects will remain in the inpatient facility for a minimum of eight days following challenge.

Inpatient monitoring.

Nurses and other clinical staff will provide day and night supervision of the subjects.

Assessments and symptom directed physical examinations will be performed daily, or more often as clinically indicated, by qualified medical staff.

Vital signs (pulse, blood pressure and temperature) will be obtained at least daily, more often as clinically indicated.

If gastrointestinal illness occurs, intake and output will be measured and recorded.

If illness occurs, serum chemistry (sodium, potassium, blood urea nitrogen/BUN and creatinine) and urine specific gravity testing may be performed at the direction of the investigator and daily as indicated. Testing will be conducted until any clinically significant abnormal tests return to normal, baseline, or are acceptable.

Review of AEs and SAEs.

At least daily assessment of reactogenicity symptoms (e.g., fever, headache, arthralgia, nausea, pain/abdominal cramps, myalgia, malaise/fatigue, anorexia/loss of appetite, chills).

Antibiotic treatment will be given to subjects on the 5th day after challenge.

Review concomitant medications taken.

Collect study samples as described in table of events and as described below:

Inpatient Study Days

- **Stool** will be collected per schedule of events.
  - Every day, an aliquot of stool will be collected for testing. If no stools are passed on a given day, 1 rectal swab will be collected for culture. All stools will be classified for consistency (see Appendix D, Diarrhea Classification for details), and all stools classified as 3-5 (i.e., those that conform to the shape of the container) will be weighed and visually assessed for gross blood. Samples with gross blood will be confirmed by hemoccult test.
- **Blood** will be collected per schedule of events.
- **Nasal swab** will be collected for SARS-CoV-2 at any time a subject exhibits symptoms of COVID-19 illness during the inpatient stay.
- **Emesis** All emesis will be measured and recorded then disinfected with bleach and discarded in the hazardous waste.

Treatment of vomiting and/or diarrhea:

Subjects who develop diarrhea will be asked to drink 1.5 cc of oral rehydration solution (ORS) for each gram of diarrheal stool that they produce. Subjects developing vomiting will be asked to drink a volume of ORS equivalent to the amount of the emesis. Subjects unable to maintain their hydration status may be provided with intravenous fluids to prevent and treat dehydration. Therapies including intravenous fluids and other medications (excluding ORS) will be recorded as concomitant medications. Subjects will be allowed to continue eating solid foods as tolerable.

At the investigator's discretion and at the subject's request, subjects may be prescribed acetaminophen and/or NSAIDS (or their equivalent) for fever, headache, muscle aches, abdominal pain, or other symptoms. Loperamide (Imodium A-D®) may be prescribed if the study physician feels the subject is at high risk for volume depletion.

Anti-emetic medication may be administered by PI discretion only. Ondansetron – (Orange Book #020781, Zofran® Oral Disintegrating Tablet) or its licensed equivalent may be administered as prescribed by the investigator.

If at any time during the inpatient facility stay, the subject develops illness requiring medical care that is beyond the care available in this facility, the subject will be transferred to a hospital capable of providing this level of care. The hospital and physicians accepting the subject for care will be informed of the subject's participation in this research study so that the subject can be managed in appropriate isolation precautions.

Criteria for Discharge from Inpatient Facility

Subjects will be discharged from the inpatient unit if they have taken their antibiotic treatment, are symptom free (or improving and able to care for themselves) and passed 2 consecutive stools culture-negative for *Shigella* at least six hours apart. In the event stool cultures are positive for *Shigella*, subjects will remain in the inpatient unit until they have at least two stools that are culture-negative for the *Shigella* and will continue to have daily assessments per protocol.

Planned Discharge (Study Day 65)

Subjects will be discharged from the inpatient unit on Day 65 after they have met all of the following criteria:

Antibiotic treatment has been initiated on Day 62 (or earlier if protocol criteria are met)

Two stool samples (collected at least six hours apart) are negative for S. sonnei

Illness symptoms are resolved or resolving

The subject is able to tolerate eating and drinking

Subjects who receive IV hydration during the inpatient admission will be required to have clinically acceptable electrolyte values for sodium and potassium prior to discharge.

Subjects will be provided with verbal and written instructions, a memory aid, and thermometer to take home and record symptoms and body temperature daily starting at discharge and for the next 5 days. Staff will review with subjects' use of memory aid, how to complete a web based "e-Memory aid" and are expected to enter information in the e-Memory aid each day.

Subjects using the e-Memory aid will also be provided with a paper memory aid for their use in the event they are unable to access the web-based system. The subjects will be asked to enter the information from the paper memory aid into the e-Memory aid once they are able to access the web-based system. The paper memory aid, if used, will not be retained as a source document. The e-Memory Aid will include prompts for the subject to indicate if they have other symptoms to report or changes to their medications. Study staff will review the responses to these prompts with the subject and enter AEs or medications on the appropriate eCRFs as needed. Subjects will be instructed to take any remaining doses of study antibiotic per the written schedule provided.

Subjects will be asked to notify the study staff promptly if they develop fever, vomiting, or diarrhea during the 5 days after discharge and may be asked to return to the study site for evaluation.

Subjects will be instructed to contact the site for any Grade 3 event, changes to health, emergency room visit or hospitalization, pregnancy, or SAE.

A stool collection kit and instructions will be given to the subjects, and they will be asked to bring a stool sample with them for the next study visit.

6.3.1 Follow-up

Follow-Up Visit Post Challenge, Day 71 (14 Days post-challenge ±2)

Following discharge from the inpatient facility, subjects will return to the outpatient clinic on Day 71. (If a subject, due to persistent symptoms, remains in the inpatient unit longer than the prescribed time, study procedures may be performed while inpatient).

At this visit, the following procedures will be performed:

Review interim medical history and record concomitant medications.

Perform a targeted physical exam if indicated.

Collect vital signs, if indicated, including temperature, blood pressure, and pulse.

Review of pregnancies, AEs, and SAEs.

Subjects will be instructed to contact the site for any Grade 3 event, changes to health, emergency room visit or hospitalization, pregnancy, or SAE.

Review and confirm reactogenicity information from subject's 5-day memory aid.

Remind subject to record any unsolicited AEs through Day 85 (28 days after study challenge administration).

A stool collection kit and instructions will be given to the subjects, and they will be asked to bring a stool sample with them for the next study visit.

The following samples will be collected:

- **Blood**
  - Safety labs
    - Hematology, Chemistry
  - Antibody (IgA and IgG) serology assays
  - Secondary research (per Appendix A2)
- **Stool**
  - Fecal IgA
  - Secondary research (per Appendix A2)

Follow-up Visit Day 85 (28 Days post challenge ±2)

The following procedures will be performed at this visit:

Review interim medical history and record concomitant medications

Perform a targeted physical exam if indicated

Collect vital signs, if indicated, including temperature, blood pressure, and pulse

Review of pregnancies, AEs, and SAEs.

Subjects will be instructed to contact the site for any Grade 3 event, changes to health, emergency room visit or hospitalization, pregnancy, or SAE.

A stool collection kit and instructions will be given to the subjects, and they will be asked to bring a stool sample with them for the next study visit.

The following samples will be collected:

- Blood
  - Safety labs, if indicated
    - Hematology, Chemistry
  - Antibody (IgA and IgG) serology assays
  - Secondary research (per Appendix A2)
- **Stool**
  - Fecal IgA (per Appendix A2)

Follow-up Visit Day 113 (56 Days post challenge ±4)

Review interim medical history and concomitant medications regarding occurrence of SAEs, any other illnesses/changes to health or pregnancies.

Perform a targeted physical exam if indicated.

Collect vital signs, if indicated, including temperature, blood pressure, and pulse.

Subjects will be instructed to contact the site for any Grade 3 event, changes to health, emergency room visit or hospitalization, pregnancy, or SAE.

The following samples will be collected:

- **Blood**
  - Antibody (IgA and IgG) serology assays
  - Safety labs, if indicated
    - Hematology, Chemistry
  - Secondary research (per Appendix A2)
- **Stool**
  - Secondary research (per Appendix A2)

6.3.2 Final Study Visit

Final Study Contact Day 180 (123 days post challenge ±14)

On Day 180, subjects will complete a final safety contact by phone or email to find out about the occurrence of any pregnancies or SAE.

6.3.3 Early Termination Visit

If, after admission to the inpatient unit and receipt of the *Shigella* challenge, a subject decides to withdraw consent and leave the unit early, he/she will be counseled about the risk of transmission of *Shigella* to close contacts. Prior to leaving the unit, the subject will be required to

be directly observed by the study staff to swallow the 500 mg dose of ciprofloxacin. The subject will be sent home with five additional 500 mg tablets of ciprofloxacin to complete the 3-day course of therapy at home. Subjects with contraindication to ciprofloxacin may receive treatment trimethoprim-sulfamethoxazole (160 mg/800 mg twice daily for 5 days). Prior to leaving the unit, the subject will be required to be directly observed by the study staff to swallow the first dose of trimethoprim-sulfamethoxazole. The subject will be sent home with 9 additional tablets of trimethoprim-sulfamethoxazole to complete the 5-day course of therapy at home. This will be documented in the subject source document.

If the subject needs to be transferred to a hospital for additional care during his/her inpatient facility stay, the study staff will ask permission of the subject to obtain additional medical records to follow the subject until the symptoms of this SAE resolves or the subject's condition becomes stable.

Regardless of when a subject decides to withdraw consent, it will be requested that the subject return for safety evaluations prior to withdrawal.

6.4 Unscheduled Study Visits

Subjects will be asked to notify the study staff promptly if they develop any illness suggestive of recurrence of *Shigella* infection (vomiting and/or diarrhea) or possible dehydration. If the study staff determines the symptoms are potentially significant, the subject will be asked to come to the clinic for an evaluation. Subjects will be asked to complete an unscheduled visit for any event that warrants follow-up. Per PI discretion, safety labs may be collected to further evaluate subject well-being. All events will be followed to resolution or until determined to be stable.

6.5 Protocol Deviations

A protocol deviation is any noncompliance with the clinical trial protocol, cGCP, or protocol-specific MOP requirements. The noncompliance may be either on the part of the subject, the site principal investigator, or the site personnel. As a result of deviations, corrective actions are to be developed by the site and implemented promptly.

These practices are consistent with ICH E6:

4.5 Compliance with Protocol, Sections 4.5.1, 4.5.2, and 4.5.3

5.1 Quality Assurance and Quality Control, Section 5.1.1

5.20 Noncompliance, Sections 5.20.1, and 5.20.2.

It is the responsibility of the site principal investigator and personnel to use continuous vigilance to identify and report deviations within five working days of identification of the protocol

deviation, or within five working days of the scheduled protocol-required activity. All deviations must be promptly reported to DMID per the DCC protocol deviation reporting procedures.

All protocol deviations, as defined above, must be addressed in study subject case report forms. A completed copy of the DMID Protocol Deviation Form must be maintained in the Regulatory File, as well as in the subject's chart. Protocol deviations must be sent to the local IRB/IEC per their guidelines. The site principal investigator and personnel are responsible for knowing and adhering to their IRB requirements.

7 DESCRIPTION OF CLINICAL AND LABORATORY EVALUATIONS

7.1 Clinical Evaluations

Following consent, a screening evaluation will be performed.

Medical History

Medical history will be obtained by direct interview and will include a review of concomitant medications, supplements, and over-the-counter medications. Subjects will be queried regarding a history of significant medical disorders of the head, eyes, ears, nose, throat (HEENT), mouth, cardiovascular system, lungs, gastrointestinal tract, liver, pancreas, kidney, urologic, nervous system, blood, lymph glands, endocrine system, musculoskeletal system, skin, and genital/ reproductive tract. A history of any allergies, cancer, gastrointestinal disorders, immunodeficiency, psychiatric illness, substance abuse, and autoimmune disease will be solicited.

Physical Examination

An abbreviated physical examination will be conducted at the screening visit. An abbreviated physical examination is distinguished from a complete physical exam in that all assessments are not required (e.g., genito-urinary and rectal exams). A rectal examination may be performed at screening if, in the opinion of the investigator, this assessment is warranted based on the subject's medical history. A targeted physical examination may be performed at all other study visits if indicated based on assessment, reactogenicity, or interim medical history review.

Inpatient Evaluations

During the Inpatient Stay, the following clinical evaluations will occur:

Assessments and targeted physical examinations will be performed daily, or more often as clinically indicated, by qualified medical staff.

Assessment of reactogenicity symptoms will be completed every evening beginning post challenge through discharge.

Vomiting (number of episodes per day and weights) and diarrhea (as defined per protocol) with stool consistency, number of stools per day and stool weights during the inpatient stay will be recorded. Stool will be collected for *Shigella* detection and stools with gross blood will be tested for occult blood.

Subjects will be asked to collect every stool passed during the inpatient portion of the study.

Pre-challenge body weight will be measured on admission to the inpatient unit and repeated during the inpatient stay as clinically indicated to evaluate for dehydration.

Vital signs including oral temperature, blood pressure and pulse will be obtained at least daily.

Daily evaluations will be performed by an investigator, or more frequently as clinically indicated.

Evaluation for dehydration. A clinician designated on the 1572 will evaluate subjects who are symptomatic and have any of the following:

- Tachycardia (resting heart rate >100 beats/min).
- Hypotension (systolic pressure <90 mm Hg or diastolic pressure <60 mm Hg accompanied by symptoms of orthostasis).
- Greater than 1000 mL deficit in ORS replacement in 24 hours.

Illness labs may be collected at the discretion of the investigator (serum chemistry, including sodium and potassium, blood urea nitrogen/BUN, and creatinine) and as indicated thereafter until symptoms of gastroenteritis resolve and any clinically significant abnormal tests return to normal, baseline, or are acceptable. A urine dipstick may be performed to capture urine specific gravity results in real time to help determine hydration status of a subject.

7.1.1 Assessment of Concomitant Medications/Treatments other than Study Product

Administration of any medications or vaccines will be documented in the appropriate eCRF. All concomitant medications, taken in the 30 days prior to study enrollment through Day 28 following challenge or early termination, whichever occurs first, will be recorded. All prescription and over-the-counter medications as well as vitamins and supplements will be recorded.

Medications which may interfere with the evaluation of the investigational product should not be used unless absolutely necessary. Assessment of whether the subject qualifies to receive the dose will include a review of permitted and prohibited medications as described in flow of study visit.

Taking prescription or over-the-counter weight-loss medication is prohibited for the duration of the study and is exclusionary for enrollment.

Use of new medications should prompt evaluation for the presence of an AE or new chronic medical condition.

Any medications considered for treatment of fever, AE, or reactogenicity will be given only at the discretion of a study investigator.

7.1.2 Non-Research Standard of Care

If at any time during the inpatient facility stay, the subject develops illness requiring medical care that is beyond the care available in this facility, the subject will be transferred to a hospital capable of providing this level of care. The hospital and physicians accepting the subject for care will be informed of the subject's participation in this research study so that the subject can be managed in appropriate isolation precautions.

7.2 Laboratory Evaluations

7.2.1 Clinical Laboratory Evaluations

Safety laboratory evaluations will be performed by the local lab of the performance site and sites will follow the FDA toxicity table (see Appendix B) for guidance.

Clinical safety lab testing will include hematology, serology, and chemistry.

Hematology: white blood cell count (WBC), absolute neutrophil count (ANC), hemoglobin (Hgb), and platelet count

Chemistry: sodium, potassium, creatinine, ALT, total bilirubin, and blood urea nitrogen/BUN if indicated during the inpatient admission.

Subjects who receive IV hydration during the inpatient admission will be required to have clinically acceptable electrolyte values for sodium and potassium prior to discharge.

Urine pregnancy testing will be performed in the clinic by a qualified study team member using a commercially available test.

For females of childbearing potential, a urine pregnancy test will be performed at screening. Urine pregnancy testing will be performed within 24 hours before vaccination and challenge

administration. Pregnancy test results must be negative for subjects to be eligible for enrollment and qualify to receive vaccination and challenge doses.

Subjects will be tested for HLA-B27, HIV, Hepatitis B surface antigen, and antibody to Hepatitis C at screening only. Any subject having HIV, Hepatitis B, or Hepatitis C, or positive HLA-B27 will not be enrolled into the study.

During screening urinalysis will be done to test for urine protein via dipstick in the clinic. Clean-catch, mid-stream, urine specimen will be collected in a sterile urine cup. Urine specific gravity may be performed during the inpatient admission, if indicated.

Urine test for opiates: A clean-catch, mid-stream urine specimen will be collected in a sterile urine cup and transported to the clinical laboratory for processing and examination

A SARS-CoV-2 test will be performed on all subjects upon entry into the challenge unit. Anyone with a positive test will remain quarantined in their room until it is medically safe for the subject to be escorted off the inpatient unit. In addition, a SARS-CoV-2 test will be performed at any time during the inpatient stay if the participant is demonstrating symptoms consistent with SARS-CoV-2.

Stool Culture, immunoblot and PCR:

The local laboratory at each site, using fresh stool samples, will conduct an assay for the presence of *S. sonnei*. The first procedure is qualitative and involves streaking swabs containing fecal material to Hektoen Enteric Agar (HEA) plates. All HEA plates will be incubated at 37±1° C overnight. For each subject from the qualitative cultures, up to two blue-green (non-lactose fermenting) colonies will be picked from swab-streaked HEA plate and tested by agglutination by *S. sonnei* polyvalent Group D antiserum. Colonies that agglutinate will be recorded as potential positive for presence of *S. sonnei*. The second procedure (to be performed at WRAIR) is quantitative (determined by immunoblot) and involves plating serial dilutions of frozen-thawed stool suspensions (in BGS) to HEA plates. HEA plates used for the serial dilutions that contain blue-green colonies will be processed using a colony blot procedure specific for the detection of *S. sonnei*. The number of positive colonies for each dilution will be recorded and used to calculate the cfu per gram of stool. These procedures will follow the MOP for culture isolation, immunoblot and identification for *S. sonnei* from stool specimens. Stool will be analyzed at WRAIR by PCR to detect WRSs2 vaccine and challenge strain *S. sonnei* 53G.

Microbiology and Immunology:

Biosamples (serum, PBMCs, stool) for immunological assays will be collected, processed, cryopreserved and stored as detailed in the protocol MOP. All immunological assays will be

performed in batches after all samples have been collected for a given assay within a cohort. To minimize variability, all pre- and post-vaccination/challenge samples of a subject will be assayed on the same day as detailed in the MOP.

Antibody secreting cells (ASC):

At times specified in the protocol and the MOP, the peripheral blood mononuclear cells (PBMCs) will be isolated from whole blood and cryopreserved to be used in ASC. An ELISPOT assay will be used to enumerate *S. sonnei* LPS and *S. sonnei* Invaplex specific IgA and IgG ASCs per 10^6 PBMC. The IgA and IgG ASC assays will be performed at CCHMC.

Serology:

Blood will be collected for IgA and IgG assay by ELISA[25]. The serum will be separated and stored frozen for determination of specific antibody responses against *S. sonnei* LPS and Invaplex antigens. The serum IgA and IgG ELISA assays will be performed at CCHMC.

Fecal IgA:

The stool sample (4-5gm) will be placed in a 30 mL Oakridge tube and frozen at -70ºC or colder until ready for extraction using soybean trypsin inhibitor-EDTA procedure. Total and *Shigella*-antigen specific IgA will be measured in stool extracts using ELISA procedures. Fecal total and antigen-specific IgA ELISA assays will be performed at CCHMC.

7.2.2 Secondary Research Assays

Stool and blood will be preserved for future studies. Details of assays, methodology and location of testing will be determined at a later date.

7.2.3 Laboratory Specimen Preparation, Handling, and Storage

Whole blood will be collected by the venous route using aseptic techniques. Depending on the testing performed, aliquots of blood will be sent to the local Clinical Laboratory and/or the site’s local research for processing.

Stool samples will be collected using specially designed stool collection kits. Study team members will use appropriate contact isolation procedures to minimize their risk of handling the stool specimens. If visible spillage is present on the outside of the stool container, the container will be wiped with an appropriate disinfectant and dried prior to the study labels being placed on the tubes. Any spillage will be handled in the same manner. The samples will then be placed in sealed specimen bags and sent to the site’s local research laboratory for processing and storage until testing is performed. Instructions for Specimen Preparation, Handling, and Storage

Instructions for specimen preparation, handling, and storage are included in the protocol-specific MOP. Instructions for specimen shipment are included in the protocol-specific MOP.

7.2.4 Laboratory Specimen Shipping

Instructions for Specimen Preparation, Handling, and Storage Instructions for specimen preparation, handling, and storage are included in the protocol-specific MOP. Instructions for specimen shipment are included in the protocol-specific MOP.

8 ASSESSMENT OF SAFETY

8.1 Assessing and Recording Safety Parameters

Safety will be assessed by the frequency and severity of:

1. SAEs occurring from the time of the first vaccination through Day 180.
2. Solicited AEs – reactogenicity events occurring from the time of each study vaccination through 7 days following vaccination doses:
    a. Reactions following vaccination to include headache, arthralgia, nausea, vomiting, diarrhea, pain/abdominal cramps, myalgia (body aches, muscular pain), malaise/fatigue, anorexia/loss of appetite, chills, and fever
3. Solicited AEs- from time of challenge through 5 days after discharge following challenge administration.
    a. Reactions following challenge to include headache, arthralgia, nausea, pain/abdominal cramps, myalgia (body aches, muscular pain), malaise/fatigue, anorexia/loss of appetite, chills, vomiting, diarrhea, and fever.
4. Unsolicited AEs – non-serious events occurring from the time of each vaccination and challenge through approximately 28 days post vaccination doses and challenge administration.
5. Clinical safety laboratory AEs occurring from the time of each study vaccination through 7 days following vaccination doses and challenge administration, respectively: Parameters to be evaluated include WBC, ANC, hemoglobin, platelet count, potassium, sodium, creatinine, ALT, and total bilirubin.

The memory aid will be used to collect all solicited and unsolicited AEs, and medications for the first 7 days after each vaccination starting the evening of administration of the vaccine/placebo. From 8 days until 28 days following each vaccination, only unsolicited AEs, solicited adverse reactions that continue beyond the 7th day, and related medications will be collected during follow up.

Subjects will be asked to contact the study site for fever, any Grade 3 event, emergency room visits, hospitalizations, pregnancy, or changes in their baseline health since vaccination.

8.1.1 Adverse Events (AEs)

Adverse Event (AE): (ICH) E6 defines an AE as any untoward medical occurrence in a patient or clinical investigation subject administered a pharmaceutical product regardless of its causal relationship to the study treatment. FDA defines an AE as any untoward medical occurrence associated with the use of a drug in humans, whether or not considered drug related.

An AE can therefore be any unfavorable and unintended sign (including an abnormal laboratory finding), symptom, or disease temporally associated with the use of medicinal (investigational) product. The occurrence of an AE may come to the attention of study personnel during study visits and interviews of a study recipient presenting for medical care, or upon review by a study monitor.

Beginning on the day of the first dose of study agent, AEs, including solicited systemic (subjective and quantitative) reactions, will be captured on the appropriate eCRF. Information to be collected for unsolicited non-serious AEs includes event description, date of onset, assessment of severity, relationship to study product and alternate etiology (assessed only by those with the training and authority to make a diagnosis and listed on the Form FDA 1572 as an investigator), date of resolution or stabilization, seriousness, and outcome. AEs occurring during the trial collection and reporting period will be documented appropriately regardless of relationship. AEs will be followed through resolution or stabilization.

Any medical condition that is present at the time that the subject is screened and does not exceed the protocol definition of a Grade 1 AE, will be considered as baseline, documented as medical history for the subject, and will not be reported as an AE. However, if at any visit after administration of the study product the severity of any pre-existing medical condition increases or is graded greater than a Grade 1, it will be recorded as an AE.

8.1.2 AE Grading

All AEs (laboratory and clinical symptoms) will be graded for severity and assessed for relationship to study product (see definitions). AEs characterized as intermittent require documentation of onset and duration of each episode. The start and stop date of each reported AE will be recorded on the appropriate eCRF.

Severity of Event

AEs will be assessed by the investigator using a protocol-defined grading system (toxicity table included as an appendix). For events not included in the protocol-defined grading system, the following guidelines will be used to quantify severity:

- Mild (Grade 1): Events that are usually transient and may require only minimal or no treatment or therapeutic intervention and generally do not interfere with the subject's usual activities of daily living.
- Moderate (Grade 2): Events that are usually alleviated with additional specific therapeutic intervention. The event interferes with usual activities of daily living, causing discomfort but poses no significant or permanent risk of harm to the research subject.
- Severe (Grade 3): Events interrupt usual activities of daily living, or significantly affects clinical status, or may require intensive therapeutic intervention. Severe events are usually incapacitating.

AEs will also be graded according to the following Grading Scales:

- Appendix B: Toxicity Table/Laboratory AE Grading Scale
- Appendix C: Vital Signs AE Grading Scale
- Appendix D: Diarrhea Classification
- Appendix E: Reactogenicity Grading Scale

AEs characterized as intermittent require documentation of onset and duration of each episode. Changes in the severity of an AE will be documented to allow an assessment of the duration of the event at each level of intensity.

Relationship to Study Products

The assessment of the relationship of an AE to the administration of study product is made only by those with the training and authority to make a diagnosis and listed on the Form FDA 1572 as an investigator based on all available information at the time of the completion of the eCRF. Whether the AE is related or not, is not a factor in determining what is or is not reported in this trial. If there is any doubt as to whether a clinical observation is an AE, the event should be reported.

In a clinical trial, the study product must always be suspect. To help assess, the following guidelines are used.

- Related – There is a reasonable possibility that the study product caused the AE. Reasonable possibility means that there is evidence to suggest a causal relationship between the study product and the AE.

- Not Related – There is not a reasonable possibility that the administration of the study product caused the event.

8.1.3 Reactogenicity

Reactogenicity is AEs that are common and known to or expected to occur following the administration of the study vaccination and challenge products. Subjects will be assessed for reactogenicity at baseline and following vaccination/placebo administration and following challenge administration.

The Toxicity Grading Scales to be used to guide the grading of reactogenicity are in Appendix D and Appendix E.

8.1.4 Serious Adverse Events (SAEs)

An AE or suspected adverse reaction is considered "serious" if, in the view of either the investigator or sponsor, it results in any of the following outcomes:

Death

A life-threatening AE1

Inpatient hospitalization or prolongation of existing hospitalization

A persistent or significant incapacity or substantial disruption of the ability to conduct normal life functions

A congenital anomaly/birth defect

Important medical events that may not result in death, be life-threatening, or require hospitalizations may be considered serious when, based upon appropriate medical judgment they may jeopardize the patient or subject and may require medical or surgical intervention to prevent one of the outcomes listed in this definition. Examples of such medical events include allergic bronchospasm requiring intensive treatment in an emergency room or at home, blood dyscrasias or convulsions that do not result in inpatient hospitalization, or the development of drug dependency or drug abuse.

[1] Life-threatening AE. An AE is considered "life-threatening" if, in the view of either the site principal investigator or sponsor, its occurrence places the patient or subject at immediate risk of death. It does not include an AE, had it occurred in a more severe form, might have caused death.

SAEs will be:

Assessed for severity and relationship to study product and alternate etiology

Recorded on the appropriate SAE data collection form and eCRF

Followed through resolution or stabilization

Reviewed and evaluated by the DSMB (periodic review unless related), DMID, and the IRB

8.2 Specification of Safety Parameters

Safety will be based on the frequency and severity of:

8.2.1 Solicited Events

Solicited Events are AEs that are common and known to or expected to occur following the administration of the study product. Subjects will be assessed for AEs following vaccination and challenge administration (see Appendix E) and will complete an e-memory aid to record symptoms for seven days following each vaccination and five days after discharge from the isolation facility. During the in-patient phase, solicited events will be reviewed and assessed by the clinical research team. The subjects will be provided with a thermometer. The subject e-memory aids will be reviewed with the subject at subsequent visits. Any symptoms still present after the solicited event collection period (Day 8 post vaccination doses and 5 days post discharge from the inpatient isolation unit) will continue to be followed until resolution or determined to be stable per investigator.

For baseline laboratory results that are abnormal according to the local laboratory reference range and fall within Grade 1 toxicity table range, these will not be considered laboratory AE and will thus not be graded. However, if baseline clinical labs fall within Grade 1 range, then a laboratory AE is reported only if the value changes such that it falls into Grade 2 or higher when subsequent safety laboratory testing is done.

8.2.2 Unsolicited Events

Unsolicited, or unexpected events, are any other AEs that occur following administration of vaccination doses or challenge. Subjects will be asked to record any unsolicited AEs through Day 29 after receipt of vaccine and challenge study product administration. AEs will be followed until resolution or determined to be stable per investigator discretion.

8.3 Reporting Procedures

8.3.1 Reporting SAEs

SAEs will be followed until resolution or stabilization even if this extends beyond the study-reporting period. Resolution of an AE is defined as the return to pretreatment status or stabilization of the condition with the expectation that it will remain chronic.

Any AE that meets a protocol-defined serious criterion must be submitted immediately (within 24 hours of site awareness) on an SAE form to the DMID Pharmacovigilance Group, at the following address:

DMID Pharmacovigilance Group

Clinical Research Operations and Management Support (CROMS)

6500 Rock Spring Dr. Suite 650

Bethesda, MD 20817, USA

SAE Hot Line: 1-800-537-9979 (US) or 1-301-897-1709 (outside US)

SAE FAX Number: 1-800-275-7619 (US) or 1-301-897-1710 (outside US)

SAE Email Address: PVG@dmidcroms.com

In addition to the SAE form, select SAE data fields must also be entered into the DCC system (for example: Advantage eClinical®). Please see the protocol-specific MOP for details regarding this procedure.

Other supporting documentation of the event may be requested by the DMID Pharmacovigilance Group and should be provided as soon as possible.

The DMID Medical Monitor and DMID Clinical Project Manager will be notified of the SAE by the DMID Pharmacovigilance Group. The DMID Medical Monitor will review and assess the SAE for regulatory reporting and potential impact on study subject safety and protocol conduct.

At any time after completion of the study, if the site principal investigator or appropriate sub-investigator becomes aware of an SAE that is suspected to be related to study product, the site principal investigator or appropriate sub-investigator will report the event to the DMID Pharmacovigilance Group.

8.3.2 Regulatory Reporting for Studies Conducted Under DMID-Sponsored IND

Following notification from the investigator, DMID, the Investigational New Drug (IND) sponsor, will report any suspected adverse reaction that is both serious and unexpected. DMID will report an AE as a suspected adverse reaction only if there is evidence to suggest a causal relationship between the drug and the AE. DMID will notify FDA and all participating investigators (i.e., all investigators to whom the sponsor is providing drug under its IND(s) or under any principal investigator's IND(s)) in an IND safety report of potential serious risks from clinical trials or any other source, as soon as possible, but in no case later than 15 calendar days after the sponsor determines that the information qualifies for reporting as specified in 21 CFR

Part 312.32. DMID will also notify FDA of any unexpected fatal or life-threatening suspected adverse reaction as soon as possible, but in no case later than 7 calendar days after the sponsor's initial receipt of the information. Relevant follow-up information to an IND safety report will be submitted as soon as the information is available. Upon request from FDA, DMID will submit to FDA any additional data or information that the agency deems necessary, as soon as possible, but in no case later than 15 calendar days after receiving the request.

All serious events designated as "not related" to study product(s), will be reported to the FDA at least annually in a summary format.

8.3.3 Reporting of Pregnancy

Pregnancy is not an AE but is a collectible event. Pregnancies occurring in study subjects will be reported in IDES. Pregnancy will be confirmed with serum pregnancy testing. No further vaccinations or challenge will be administered to pregnant subjects. With the subject's permission all planned study samples will be collected and the subject will continue in follow-up for safety events. Efforts will be made to follow all pregnancies reported during the study to pregnancy outcome with the subject's permission.

8.4 Type and Duration of Follow-up of Subjects after AEs

All AEs and SAEs will be followed to resolution or until determined to be stable.

AEs will be assessed and followed from initial recognition of the AE through end of the protocol defined follow-up period.

SAEs will be followed up through resolution even if duration of follow-up goes beyond the protocol-defined follow-up period.

Resolution of an AE is defined as the return to pre-treatment status or stabilization of the condition with the expectation that it will remain chronic.

8.5 Procedures to be Followed in the Event of Abnormal Laboratory Test Values or Abnormal Clinical Findings

The site principal investigator or appropriate sub-investigator is responsible for recording all AE/SAEs that are observed or reported during this trial, regardless of the relationship to study product. AE/SAEs, abnormal laboratory test values, or abnormal clinical findings will be collected, assessed, documented, reported, and followed appropriately. Grading of the laboratory AEs will be based on the Appendix B toxicity table.

8.6 Study Halting Rules

Further study enrollment and dosing at $5x10^5$ will be halted for DSMB review/recommendation if any of the following Halting Rules are met at this dose:

Death of an enrolled subject or any SAE if the SAE is considered by the Medical Monitor to be related to the study products (WRSs2 or *S. sonnei* 53G), at any time during the study

Any Grade 3 AE (of the same parameter or MedDRA Preferred Term) in two subjects prior to challenge

Any Grade 3 AE (of the same parameter or MedDRA Preferred Term) in two subjects post-challenge, unless it is expected due to shigellosis (diarrhea, vomiting, fever, nausea, abdominal pain/cramping, myalgia/arthralgia, headache, anorexia, chills, and malaise).

The study may also be suspended because of safety findings such as an overall pattern of symptomatic, clinical, or laboratory events that the Medical Monitor considers related to the study products (WRSs2 or *S. sonnei* 53G) and that may appear minor in terms of individual events, but that may collectively represent a serious potential concern for safety

8.6.1 Individual Halting Criteria

A subject may be removed from the study if an investigator deems it in the best interest of the subject. A subject also may halt (discontinue participation) at any time. If a subject meets an individual halting criterion, it will not halt the study but halt the subject’s involvement.

8.7 Safety Oversight (DSMB, as applicable)

Safety oversight will be conducted by a Data and Safety Monitoring Board (DSMB) which is an independent group of experts that monitors subject safety and advises the IND sponsor and study investigators.

8.7.1 Data and Safety Monitoring Board (DSMB)

Safety oversight will be conducted by a DSMB that is an independent group with expertise to interpret data from this study and will monitor subject safety and advise DMID. The DSMB members will be separate and independent of study personnel participating in this study and should not have scientific, financial, or other conflict of interest related to this study. DSMBs must consist of at least three voting members, including a biostatistician experienced in statistical methods for clinical trials and a clinician with relevant expertise.

The DSMB will review safety data at the following time points:

Before study initiation and after all challenge cohorts have been completed

Ad hoc when a halting rule is met or as needed

At least annually

Final review meeting: 6 to 8 months after clinical database lock to review the cumulative unblinded safety data for the study. The data will be provided in a standard summary format. The DSMB may be asked to provide recommendations in response to questions posed by the IND sponsor.

Procedures for DSMB reviews/meetings will be defined in a charter. The DSMB will review applicable data to include but not limited to enrollment, demographic information, dosing, laboratory, and safety data which may include solicited and unsolicited AE/SAEs, concomitant medications, medical history, clinical laboratory values and any physical examinations. Safety data will be reviewed by the DSMB per the DSMB charter for this study. The DSMB may receive data in aggregate and presented by group. The DSMB may be unblinded to study group assignment, as needed, to assess safety issues. As an outcome of each review/meeting, the DSMB will make a recommendation as to the advisability of proceeding with vaccinations (as appropriate), and to continue, modify or terminate the study.

The DSMB will review data at specified times during the course of the study for subject and overall study progress and will conduct ad hoc reviews as appropriate when a halting rule is met or for immediate concerns regarding observations during this study.

The IND sponsor or the DSMB chair may convene the DSMB on an ad hoc basis according to protocol criteria or if there are immediate concerns regarding observations during the course of the study. The Medical Monitor is empowered to stop the study at any time if AEs that meet the halting criteria are reported. The Medical Monitor will be responsible for reviewing SAEs in real time.

9 HUMAN SUBJECTS PROTECTION

9.1 Institutional Review Board/Independent Ethics Committee

Each site principal investigator will obtain IRB approval for this protocol to be conducted at his/her research site(s) and send supporting documentation to the DMID before initiating recruitment of subjects. The investigator will submit applicable information to the IRB/IEC on which it relies for the review, to conduct the review in accordance with 45 CFR 46, ICH E6 GCP, and as applicable, 21 CFR 56 (Institutional Review Boards) and 21 CFR 50 (Protection of Human Subjects), other federal, state, and local regulations. The IRB/IEC must be registered with OHRP as applicable to the research. DMID must receive the documentation that verifies IRB/IEC-approval for this protocol, associated informed consent documents, and upon request any recruitment material and handouts or surveys intended for the subjects, prior to the recruitment and enrollment of subjects.

Any amendments to the protocol or consent materials will be approved by the IRB/IEC before they are implemented. IRB/IEC review and approval will occur at least annually throughout the enrollment and follow-up of subjects and may cease if annual review is no longer required by applicable regulations and the IRB/IEC. The investigator will notify the IRB/IEC of deviations from the protocol and reportable SAEs, as applicable to the IRB/IEC policy.

Each institution engaged in this research will hold a current Federal-wide Assurance (FWA) issued by the Office of Human Research Protection (OHRP) for federally funded research.

9.2 Informed Consent Process

Informed consent is a process that is initiated prior to an individual agreeing to participate in a trial and continuing throughout the individual’s trial participation. Before any study procedures are performed, informed consent will be obtained and documented. Subjects will receive a concise and focused presentation of key information about the clinical trial, verbally and with a consent form. The explanation will be organized and presented in lay terminology and language that facilitates understanding why one might or might not want to participate.

An investigator or designee will describe the protocol to potential subjects face-to-face. The key information about the purpose of the study, the procedures and experimental aspects of the study, risks and discomforts, any expected benefits to the subject, and alternative treatment will be presented first to the subject.

Subjects will also receive an explanation that the trial involves research, and a detailed summary of the proposed study procedures and study interventions/products. This will include aspects of

the trial that are experimental, the probability for random assignment to treatment groups, any expected benefits, all possible risks (including a statement that the particular treatment or procedure may involve risks to the subject or to the embryo or fetus, if the subject is or may become pregnant, that are currently unforeseeable), the expected duration of the subject's participation in the trial, alternative procedures that may be available and the important potential benefits and risks of these available alternative procedures.

Subjects will be informed that they will be notified in a timely manner if information becomes available that may be relevant to their willingness to continue participation in the trial. Subjects will receive an explanation as to whether any compensation and any medical treatments are available if injury occurs, and, if so, what they consist of, or where further information may be obtained. Subjects will be informed of the anticipated financial expenses, if any, to the subject for participating in the trial, as well as any anticipated prorated payments, if any, to the subject for participating in the trial. They will be informed of whom to contact (e.g., the investigator) for answers to any questions relating to the research project.

Information will also include the foreseeable circumstances and/or reasons under which the subject's participation in the trial may be terminated. The subjects will be informed that participation is voluntary and that they are free to withdraw from the study for any reason at any time without penalty or loss of benefits to which the subject is otherwise entitled.

The extent of the confidentiality of the subjects' records will be defined, and subjects will be informed that applicable data protection legislation will be followed. Subjects will be informed that the monitor(s), auditors(s), IRB, NIAID, and regulatory authority(ies) will be granted direct access to the subject's original medical records for verification of clinical trial procedures and/or data without violating the confidentiality of the subject, to the extent permitted by the applicable laws and regulations, and that, by signing an informed consent form, the subject is authorizing such access.

Subjects will be informed that records identifying the subject will be kept confidential, and, to the extent permitted by the applicable laws and/or regulations, will not be made publicly available and, if the results of the trial are published, the subject's identity will remain confidential. Subjects will be informed whether private information collected from this research and/or specimens will be used for additional research, even if identifiers are removed.

Subjects will be allowed sufficient time to consider participation in this research trial and have the opportunity to discuss this trial with their family, friends, or legally authorized representative, or think about it prior to agreeing to participate.

Informed consent forms will be IRB approved and subjects will be asked to read and review the consent form. Subjects must sign the informed consent form prior to starting any study procedures being done specifically for this trial.

Once signed, a copy of the informed consent form will be offered to the subject(s) for their records. The subject(s) may withdraw their consent at any time during the trial. The rights and welfare of the subject(s) will be protected by emphasizing to them that the quality of their medical care will not be adversely affected if they decline to participate in this study.

Study personnel may employ recruitment efforts prior to obtaining study consent if a patient-specific screening consent is on record or if the IRB has agreed that chart review is allowed without a fully executed screening consent. In cases where there is not a patient-specific screening consent on record, site Clinical staff may pre-screen via chart review and refer potential subjects to the Research staff. Research staff would obtain consent per the standard informed consent process before conducting protocol-specific screening activities.

New information will be communicated by the site principal investigator to subjects who consent to participate in this trial in accordance with IRB requirements. The informed consent document will be updated, and subjects will be re-consented per IRB requirements, if necessary. Subjects will be offered a copy of all informed consent forms that they sign.

9.3 Consent for Secondary Research of Residual Specimens and Data

Residual samples are samples that are left over after the study has been completed and are stored for possible use in future research studies. Retention of residual samples for secondary research and the collection and storage of samples for use in future research studies is a condition of study participation. Subjects who sign the informed consent form for the study are consenting to allow the collection, storage, and use of both residual and secondary research samples.

Residual samples will be maintained for possible use in future research studies, such as examining additional immunological assessments or testing for antibodies against other viruses or bacteria. These samples may be shared for purposes other than per protocol analysis with investigators at the participating VTEU site and with other investigators at other institutions once the clinical study report has been finalized.

Secondary research samples are also being collected and will not become part of a subject's medical record and will not be shared with their doctor. Secondary research studies may include but are not limited to non-traditional immune assay development, assessing innate immune factors, cytokines and other virologic evaluations.

Residual and secondary research samples will be stored indefinitely at a DMID designated central clinical storage facility. These samples will not be sold or used directly for production of any commercial product. Each sample will be encoded (labeled) only with a barcode and a unique tracking number to protect subject confidentiality. There are no benefits to subjects in the collection, storage, and subsequent use of their specimens for future research. Reports about future research done with subjects' samples will NOT be kept in their health records.

Residual samples will be available upon the completion of the study; however, secondary research samples may be requested from DMID and shipped from the DMID CMS at any time.

Subjects may change their decision to participate in the study at any time by notifying the study doctors or nurses in writing. However, any data from a previously collected sample prior to the withdrawn consent will not be removed. Any secondary research of residual samples or collection of samples specifically for future research not yet collected/stored will not be stored for future/residual use. Any data from a previously collected sample prior to the withdrawn consent will not be removed.

9.4 Exclusion of Women, Minorities, and Children (Special Populations)

Special populations, e.g., non-English speakers, children, illiterate or non-writing individuals and vulnerable populations will not be enrolled in this study.

9.5 Subject Confidentiality

Subject confidentiality is strictly held in trust by the participating investigators, their staff, and the sponsor(s) and their agents. This confidentiality includes documentation, investigation data, subject's clinical information, and all other information generated during participation in the study. No information concerning the study, or the data generated from the study will be released to any unauthorized third party without prior written approval of the DMID and the subject. Subject confidentiality will be maintained when study results are published or discussed in conferences. The study monitor, or other authorized representatives of the sponsor or governmental regulatory agencies may inspect all documents and records required to be maintained by the investigator, including but not limited to, medical records (office, clinic, or hospital) and pharmacy records for the subjects in this study. The clinical study site will permit access to such records.

All records will be kept locked, and all computer entry and networking programs will be carried out with coded numbers only and with password protected systems. All non-clinical specimens, evaluation forms, reports, and other records that leave the site will be identified only by a coded number.

Each subject will be assigned a unique study identifier. All data collection sheets will identify the subject by a unique identifier, and the date. Names will not be used on any samples or in any publication of this study. All efforts will be made to protect the privacy of subjects.

This information and data will not be used by the site principal investigator or other study personnel for any purpose other than conducting the study. These restrictions do not apply to: (1) information which becomes publicly available through no fault of the site principal investigator or other study personnel; (2) information which is necessary to disclose in confidence to an IRB solely for the evaluation of the study; (3) information which it is necessary to disclose in order to provide appropriate medical care to a study subject; or (4) study results which may be published.

9.6 Certificate of Confidentiality

To protect privacy, we have received a Certificate of Confidentiality. With this Certificate, the researchers cannot be forced to release information that may identify the research subject, even by a court subpoena, in any federal, state, or local civil, criminal, administrative, legislative, or other proceedings. The researchers will use the Certificate to resist any demands for information that would identify the subject, except as explained below.

The Certificate cannot be used to resist a demand for information from personnel of the United States Government that is used for auditing or evaluation of federally funded projects, like this study, or for information that must be released in order to meet the requirements of the Federal Food and Drug Administration (FDA).

A Certificate of Confidentiality does not prevent the subject from voluntarily releasing information about themselves or their involvement in this research. If any person or agency obtains a written consent to receive research information, then the researchers may not use the Certificate to withhold that information.

The Certificate of Confidentiality does not prevent the researchers from reporting without the subject's consent, information that would identify the subject as a participant in the research project regarding matters that must be legally reported including child and elder abuse, sexual abuse, or wanting to harm themselves or others.

The release of individual private information or specimens for other research will only occur if consent was obtained from the individual to whom the information, document, or biospecimen pertains, or for the purposes of other research that is in compliance with applicable Federal regulations governing the protection of human subjects in research.

9.7 Costs, Subject Compensation, and Research Related Injuries

There is no cost to subjects for the research tests, procedures, and study product while taking part in this trial. Procedures and treatment for clinical care may be billed to the subject, subject's insurance or third party. Subjects may be compensated for their participation in this trial.

Compensation will be in accordance with the local IRB's policies and procedures, and subject to IRB approval.

If it is determined by the site principal investigator that an injury occurred to a subject as a direct result of the tests or treatments that are done for this trial, then referrals to appropriate health care facilities will be provided to the subject. Study personnel will try to reduce, control, and treat any complications from this trial. Immediate medical treatment may be provided by the participating site. No financial compensation will be provided to the subject by the NIAID, NIH to the subject, or by the participating site for any injury suffered due to participation in this trial.

10 STATISTICAL CONSIDERATIONS

10.1 Study Hypotheses

The primary objective of this study is to estimate the combined vaccine efficacy of 2 doses of WRSs2 (10^6 cfu or $5x10^5$ cfu) in preventing shigellosis following challenge with *S. sonnei* strain 53G. Vaccine efficacy (VE) will be estimated by the prevention of shigellosis, as defined in Table 4, among vaccine recipients compared to placebo recipients through Day 63 after receipt of challenge.

$$\widehat{VE} = \frac{P_0 - P_1}{P_0}$$

P_1 is the observed proportion of shigellosis among subjects receiving WRSs2 and P_0 is the observed proportion among subjects receiving placebo.

The null hypothesis is that the absolute vaccine efficacy is zero. The one-sided alternative hypothesis is that the absolute vaccine efficacy is greater than zero.

10.2 Sample Size Considerations

Assuming a rate of 70% in the placebo arm with shigellosis after challenge, and that the expected vaccine efficacy is 57%, this corresponds to an expected rate of 30% in a WRSs2 group.

The study was originally designed with three arms (one dose of WRSs2, two doses of WRSs2, and placebo) with two primary hypotheses comparing each vaccine arm to placebo. It was

determined that 120 randomized subjects would be required to ensure at least 90 subjects were challenged, assuming approximately 25% of subjects who are randomized would drop-out or no longer qualify to receive challenge. Thirty (30) subjects per arm provided a power of ~82% for each hypothesis test, using a two-sided Chi-square test at alpha=0.025 (equivalent to a one-sided test at alpha=0.0125) utilizing a Bonferroni correction to adjust for the increase in Type I error due to having two primary hypotheses.

After the study accrued with 69 subjects, the DSMB recommended lowering the dose due to a halting event. To meet the recommendation of the DSMB and evaluate the safety and efficacy signal, the decision was made to amend the protocol to discontinue further enrollment in the one dose vaccine arm and proceed with two arms (two lower doses of WRSs2 or two doses of placebo). It is anticipated that approximately 43 subjects will be randomized to either two administrations of the lower dose or placebo to achieve approximately 30 subjects challenged.

The primary objective was modified to estimate the combined vaccine efficacy of two doses of WRSs2 at either the higher or lower dose in order to have enough power to detect vaccine efficacy with a smaller sample size than planned. Given the following assumptions:

-70% Shigellosis rate in placebo arm

-57% true vaccine efficacy of WRSs2

-One hypothesis test for the combined vaccine efficacy of 2 doses of WRSs2 (eliminating the one dose arm from the primary analysis), with the following null and alternative hypothesis:

-Null hypothesis: vaccine efficacy = 0

-Alternative hypothesis: vaccine efficacy > 0

-Placebo arm sample size: 25 (~15 from prior to study halt + ~10 after protocol amendment)

-Combined 2 dose WRSs2 arm sample size: 35 (~15 from prior to study halt + ~20 after protocol amendment)

A two-sided Chi-squared test at alpha=0.05 provides approximately 89% power to detect a combined vaccine efficacy greater than zero.

10.3 Treatment Assignment Procedures

10.3.1 Randomization Procedures

It was planned to randomize up to 120 subjects to three vaccination arms (Arm 1: WRSs2/WRSs2, Arm 2: Placebo/WRSs2, and Arm 3: Placebo/Placebo) in a 1:1:1 ratio using 10^6 cfu.

Based on recommendations of the DSMB, the dose of study vaccine was decreased to $5x10^5$ cfu WRSs2 and the design modified to 2 arms with a randomization scheme of 2:1 (vaccine: placebo). Up to 3 sites may be utilized for this study and as such treatment assignments will be stratified by site. A permuted block randomization scheme will be developed to avoid the potential for serious imbalance in the number of subjects assigned to each arm, an imbalance that can occur in the simple randomization procedures. Subjects who withdraw, or are withdrawn from the study, or are lost to follow-up after randomization will not be replaced.

The list of randomized treatment assignments will be prepared by statisticians at the Emmes Corporation and included in the enrollment module IDES for the trial. IDES will assign each subject a treatment code after demographic and eligibility data have been entered into the system. A designated individual at the site will be provided with a treatment key, which links the treatment code to the actual treatment assigned, which will be kept in a secure place.

Instructions for use of the enrollment module will be included in the IDES User's Guide. Manual back-up randomization procedures and instructions are provided for use in the event that the site temporarily loses access to the Internet, or the online enrollment system is unavailable.

Per GCP, screening records will be kept at the study site to document the reason why an individual was screened but failed trial entry criteria. The reasons why individuals failed screening will be recorded in IDES.

10.3.2 Masking Procedures

The *Shigella* vaccine and placebo will be prepared by the unblinded site pharmacist and will be administered by unblinded study staff on Day 1 and Day 29. The subjects, the study personnel who perform any study-related assessments after administration, data entry personnel at the sites, and laboratory personnel performing immunologic assays will be blinded to treatment assignment.

The DSMB may receive data in aggregate and presented by treatment group, but without the treatment group identified. The DSMB may be unblinded to individual study treatment assignments, as needed to adequately assess safety issues. Refer to the MOP for unblinding procedures.

10.4 Planned Interim Analyses

No formal interim analysis involving the testing of a hypothesis is planned.

10.4.1 Interim Safety Review

As described in Section 8.7, the DSMB will meet and review safety data at specified intervals. The study will be monitored to determine if any of the safety halting rules described in Section

8.6 is met. The halting rules do not utilize any statistical criteria and no formal hypothesis testing is planned to occur for the safety reviews.

10.4.2 Interim Immunogenicity or Efficacy Review

No interim immunogenicity or efficacy review is planned.

10.5 Final Analysis Plan

A formal SAP will be developed and finalized prior to any analysis, or database lock.

The final analysis will be performed, and clinical study report completed and distributed when all primary, secondary, and exploratory endpoint data are available. For purposes of scientific meeting presentation of study progress in advance of the final analysis, the protocol PI may request analysis of the demographics and blinded aggregate analyses of available primary and secondary endpoint data.

10.5.1 Endpoint Review Committee

For all enrolled subjects, all available clinical trial data necessary for determination of primary endpoint status (shigellosis) per Table 4 will be reviewed by a blinded endpoint review committee (ERC). This committee will be comprised primarily of independent *Shigella* experts, the study PI, and the SDCC. The ERC will be responsible for making a final determination of endpoint status for each subject prior to database lock and study unblinding[34].

10.5.2 Analysis Populations

The Safety Population includes all subjects who received at least one dose of study vaccination. Subjects will be analyzed according to the study product they actually received, not necessarily the study product to which a subject was randomized.

The Immunogenicity Population consists of all subjects who received any study product and for whom immunogenicity endpoint data are available. Subjects will be analyzed according to the study product actually received, not necessarily the study product to which a subject was randomized to.

The Full Analysis Population includes all randomized subjects with complete data for the primary efficacy endpoint. Subjects will be analyzed according to the study arm to which they were randomized.

The Per Protocol (PP) Population includes all subjects in the Full Analysis Population with the following exclusions:

- Subjects found to be ineligible at baseline.

- Subjects who received an incorrect study vaccination
- Subjects with second study vaccination or challenge received out of window
- Subjects who received any of the following prior to the assessment of the primary endpoint:
    - Non-study vaccines
    - Antibiotics
    - Oral, parenteral, or high-dose inhaled steroids
    - Immunosuppressive or cytotoxic therapy
    - Blood products or immunoglobulins
    - Experimental products

In the case of mis-randomization, subjects in the PP population will be analyzed according to the study product actually received.

10.5.3 Primary Efficacy Analysis

The primary efficacy analysis will be conducted in the Full Analysis Population and the Per Protocol Population among subjects who were randomized to receive two doses of 10^6 cfu or $5x10^5$ cfu of WRSs2 or placebo.

The combined absolute vaccine efficacy of two doses of 10^6 or $5x10^5$ of WRSs2 will be estimated and presented with a 95% confidence interval, and the null hypothesis that the absolute vaccine efficacy is zero will be tested using a two-sided Chi-squared test. If the p-value is <0.05 and the lower limit of the 95% confidence interval for VE is above zero, then we can conclude that the combined VE of two doses of WRSs2 is greater than zero.

10.5.4 Secondary Efficacy Analysis

Secondary efficacy analyses to estimate the vaccine efficacy of the individual vaccine arms (1 dose of 10^6 cfu, 2 doses of 10^6 cfu, and 2 doses of $5x10^5$ cfu) will be presented along with 95% confidence intervals. As the study is not powered for these individual comparisons to placebo, no formal hypothesis tests are planned, and no p-values will be presented. As such, no adjustments for multiplicity will be made.

10.5.5 Safety Data

Safety data will be summarized for the Safety Analysis Population. Subjects receiving a vaccination according to a study arm other than the study arm to which they were randomized, will be analyzed according to the vaccination(s) they actually received. Solicited AEs will be summarized by maximum severity for each day after each study vaccination for Days 1-8. The number, percentage (observed rate), and exact two-sided 95% confidence interval for subjects reporting each solicited AE within 8 days following vaccination will be summarized. In addition,

maximum severity for each solicited AE will be determined for each subject and reported by study arm and the resulting number and percentage of subjects will be summarized by severity grade (none, mild, moderate, severe). Summaries of solicited AEs will be presented separately by study arm for each study vaccination as well as overall study vaccinations.

Vaccine-related unsolicited AEs will be coded by Medical Dictionary for Regulatory Activities (MedDRA®) for preferred term and system organ class (SOC). The number of SAEs are likely to be small in this trial and will be reported by detailed listings showing the event description, MedDRA® preferred term and SOC, relevant dates (study vaccinations/challenge and AEs), severity, relatedness, and outcome for each event. Non-serious unsolicited vaccine-related AEs will be summarized as number and percentage of subjects reporting at least one event in each MedDRA® preferred term and SOC, cross tabulated by severity and relationship to study product. Additionally, the proportion of subjects and exact 95% confidence intervals of AEs by study arm for each study vaccination and challenge as well as overall study vaccinations and by MedDRA® categories will be calculated.

10.5.6 Immunogenicity Data

Immunogenicity data summaries and analysis will be presented for the Immunogenicity Population.

Immune responses for *Shigella* LPS-specific and Invaplex-specific IgA and IgG will be summarized at each time point by study arm. Geometric mean titers (GMTs), geometric mean fold rise (GMFR), and corresponding 95% confidence intervals will be reported. The maximum titer and peak-fold rise for the post-vaccination and post-challenge time period will be reported. The number and percentage of subjects with at least a 4-fold rise from pre-vaccination (or pre-challenge) will be reported for each post-vaccination (and post-challenge) time point.

Graphical presentations of immune response may include reverse cumulative distribution (RCD) curves and longitudinal presentation of GMTs.

10.5.7 Missing Values and Outliers

All attempts will be made to collect all data per protocol. As missing data are expected to be minimal, no imputation will be performed for missing values. Any data point that appears to be erroneous or inexplicable based on clinical judgment will be investigated as a possible outlier. If data points are identified as outliers, sensitivity analyses will be performed to examine the impact of including or excluding the outliers. Any substantive differences in these analyses will be reported.

11 SOURCE DOCUMENTS AND ACCESS TO SOURCE DATA/DOCUMENTS

Each participating site will maintain appropriate medical and research records in compliance with ICH E6, Section 4.9 and regulatory and institutional requirements for the protection of confidentiality of subjects. Each site will permit authorized representatives of the DMID, its designees, and appropriate regulatory agencies to examine (and when required by applicable law, to copy) clinical records for the purposes of quality assurance reviews, audits, and evaluation of the study safety and progress. These representatives will be permitted access to all source data and source documents, which include, but are not limited to, hospital records, clinical and office charts, laboratory notes, memoranda, pharmacy dispensing records, recorded data from automated instruments, copies or transcriptions certified after verification as being accurate and complete, microfiches, photographic negatives, microfilm or magnetic media, x-rays, and subject files and records kept at the pharmacy, at the laboratories, and medico-technical departments involved in the clinical trial.

The study uses direct data entry for the participating clinic site. Subjects will utilize a web-based e-Memory aid. Staff will review with subjects use of memory aid, how to complete a web based "e-Memory aid" and are expected to enter information in the e-Memory aid each day. Data entered by subjects into the e-Memory Aid are stored in the data system for clinic staff review during scheduled visits. The e-Memory Aid is not considered source data. After clinic staff review and save the data, the data will be entered into Advantage eClinical as source.

The site staff will be the data originators for the clinically reviewed data in Advantage eClinical® that will be used for the study endpoints.

12 QUALITY CONTROL AND QUALITY ASSURANCE

Following a written DMID-accepted site quality management plan, each participating site and its subcontractors are responsible for conducting routine quality assurance (QA) and quality control (QC) activities to internally monitor study progress and protocol compliance. The site principal investigator will provide direct access to all study-related sites, source data/data collection forms, and reports for the purpose of monitoring and auditing by the sponsor, and inspection by local

and regulatory authorities. The site principal investigator will ensure all study personnel are appropriately trained and applicable documentations is maintained on site.

The DCC will implement quality control procedures beginning with the data entry system and generate data quality control checks that will be run on the database. Any missing data or data anomalies will be communicated to the participating site(s) for clarification and resolution.

DMID-designated clinical monitors will verify that the clinical trial is conducted, and data are generated, documented (recorded), and reported in compliance with the protocol, ICH/GCP guidelines, and the applicable regulatory requirements. Clinical monitoring reports will be submitted to DMID.

13 DATA HANDLING AND RECORD KEEPING

13.1 Data Management Responsibilities

The investigator is responsible for ensuring the accuracy, completeness, legibility, and timeliness of the data reported. All source documents should be completed in a neat, legible manner to ensure accurate interpretation of data. Black or blue permanent ink is required to ensure clarity of reproduced copies. When making changes or corrections, cross out the original entry with a single line, and initial and date the change. DO NOT ERASE, OVERWRITE, OR USE CORRECTION FLUID OR TAPE ON THE ORIGINAL.

Electronic case report forms will be created by the SDCC to record and maintain data for each subject enrolled in this study.

The sponsor and/or its designee will provide guidance to the site principal investigators and other study personnel on making corrections to the data collection forms and eCRF.

13.2 Data Coordinating Center/Biostatistician Responsibilities

All electronic case report forms and laboratory reports must be reviewed by the clinical team and data entry personnel, who will ensure that they are accurate and complete. AEs must be recorded on the appropriate electronic case report form, assessed for severity and relationship, and reviewed by the site principal investigator or appropriate sub-investigator.

Data collection is the responsibility of the study personnel at the participating VTEU site under the supervision of the respective site principal investigator. During this study, the site principal investigator must maintain complete and accurate documentation for this study.

The SDCC for this study will be responsible for data management, quality review, analysis, and reporting of the study data.

13.3 Data Capture Methods

Clinical data (including, but not limited to, AE/SAEs, concomitant medications, medical history, physical assessments, and clinical laboratory values) immunogenicity, and reactogenicity will be collected by study personnel then entered into eCRFs via a 21 CFR Part 11-compliant internet data entry system provided by the study data coordinating center. The data system includes password protection and internal quality checks, such as automatic range checks, to identify data that appear inconsistent, incomplete, or inaccurate. Clinical data will be entered directly into electronic case report forms by the study personnel.

13.4 Types of Data

Data for this trial will include clinical, safety, efficacy, and outcome measures (e.g., assessments, clinical laboratory values, reactogenicity, and immunogenicity data).

13.5 Study Records Retention

Study records and reports including, but not limited to, eCRFs, source documents, ICFs, laboratory test results, and study drug disposition records will be retained for 2 years after a marketing application is approved for the study product for the indication for which it is being investigated; or, if no application is to be filed or if the application is not approved for the study product, until 2 years after the investigation is discontinued and the FDA has been notified. These documents will be retained for a longer period, however, if required by local regulations. ICFs for secondary research will be maintained if the sample/specimen exists.

No records will be destroyed without the written consent of the sponsor. The sponsor is responsible for informing the site's principal investigator when these documents no longer need to be retained. The participating VTEU site must contact DMID for authorization prior to the destruction of any study records.

14 CLINICAL MONITORING

Site monitoring is conducted to ensure that the human subjects' protections, study and laboratory procedures, study intervention administration, and data collection processes are of high quality and meet sponsor, ICH/GCP guidelines and applicable regulations, and that this trial is conducted in accordance with the protocol, protocol-specific MOP, and applicable sponsor

standard operating procedures. DMID, the sponsoring agency, or its designee will conduct site-monitoring visits as detailed in the clinical monitoring plan.

Site visits will be made at standard intervals as defined by DMID and may be made more frequently as directed by DMID. Monitoring visits will include, but is not limited to, review of regulatory files, accountability records, eCRFs, informed consent forms, medical and laboratory reports, and protocol and GCP compliance. Site monitors will have access to each participating site, study personnel, and all study documentation according to the DMID-approved site monitoring plan. Study monitors will meet with site principal investigators to discuss any problems and actions to be taken and will document site visit findings and discussions.

15 PUBLICATION POLICY

Following completion of the study, the lead Principal Investigator will publish the results of this research in a scientific journal. The Principal Investigators will submit or have submitted for them to the National Library of Medicine's PubMed Central (http://www.ncbi.nlm.nih.gov/pmc/) an electronic version of their final, peer-reviewed manuscripts upon acceptance for publication, to be made publicly available no later than 12 months after the official date of publication in accordance with:

NIH Public Access Policy, http://publicaccess.nih.gov/

NIH Office of Extramural Research (OER) Grants and Funding, http://grants.nih.gov/grants/oer.htm

As of January 2018, all clinical trials supported by the NIH must be registered on ClinicalTrials.gov, no later than 21 days after the enrollment of the first subject. Results of all clinical trials supported by the NIH, generally, need to be submitted no later than 12 months following the primary completion date. A delay of up to 2 years is available for trials that meet certain criteria and have applied for certification of delayed posting.

As part of the result posting a copy of this protocol (and its amendments) and a copy of the Statistical Analysis Plan will be posted on ClincialTrials.gov.

For this trial the responsible party is DMID which will register the trial and post results.

The responsible party, DMID, does not plan to request certification of delayed posting.

Refer to:

Public Law 110-85, Section 801, Clinical Trial Databases

42CFR11

NIH NOT-OD-16-149

16 LITERATURE REFERENCES

1. Collaborators GBDDD. Estimates of global, regional, and national morbidity, mortality, and aetiologies of diarrhoeal diseases: a systematic analysis for the Global Burden of Disease Study 2015. Lancet Infect Dis. 2017;17(9):909-48. doi: 10.1016/S1473-3099(17)30276-1. PubMed PMID: 28579426; PubMed Central PMCID: PMCPMC5589208.

2. Kotloff KL, Nataro JP, Blackwelder WC, Nasrin D, Farag TH, Panchalingam S, et al. Burden and aetiology of diarrhoeal disease in infants and young children in developing countries (the Global Enteric Multicenter Study, GEMS): a prospective, case-control study. Lancet. 2013;382(9888):209-22. doi: 10.1016/S0140-6736(13)60844-2. PubMed PMID: 23680352.

3. Ashkenazi S. Shigella infections in children: new insights. Semin Pediatr Infect Dis. 2004;15(4):246-52. PubMed PMID: 15494948.

4. Lamberti LM, Bourgeois AL, Fischer Walker CL, Black RE, Sack D. Estimating diarrheal illness and deaths attributable to Shigellae and enterotoxigenic Escherichia coli among older children, adolescents, and adults in South Asia and Africa. PLoS Negl Trop Dis. 2014;8(2):e2705. doi: 10.1371/journal.pntd.0002705. PubMed PMID: 24551265; PubMed Central PMCID: PMCPMC3923718.

5. Wilson R, Feldman RA, Davis J, LaVenture M. Family illness associated with Shigella infection: the interrelationship of age of the index patient and the age of household members in acquisition of illness. J Infect Dis. 1981;143(1):130-2. PubMed PMID: 7217710.

6. Shakoor S, Zaidi AK, Hasan R. Tropical bacterial gastrointestinal infections. Infect Dis Clin North Am. 2012;26(2):437-53. doi: 10.1016/j.idc.2012.02.002. PubMed PMID: 22632648.

7. Kotloff KL, Winickoff JP, Ivanoff B, Clemens JD, Swerdlow DL, Sansonetti PJ, et al. Global burden of Shigella infections: implications for vaccine development and implementation of control strategies. Bull World Health Organ. 1999;77(8):651-66. PubMed PMID: 10516787; PubMed Central PMCID: PMCPMC2557719.

8. Ericsson CD. Travelers' diarrhea. Epidemiology, prevention, and self-treatment. Infect Dis Clin North Am. 1998;12(2):285-303. PubMed PMID: 9658246.

9. Kosek M, Yori PP, Olortegui MP. Shigellosis update: advancing antibiotic resistance, investment empowered vaccine development, and green bananas. Curr Opin Infect Dis. 2010;23(5):475-80. doi: 10.1097/QCO.0b013e32833da204. PubMed PMID: 20689423.

10. Niyogi SK. Increasing antimicrobial resistance--an emerging problem in the treatment of shigellosis. Clin Microbiol Infect. 2007;13(12):1141-3. doi: 10.1111/j.1469-0691.2007.01829.x. PubMed PMID: 17953700.

11. Shiferaw B, Solghan S, Palmer A, Joyce K, Barzilay EJ, Krueger A, et al. Antimicrobial susceptibility patterns of Shigella isolates in Foodborne Diseases Active Surveillance Network (FoodNet) sites, 2000-2010. Clin Infect Dis. 2012;54 Suppl 5:S458-63. doi: 10.1093/cid/cis230. PubMed PMID: 22572670.

12. Bernardini ML, Mounier J, d'Hauteville H, Coquis-Rondon M, Sansonetti PJ. Identification of icsA, a plasmid locus of Shigella flexneri that governs bacterial intra- and intercellular spread through interaction with F-actin. Proc Natl Acad Sci U S A. 1989;86(10):3867-71. PubMed PMID: 2542950; PubMed Central PMCID: PMCPMC287242.

13. Makino S, Sasakawa C, Kamata K, Kurata T, Yoshikawa M. A genetic determinant required for continuous reinfection of adjacent cells on large plasmid in S. flexneri 2a. Cell. 1986;46(4):551-5. PubMed PMID: 3524856.

14. Collins TA, Barnoy S, Baqar S, Ranallo RT, Nemelka KW, Venkatesan MM. Safety and colonization of two novel VirG(IcsA)-based live Shigella sonnei vaccine strains in rhesus macaques (Macaca mulatta). Comp Med. 2008;58(1):88-94. PubMed PMID: 19793462; PubMed Central PMCID: PMCPMC2703165.

15. Venkatesan MM, Ranallo RT. Live-attenuated Shigella vaccines. Expert Rev Vaccines. 2006;5(5):669-86. Epub 2006/12/22. doi: 10.1586/14760584.5.5.669. PubMed PMID: 17181440.

16. Kotloff KL, Taylor DN, Sztein MB, Wasserman SS, Losonsky GA, Nataro JP, et al. Phase I evaluation of delta virG Shigella sonnei live, attenuated, oral vaccine strain WRSS1 in healthy adults. Infect Immun. 2002;70(4):2016-21. PubMed PMID: 11895966; PubMed Central PMCID: PMCPMC127867.

17. Orr N, Katz DE, Atsmon J, Radu P, Yavzori M, Halperin T, et al. Community-based safety, immunogenicity, and transmissibility study of the Shigella sonnei WRSS1 vaccine in Israeli volunteers. Infect Immun. 2005;73(12):8027-32. doi: 10.1128/IAI.73.12.8027-8032.2005. PubMed PMID: 16299296; PubMed Central PMCID: PMCPMC1307051.

18. Pitisuttithum P, Islam D, Chamnanchanunt S, Ruamsap N, Khantapura P, Kaewkungwal J, et al. Clinical Trial of an Oral Live Shigella sonnei Vaccine Candidate, WRSS1, in Thai Adults. Clin Vaccine Immunol. 2016;23(7):564-75. doi: 10.1128/CVI.00665-15. PubMed PMID: 27146000; PubMed Central PMCID: PMCPMC4933782.

19. Nataro JP, Seriwatana J, Fasano A, Maneval DR, Guers LD, Noriega F, et al. Identification and cloning of a novel plasmid-encoded enterotoxin of enteroinvasive Escherichia coli and Shigella strains. Infect Immun. 1995;63(12):4721-8. Epub 1995/12/01. PubMed PMID: 7591128; PubMed Central PMCID: PMCPMC173677.

20. Venkatesan MM, Goldberg MB, Rose DJ, Grotbeck EJ, Burland V, Blattner FR. Complete DNA sequence and analysis of the large virulence plasmid of Shigella flexneri. Infect Immun. 2001;69(5):3271-85. Epub 2001/04/09. doi: 10.1128/IAI.69.5.3271-3285.2001. PubMed PMID: 11292750; PubMed Central PMCID: PMCPMC98286.

21. Faruque SM, Khan R, Kamruzzaman M, Yamasaki S, Ahmad QS, Azim T, et al. Isolation of Shigella dysenteriae type 1 and S. flexneri strains from surface waters in Bangladesh: comparative molecular analysis of environmental Shigella isolates versus clinical strains. Appl Environ Microbiol. 2002;68(8):3908-13. Epub 2002/07/31. doi: 10.1128/aem.68.8.3908-3913.2002. PubMed PMID: 12147489; PubMed Central PMCID: PMCPMC124020.

22. Fasano A, Noriega FR, Liao FM, Wang W, Levine MM. Effect of shigella enterotoxin 1 (ShET1) on rabbit intestine in vitro and in vivo. Gut. 1997;40(4):505-11. Epub 1997/04/01. doi: 10.1136/gut.40.4.505. PubMed PMID: 9176079; PubMed Central PMCID: PMCPMC1027126.

23. Mou X, Souter S, Du J, Reeves AZ, Lesser CF. Synthetic bottom-up approach reveals the complex interplay of Shigella effectors in regulation of epithelial cell death. Proc Natl Acad Sci U S A. 2018;115(25):6452-7. doi: 10.1073/pnas.1801310115. PubMed PMID: 29866849; PubMed Central PMCID: PMCPMC6016774.

24. Kotloff KL, Pasetti MF, Barry EM, Nataro JP, Wasserman SS, Sztein MB, et al. Deletion in the Shigella enterotoxin genes further attenuates Shigella flexneri 2a bearing guanine auxotrophy in a phase 1 trial of CVD 1204 and CVD 1208. J Infect Dis. 2004;190(10):1745-54. doi: 10.1086/424680. PubMed PMID: 15499528.

25. Frenck RW, Jr., Baqar S, Alexander W, Dickey M, McNeal M, El-Khorazaty J, et al. A Phase I trial to evaluate the safety and immunogenicity of WRSs2 and WRSs3; two live oral candidate vaccines against Shigella sonnei. Vaccine. 2018;36(32 Pt B):4880-9. Epub 2018/07/25. doi: 10.1016/j.vaccine.2018.06.063. PubMed PMID: 30037478.

26. Islam D, Ruamsap N, Khantapura P, Aksomboon A, Srijan A, Wongstitwilairoong B, et al. Evaluation of an intragastric challenge model for Shigella dysenteriae 1 in rhesus monkeys (Macaca mulatta) for the pre-clinical assessment of Shigella vaccine formulations. APMIS. 2014;122(6):463-75. doi: 10.1111/apm.12168. PubMed PMID: 24028276; PubMed Central PMCID: PMCPMC3954967.

27. Gregory M, Kaminski RW, Lugo-Roman LA, Galvez Carrillo H, Tilley DH, Baldeviano C, et al. Development of an Aotus nancymaae model for Shigella Vaccine immunogenicity and efficacy studies. Infect Immun. 2014;82(5):2027-36. doi: 10.1128/IAI.01665-13. PubMed PMID: 24595138; PubMed Central PMCID: PMCPMC3993435.

28. Porter CK, Thura N, Ranallo RT, Riddle MS. The Shigella human challenge model. Epidemiol Infect. 2013;141(2):223-32. doi: 10.1017/S0950268812001677. PubMed PMID: 22906296.

29. Frenck RW, Jr., Dickey M, Suvarnapunya AE, Chandrasekaran L, Kaminski RW, Clarkson KA, et al. Establishment of a Controlled Human Infection Model with a Lyophilized Strain of Shigella sonnei 53G. mSphere. 2020;5(5). Epub 2020/09/25. doi:

10.1128/mSphere.00416-20. PubMed PMID: 32968005; PubMed Central PMCID: PMCPMC7568646.

30. Thabane M, Kottachchi DT, Marshall JK. Systematic review and meta-analysis: The incidence and prognosis of post-infectious irritable bowel syndrome. Aliment Pharmacol Ther. 2007;26(4):535-44. doi: 10.1111/j.1365-2036.2007.03399.x. PubMed PMID: 17661757.

31. Neal KR, Hebden J, Spiller R. Prevalence of gastrointestinal symptoms six months after bacterial gastroenteritis and risk factors for development of the irritable bowel syndrome: postal survey of patients. BMJ. 1997;314(7083):779-82. PubMed PMID: 9080994; PubMed Central PMCID: PMCPMC2126206.

32. Talaat KR, Bourgeois AL, Frenck RW, Chen WH, MacLennan CA, Riddle MS, et al. Consensus Report on Shigella Controlled Human Infection Model: Conduct of Studies. Clin Infect Dis. 2019;69(Suppl 8):S580-S90. Epub 2019/12/10. doi: 10.1093/cid/ciz892. PubMed PMID: 31816068; PubMed Central PMCID: PMCPMC6901126.

33. Barnoy S, Jeong KI, Helm RF, Suvarnapunya AE, Ranallo RT, Tzipori S, et al. Characterization of WRSs2 and WRSs3, new second-generation virG(icsA)-based Shigella sonnei vaccine candidates with the potential for reduced reactogenicity. Vaccine. 2010;28(6):1642-54. Epub 2009/11/26. doi: 10.1016/j.vaccine.2009.11.001. PubMed PMID: 19932216; PubMed Central PMCID: PMCPMC2999844.

34. MacLennan CA, Riddle MS, Chen WH, Talaat KR, Jain V, Bourgeois AL, et al. Consensus Report on Shigella Controlled Human Infection Model: Clinical Endpoints. Clin Infect Dis. 2019;69(Suppl 8):S591-S5. Epub 2019/12/10. doi: 10.1093/cid/ciz891. PubMed PMID: 31816065; PubMed Central PMCID: PMCPMC6901125.

17 APPENDICES

Appendix A: Schedule of Events

Appendix A 1: Screening and Vaccination

Appendix A 2: Inpatient Challenge and Follow-up

Appendix B: Toxicity Table/Laboratory AE Grading Scale

Appendix C: Vital Signs AE Grading Scale

Appendix D: Diarrhea Classification

Appendix E: Reactogenicity Grading Scale

Appendix A: Schedule of Events

Appendix A 1: Screening and Vaccination

Study Event										
Visit Number	00A	00B	01	03	05	06	07	09	11	12
Study Day	Screen -60 to-3	-3	1	4	8	15	29	32	36	43
Compliance range days		-2		±1	±1	±2	+2	±1	±1	±2
Day from Last vaccination			0	3	7	14	28	3	7	14
Vaccination WRSs2			X[5]				X[5]			
Contact Type	C	C	C	C	C	C	C	C	C	C
Inclusion / Exclusion Criteria review	✓		✓							
Dose Qualification							✓			
Educational Materials, Written Test	✓	(✓)								
Informed Consent	✓									
Demographics	✓									
Abbreviated PE	✓									
Targeted PE			¶	¶	¶	¶	¶	¶	¶	¶
Vital Sign[^, 1, 6]	✓[11]		✓	¶	¶	¶	✓	¶	¶	¶
Concomitant Medication	✓		✓	¶	✓	✓	✓	¶	✓	✓
Medical History	✓									

Study Event										
Visit Number	00A	00B	01	03	05	06	07	09	11	12
Study Day	Screen -60 to-3	-3	1	4	8	15	29	32	36	43
Compliance range days		-2		±1	±1	±2	+2	±1	±1	±2
Day from Last vaccination			0	3	7	14	28	3	7	14
Vaccination WRSs2			X[5]				X[5]			
Contact Type	C	C	C	C	C	C	C	C	C	C
Interim Medical History			✓	¶	✓	✓	✓	¶	✓	✓
Urine Pregnancy	✓		✓				✓			
Urine Dipstick, Opioid Testing	✓									
Screening health /safety blood[%], mL	11									
Safety Lab	6.5				6.5				6.5	
Reactogenicity Memory Aid			✓[2]	✓[3]	✓[3]		✓[2]	✓[3]	✓[3]	
Collection of AEs, SAEs, other illness, pregnancy			✓	✓	✓	✓	✓	✓	✓	✓
Serum IgA & IgG (mL blood)	5	10	(10)			10	10			10
ASC Whole Blood, mL		16	(16)		16		16		16	
Stool collection for WRSs2 shedding		✓	(✓)	✓	✓	✓	✓	✓	✓	✓
Stool for immunoblot and PCR		✓	(✓)	✓	✓	✓	✓	✓	✓	✓
Stool for IgA		✓	(✓)		✓	✓	✓		✓	✓
**Samples for Secondary research[?]:**										
Plasma		✓	(✓)	✓	✓	✓	✓	✓	✓	✓

Study Event											
Visit Number	00A	00B	01	03	05	06	07	09	11	12	
Study Day	Screen -60 to-3	-3	1	4	8	15	29	32	36	43	
Compliance range days		-2		±1	±1	±2	+2	±1	±1	±2	
Day from Last vaccination			0	3	7	14	28	3	7	14	
Vaccination WRSs2			X[5]				X[5]				
Contact Type	C	C	C	C	C	C	C	C	C	C	
Whole Blood PBMC for Tfh (FACS T/B/IR Cell Panel) and Phenotyping, mL		16	(16)	16			16	16			
**Paxgene Whole Blood, mL**		2.5	(2.5)	2.5	2.5		2.5	2.5	2.5		
Blood for serum (IgG subclass, Avidity/affinity, Microarray)		5	(5)			5	5			5	
ALS whole blood		24	(24)		24		24		24		
Memory B cell Whole blood, mL		16	(16)				16				
SBA		✓	(✓)			✓	✓			✓	
Stool Microbiome		✓	(✓)	✓	✓	✓	✓	✓	✓	✓	
**Estimated Total Blood Volume (Vaccination Phase) mL**	22.5	89.5	(89.5)	18.5	49	15	89.5	18.5	49	15	366.5

Appendix A 2: Inpatient Challenge and Follow-up

Study Event														
Visit Number	13	14	15	16	17	18	19	20	21	22	23	24	25	26
Study Day	56+	57	58	59	60	61	62	63	64	65	71	85	113	180
Compliance rage days	-	±2	-	-	-	-	-	-	-	-	±2	±2	±4	±14
Day from Last vaccination	27	28												
Day from Challenge			1	2	3	4	5	6	7	8	14	28	56	
Admission	✓	¶												
Challenge with 53G		✓												
Contact Type	Inpatient Period										C	C	C	SC
Assessing safety for challenge receipt	✓													Post-Study Safety Assessment, Clinical Phase study Completion
Dose Qualification	✓													
Medical Interview	✓													
Informed Consent														
Abbreviated PE	✓	¶												
Targeted PE			¶	¶	¶	¶	¶	¶	¶	¶	¶	¶	¶	
Vital Sign^, 6 Body weight (pre-challenge and as indicated)	✓	✓	✓	✓	✓	✓	✓	✓	✓	✓	¶	¶	¶	
Concomitant Medication[10]	✓	✓	✓	✓	✓	✓	✓	✓	✓	✓	✓	✓		
Medical History	✓	¶	¶	¶	¶	¶	¶	¶	¶	¶	✓	✓	✓	
Urine Pregnancy	✓	¶												

SARS-CoV2 testing[9]	✓	¶	¶	¶	¶	¶	¶	¶	¶	¶				
Safety Lab	6.5								6.5		6.5	¶	¶	
Memory Aid										✓[2]	✓[3]			
Collection of AEs, SAEs, other illness, pregnancy	✓	✓	✓	✓	✓	✓	✓	✓	✓	✓	✓	✓	✓	
Study Event														
Visit Number	13	14	15	16	17	18	19	20	21	22	23	24	25	26
Study Day	56[+]	57	58	59	60	61	62	63	64	65	71	85	113	180
Compliance rage days	-	±2	-	-	-	-	-	-	-	-	±2	±2	±4	±14
Day from Last vaccination	27	28												
Day from Challenge			1	2	3	4	5	6	7	8	14	28	56	
Admission	✓	¶												
Challenge with 53G		✓												
Contact Type	Inpatient Period										C	C	C	SC
Reactogenicity assessment and Illness labs if needed[7],		✓	✓	✓	✓	✓	✓	✓	✓	✓	✓	✓	✓	Post-Study Safety Assessment, Clinical Phase study Completion
Stool for classification of consistency, and hemoccult[#]	✓	✓	✓	✓	✓	✓	✓	✓	✓	✓				
Measure and Record emesis output[8]		✓	✓	✓	✓	✓	✓	✓	✓	✓				
Serum IgA & IgG, (mL Blood)	10								10		10	10	10	
Antibiotic Therapy[!] Day 62-64 and discharge[@] Day 65							✓	✓	✓	@				
ASC Whole Blood, mL	16				16				16					
Stool collection for shedding	✓	✓	✓	✓	✓	✓	✓	✓	✓	✓				
Stool for immunoblot and PCR		✓	✓	✓	✓	✓	✓	✓	✓	✓				
Stool for IgA	✓					✓			✓		✓	✓		
**Samples for Secondary research**[?]														

Plasma	✓				✓				✓			✓	✓	
Whole Blood PBMC for Tfh (FACS T/B/IR Cell Panel) and Phenotyping, mL	16				16									
Paxgene whole blood, mL	2.5				2.5				2.5					

Study Event															
Visit Number	13	14	15	16	17	18	19	20	21	22	23	24	25	26	
Study Day	56+	57	58	59	60	61	62	63	64	65	71	85	113	180	
Compliance rage days	-	+2	-	-	-	-	-	-	-	-	±2	±2	±4	±14	
Day from Last vaccination	27	28													
Day from Challenge			1	2	3	4	5	6	7	8	14	28	56		
Admission	✓	¶													
Challenge with 53G		✓													
Contact Type	Inpatient Period										C	C	C	SC	
Blood for serum (IgG subclass, Avidity/affinity, Microarray)	5								5		5	65*	5		
ALS Whole blood	24				24				24						
Memory B-cell Whole blood, mL	16											16	16		
SBA	✓								✓		✓	✓	✓		
Stool Microbiome and Cytokine	✓	✓	✓	✓	✓	✓	✓	✓	✓	✓	✓		✓		
**Estimated Total Blood Volume (Challenge Phase), mL**	96				58.5				64		21.5	91	31		759.5

C = Clinic; SC = Contact

¶ = if indicated

^ Vital signs include temperature, blood pressure, and pulse

% Screening health and safety labs include: hematology (white cell count, hemoglobin, platelets, absolute neutrophil count/ANC); chemistry (total bilirubin, ALT, creatinine, sodium, potassium); serology (HIV antibodies, HBsAg, and HCV antibodies, HLA-B27, *S. sonnei* LPS IgG (ELISA)).

+ Day 56 is admission to the inpatient isolation unit 1 day prior to challenge

# All stools will be collected and visually assessed for consistency, blood, or mucus. Stools that are classified as 3-5 consistency will be weighed, and if there is visible blood, a Hemoccult test will be performed to confirm presence of occult blood. Up to two stools with blood present by visual inspection in a 24- hour period will be tested by Hemoccult. Any remaining stool will be disinfected with bleach in the plastic stool collector and discarded in the hazardous waste.

@ Discharge, continue daily protocol collections if subject does not meet discharge criteria and remains in inpatient isolation facility

* 65 mL: 5mL blood collected for secondary research; 60 mL blood will be collected to establish the repository for positive control for future assays.

! = On the 5th day after challenge (Day 62), subjects will begin to receive 500 mg of ciprofloxacin twice daily for 3 days. Subjects with contraindication to ciprofloxacin may receive as second-line treatment trimethoprim-sulfamethoxazole (160 mg/800 mg twice daily for 5 days), or any antibiotic that is deemed suitable according to local guidelines and the investigator's assessment can be used.

? = These are assays that may be performed.

( ) = collected on day of vaccination or second screening visit if not collected during initial screening visit
1 = Pre-vaccination vital signs and post vaccination dose if indicated
2 = review use of memory aid, provide thermometer and instruct to **contact** site for AEs, changes to health, emergency room visit or hospitalization, SAE, or pregnancy
3 = review and confirm information from memory aid
4 = **contact** by various methods, including email, text, or phone call to remind subject to complete memory aid and instruct how to fill out memory aid
5 = vaccination assessment completed at least 90 minutes post dose
6 = Pre-challenge vital signs, post challenge dose per protocol +90 min, daily and additional assessment as indicated
7 = Illness Labs collected per PI discretion include: serum chemistry, sodium, potassium, blood urea nitrogen/BUN, and creatinine. Urine dip for specific gravity, as indicated
8 = All emesis will be measured and recorded then disinfected with bleach and discarded in the hazardous waste
9 = A SARS-CoV-2 test will be performed on all subjects upon entry into the challenge unit. Anyone with a positive test will be quarantined in their room until they can be safely escorted out of the challenge unit. In addition, a SARS-CoV-2 test will be performed at any time a subject were to exhibit symptoms of COVID-19 illness during the inpatient stay.
10 = All concomitant medications, taken in the 30 days prior to study enrollment through Day 28 following challenge or early termination, whichever occurs first, will be recorded.
11= Height and weight collected to determine BMI at screening

Appendix B: Toxicity Table/Laboratory AE Grading Scale

AE Grading

This protocol is CDISC compliant. The FDA Toxicity Grading Scale for Laboratory Abnormalities below serves as a guideline. - Institutional normal reference ranges should be provided to demonstrate that they are appropriate. During screening, subjects will have blood drawn to determine if any clinically significant laboratory abnormalities exist that would preclude study participation. Following enrollment, the toxicity table will be used as a reference for grading severity of laboratory AEs.

	**Mild (Grade 1)**	**Moderate (Grade 2)**	**Severe (Grade 3)**
**Serum**			
Sodium – Hyponatremia mEq/L	132 – 134	130 – 131	<130
Sodium – Hypernatremia mEq/L	144 – 145	146 – 147	>147
Potassium – Hyperkalemia mEq/L	5.1 – 5.2	5.3 – 5.4	>5.4
Potassium – Hypokalemia mEq/L	3.5 – 3.6	3.3 – 3.4	< 3.3
Blood Urea Nitrogen BUN	23 – 26	27 – 31	> 31
Creatinine – mg/dL	1.5 – 1.7	1.8 – 2.0	> 2.0
Liver Function Tests ALT increase by factor	1.1 – 2.5 x ULN**	2.6 – 5.0 x ULN	>5.0 x ULN
Bilirubin – when Liver Function Test is normal; increase by factor	1.1 – 1.5 x ULN	1.6 – 2.0 x ULN	>2.0 x ULN
**Hematology**			
Hemoglobin (Female) - gm/dL	11.0 – 12.0	9.5 – 10.9	< 9.5
Hemoglobin (Male) - gm/dL	12.5 – 13.5	10.5 – 12.4	< 10.5
WBC Increase - cell/mm^3	10,800 – 15,000	15,001 – 20,000	> 20, 000
WBC Decrease - cell/mm^3	2,500 – 3,500	1,500 – 2,499	< 1,500
Neutrophils Decrease - cell/mm^3	1,500 – 2,000	1,000 – 1,499	< 1,000
Platelets Decreased - cell/mm^3	125,000 – 140,000	100,000 – 124,000	< 100,000
**Urine**			
Protein	Trace	1+	2+

** ULN" is the upper limit of the normal range.

Appendix C: Vital Signs ADVERSE EVENT Grading Scale

**Vital Signs Grading**		
**AE**	**Severity**	**Parameter**
Fever	1	38.0 – 38.4^{0}C<br>100.4 – 101.1^{0}F
	2	38.5 – 38.9^{0}C<br>101.2 – 102.0^{0}F
	3	≥39.0^{0}C<br>>102^{0}F or ER visit or hospitalization
Hypertension (systolic)	1	141-150 mm Hg
	2	151-155 mm Hg
	3	>155mm Hg
Hypertension (diastolic)	1	91 – 95 mm Hg
	2	96-100 mm Hg
	3	>100 mm Hg
Hypotension (systolic)	1	85-89 mm Hg
	2	80-84 mm Hg
	3	<80 mm Hg
Hypotension (diastolic)	1	50-54 mm Hg
	2	45-49 mm Hg
	3	<45 mm Hg
Bradycardia	1	45-49 bpm
	2	40-44 bpm
	3	<40 bpm
Tachycardia	1	101-115 bpm
	2	116-130 bpm

Vital Signs Grading		
**AE**	**Severity**	**Parameter**
	3	>130 bpm

Appendix D: Diarrhea Classification

Diarrheal stools are loose or watery stools classified as 3-5 consistency. To assess diarrhea, study personnel will use the following definitions for classification of stool samples:

1 (Normal stool) – Firm, tootsie roll consistency

2 (Soft stool) - Pudding consistency, not firm but holds some shape

3 (Loose stool) – Takes the shape of the container, thick gravy consistency/brown watery, opaque watery, chocolate milk consistency

4 (Watery stool) – Opaque colored, watery consistency

5 (“Rice water”) - soapy water consistency

Episode of diarrhea- begins with the first 3 or higher stool classification that ultimately is part of a diarrhea episode and ends when the subject has not passed any stool classified as 3-5 for 24 hours

Only stools classified as 3, 4 and 5 consistency will be considered in the criteria for diarrhea. Stools classified as consistency of 3, 4 and 5 within any 24-hour period will be added to determine the highest severity for the episode. When there are no stools classified as 3-5 consistency for 24 hours, the episode will be considered resolved. The end date for the episode will be the date and time of the last stool consistency was classified as 3, 4, or 5. For subjects who experience diarrhea, all stools classified as 3-5 consistency will be weighed and evaluated by qualified medical personnel for grossly visible blood.

Dysentery- ≥2 stools classified as consistency of 3-5 with gross blood (hemoccult positive) in 24 hours with one or more of the following: oral temperature ≥38.0˚C; ≥1 moderate constitutional/enteric symptom; ≥2 episodes of vomiting in 24 hours.

See Appendix E for diarrhea grading scale.

Appendix E: Reactogenicity Grading Scale

Clinical Feature	Grade 0	Grade 1 (mild)	Grade 2 (moderate)	Grade 3 (severe)
Fever	none	100.4 – 101.1 °F<br>38.0 – 38.4 °C	101.2 – 102.0 °F<br>38.5 – 38.9 °C	>102 °F/≥39.0 °C or ER visit or hospitalization
Headache	none	no pain medications taken	use of pain medication required	narcotic pain med required and/or prevents daily activity
Arthralgia	none	mild*	moderate**	severe***
Nausea	none	mild*	moderate**	severe***
Pain/Abdominal Cramps	none	mild*	moderate**	severe***
Myalgia	none	mild*	moderate**	severe***
Malaise, fatigue	none	mild*	moderate**	severe***
Anorexia, loss of appetite	none	mild*	moderate**	severe***
Chills	none	mild*	moderate**	severe***
Diarrhea	< 2 loose or watery stools of consistency 3-5	2-3 loose or watery stools consistency of 3-5 and <400 gm per 24 hours	4-5 loose or watery stools consistency of 3-5 or 400-800 gm stools classified as consistency of 3-5 per 24 hours	≥6 loose or watery stools consistency of 3-5 or >800 gm stools classified as consistency of 3-5 per 24 hours
Vomiting	none	1-2 episodes within 24-hour period	3-5 episodes within 24-hour period	>5 episodes within 24-hour period

Scoring guidelines:
***Mild**: aware of symptom, but easily tolerated
****Moderate**: discomfort enough to interfere with daily activities if at home
*****Severe**: incapacitating, unable to perform daily activities, would seek medical attention if at home
